# Antenatal and intrapartum interventions for reducing caesarean section, promoting vaginal birth, and reducing fear of childbirth: An overview of systematic reviews

**DOI:** 10.1371/journal.pone.0224313

**Published:** 2019-10-24

**Authors:** Valerie Smith, Louise Gallagher, Margaret Carroll, Kathleen Hannon, Cecily Begley

**Affiliations:** School of Nursing & Midwifery, Trinity College Dublin, Dublin, Ireland; University of the Witwatersrand, SOUTH AFRICA

## Abstract

Concern has been expressed globally over rising caesarean birth rates. Recently, the International Federation of Gynaecology and Obstetrics (FIGO) called for help from governmental bodies, professional organisations, women’s groups, and other stakeholders to reduce unnecessary caesareans. As part of a wider research initiative, we conducted an overview of systematic reviews of antenatal and intrapartum interventions, and reports of evidence based recommendations, to identify and highlight those that have been shown to be effective for reducing caesarean birth, promoting vaginal birth and reducing fear of childbirth. Following registration of the review protocol, (PROSPERO 2018 CRD42018090681), we searched The Cochrane Database of Systematic Reviews, PubMed, CINAHL and EMBASE (Jan 2000-Jan 2018) and searched for grey literature in PROSPERO, and on websites of health professional and other relevant bodies. Screening and selection of reviews, quality appraisal using AMSTAR-2, and data extraction were performed independently by pairs of at least two reviewers. Excluding reviews assessed as ‘critically low’ on AMSTAR-2 (n = 54), 101 systematic reviews, and 10 reports of evidence based recommendations were included in the overview. Narrative synthesis was performed, due to heterogeneity of review methodology and topics. The results highlight twenty-five interventions, across 17 reviews, that reduced the risk of caesarean, nine interventions across eight reviews that increased the risk of caesarean, eight interventions that reduced instrumental vaginal birth, four interventions that increased spontaneous vaginal birth, and two interventions that reduced fear of childbirth. This overview of reviews identifies and highlights interventions that have been shown to be effective for reducing caesarean birth, promoting vaginal births and reducing fear of childbirth. In recognising that clinical practices change over time, this overview includes reviews published from 2000 onwards only, thus providing contemporary evidence, and a valuable resource for clinicians when making decisions on practices that should be implemented for reducing unnecessary caesarean births safely.

**Protocol Registration:** PROSPERO 2018 CRD42018090681. Available from: http://www.crd.york.ac.uk/PROSPERO/display_record.php?ID=CRD42018090681

## Introduction

Caesarean birth can be a necessary emergency procedure to prevent maternal or neonatal harm or death. Contrastingly, caesarean birth can result in death or serious morbidity, with evidence to suggest that caesarean birth more than trebles the risk of maternal mortality (3.6 in vaginal births compared with 13.3 in caesareans, per 100,000 births), although overall rates are very low [1]. Severe maternal morbidity is also increased in caesarean birth, including a five-fold increase in cardiac arrest (0.19% versus 0.04%), 2.5 times the rate of anaesthetic complications (0.53% versus 0.21%), a trebling of infection rates (0.6% versus 0.21%), almost four times the rate of wound haematoma (1.3% versus 0.27%), and three times the rate of haemorrhage leading to hysterectomy (0.03% versus 0.01%) [2]. Neonatal respiratory distress requiring oxygen therapy is also more common in term babies if birth is by elective or emergency caesarean (35.5 and 12.2 per 1000 live births) compared with vaginal birth (5.3 per 1000 live births) [3]. Women giving birth by caesarean also have more negative perceptions of their birth experience, exhibit poorer parenting behaviours and may be at higher risk for postpartum mood disturbance [4]. In addition, caesarean birth costs €739 (elective) or €1180 (emergency) more than vaginal birth [5].

The 2013 European Perinatal Health Report records “a longstanding and continuing cause for concern” over increasing caesarean rates, with European rates varying from 16.1% in Iceland to 56.9% in Cyprus [6] and similar high rates are seen in Australia (30%), United States (33%), and Brazil (55%) [7]. Low-income countries, however, have lower rates, possibly indicating a need to increase access to hospital care, which may lead to a life-saving caesarean. A recent systematic review of 34 studies from 20 countries found that, in clinicians’ opinion, key factors influencing their decision-making for caesarean section included their personal beliefs, fear of litigation, and convenience [8]. Similar work in Sweden, where caesarean section rates are low, found that ‘belief in normal birth’ was the core theme emerging from clinicians’ accounts of their decision-making [9], in stark contrast to the review of evidence from countries with higher caesarean section rates. One factor that clinicians believe increases the caesarean rate is ‘maternal request,’ often presumed to be due to fear of childbirth (FOC), but this is not borne out in the literature. A systematic review of 24 papers presenting prevalence of FOC from Australia, Canada, the United States and nine European countries, found that around 6–15% of women suffer from severe fear of childbirth [10], which may sometimes lead to a request for elective caesarean birth. Stress, lack of social support, depression and anxiety are linked with fear during pregnancy, and the strongest predictor for fear in multiparous women is a previous operative birth or negative birth experience [11].

The International Federation of Gynaecology and Obstetrics (FIGO), in a recently published position paper, stated that *“Worldwide there is an alarming increase in caesarean section (CS) rates*,*”* and continued *“The large variation in CS rates indicates that these rates have virtually nothing to do with evidence-based medicine*.*”* They end by calling for the help of *“governmental bodies*, *UN partners*, *professional organisations*, *women’s groups*, *and other stakeholders to reduce unnecessary CSs*.*”* [12].

As part of a wider research initiative aimed at developing an evidence based intervention for reducing unnecessary caesareans safely, we conducted a systematic review of systematic reviews (overview) to identify and highlight antenatal and intrapartum interventions that have shown to be effective for reducing caesarean birth, promoting vaginal birth and/or reducing fear of childbirth. In reporting this review, we adhered, in as far as is possible, to the Preferred Reporting of Items for Systematic Reviews and Meta-Analyses (PRISMA) statement [13].

## Materials and methods

### Inclusion criteria

#### Types of participants

Populations of low- and high-risk pregnant women, of any gestation from pregnancy up to the birth of the baby and clinicians engaged in the care of women in pregnancy or childbirth were included.

#### Types of interventions

Any antenatal or intrapartum intervention that is designed to reduce caesarean birth, promote vaginal birth or reduce fear of childbirth or other antenatal or intrapartum interventions where mode of birth (caesarean or vaginal birth) or fear of childbirth are listed as outcomes of interest. The intervention can be applied at any time in pregnancy to the birth of the baby, but not postpartum and can be targeted towards women and/or clinicians.

#### Types of comparators

Comparator treatment included ‘usual care’ (as described by the authors of the included reviews), placebo or no intervention. As we wished to identify and describe interventions shown to be effective for reducing caesarean, rather than a hierarchy of comparative interventions of effect, reviews comparing antenatal or intrapartum interventions with alternative antenatal or intrapartum interventions were excluded, unless there was a control group receiving ‘usual care’.

#### Types of outcomes

To be included in the review, at least one of the following outcomes must have been reported; caesarean birth, vaginal birth (spontaneous or instrumental vaginal birth) or fear of childbirth.

#### Types of studies

Knowledge of the literature indicates that a number of systematic reviews of interventions designed to reduce caesarean birth have already been performed [14–17]. Furthermore, well conducted systematic reviews provide higher level evidence than individual randomised trials and other studies. For this reason, we included systematic reviews only, that evaluated antenatal and intrapartum interventions and reported their effect on at least one of our primary outcomes. National guidelines and Committee, Royal College or national Association (midwifery and obstetric) ‘Opinions’, Guidelines (e.g. RCOG Green-Top Guidelines) or Statements were also included if they provided recommendations for practices for reducing caesarean birth.

### Outcomes

Our primary outcomes of interest were caesarean birth, vaginal birth (spontaneous or instrumental assisted) and fear of childbirth. Our secondary outcomes were adverse effects (maternal and neonatal) associated with the intervention (short or long-term as defined by the review authors), satisfaction with the intervention (women and clinicians), adherence/compliance to the intervention, costs and evidence based recommendations for practices for reducing caesarean birth.

### Search and selection strategy

#### Database searching

To identify relevant reviews/reports, the following electronic databases were searched from January 2000 to January 2018: The Cochrane Database of Systematic Reviews (CDSR), PubMed, CINAHL and EMBASE. Although, traditionally, date restrictions are not applied to searching when conducting systematic reviews of primary research, we wished to identify and describe the most up-to-date contemporary evidence of effect for reducing caesarean birth, being cognisant of changes in approaches to clinical care over time, while acknowledging also that systematic reviews of healthcare interventions began to emerge in the 1990s. For this reason we fixed our starting search date to the year 2000. Language restrictions were not applied to the search. The selection of relevant papers for inclusion in the review, however, was restricted to English language publications. Searching all languages enabled us to identify the extent of potentially eligible additional papers that were not included and consider if their exclusion might be a source of language bias.

#### Searching other sources

The electronic database search was supplemented with a search for grey literature by searching PROSPERO (a database of registered systematic review protocols) and by screening the reference lists of included reviews for potentially relevant reviews that were not captured during the database search. The following websites were searched also to identify guidelines, opinions or statements on reducing caesarean birth: National Institute for Health and Clinical Excellence, Scottish Intercollegiate Guidelines Network, Guidelines International Network, and The World Health Organization. The websites of the following professional bodies were reviewed to seek information on the potential development or updating of guidelines for reducing caesarean birth: Institute of Obstetricians and Gynaecologists, Royal College of Physicians, Ireland, Royal College of Obstetricians and Gynaecologists (UK), American College of Obstetricians and Gynaecologists, Society of Obstetricians and Gynaecologists of Canada, Royal Australian and New Zealand College of Obstetricians and Gynaecologists, Royal College of Midwives (UK), Australian College of Midwives Australian, and New Zealand College of Midwives.

#### Search terms

Medical Subject Headings and keywords were used for searching for relevant literature. Table 1 presents the search terms used to guide the search, based on population, intervention, outcome and study/report design, combined within and across strings using the Boolean operands ‘OR’ and ‘AND’, respectively, with search terms adapted as appropriate for the various databases.

**Table 1:** Search terms.

*Population*	pregnant OR primigravida OR multigravida OR nulliparous OR obstetrician OR physician OR clinician OR doctor OR doctors OR midwife OR midwives OR “obstetric nurse” OR consultant OR Midwifery
*Intervention*	antenatal OR prenatal OR labour OR labor OR intrapartum
*Outcomes*	caesarean OR cesarean OR “caesarean section” OR “vaginal birth after caesarean” OR VBAC OR “next birth after caesarean” OR “trial of labor” OR “trial of labour” OR TOL OR TOLAC OR “spontaneous vaginal birth” OR ventouse OR forceps OR “instrumental birth” OR “fear of childbirth” OR tocophobia OR tokophobia
*Study design*	“systematic review” OR meta-analysis OR meta-synthesis OR “literature review” OR “clinical guideline” OR “practice guideline” OR “practice recommendation”

### Screening and selection for inclusion

Two reviewers (VS & LG) independently screened titles and abstracts, based on the review’s inclusion criteria. Full texts of all reports that were judged to be potentially relevant on title and abstract screening were retrieved and independently reviewed by two reviewers. Final inclusion was determined based on agreement of both reviewers. Any disagreement or uncertainty was resolved through discussion and consensus, or by reverting to a third reviewer.

### Assessment of methodological quality

Two pairs of two reviewers (VS & LG and CB & MC) independently assessed the methodological quality of the included reviews and guidelines. The AMSTAR-2 tool (A Measurement Tool to Assess Systematic Reviews (https://amstar.ca/docs/AMSTAR-2.pdf) was used to assess the methodological quality of the included systematic reviews [18]. Reviews judged to be of poor methodological quality, based on receiving a ‘critically low’ assessment (i.e. more than one critical flaw with and without non-critical weaknesses) were subsequently excluded as they *‘should not be relied on to provide an accurate and comprehensive summary of the available studies’* [18]. The AGREE II tool, an international tool for assessing the quality and reporting of practice guidelines (http://www.agreetrust.org/agree-ii/) was used to assess the quality of the included guidelines. We did not exclude guidelines on the basis of low AGREE II scores, rather we considered all recommendations, and drew, in the main, on recommendations underpinned by high level evidence (i.e. level 1 or level A evidence) for informing the results of our review and in highlighting clinical practices that reduce caesarean birth.

### Data extraction

Two pairs of two reviewers (VS & LG and CB & MC) independently extracted the data from the included reports. A data extraction form was pre-designed and piloted to ensure its appropriateness for extracting the relevant data. Data that were extracted from the reviews included aim of the review, year the review was published, population characteristics, description of the intervention and comparator, outcome data for both groups and the results (narrative or meta-analysed data, as available). Data extracted from the guidelines included country or region of origin, year the guideline was developed/published, guideline development strategy, key recommendations and guideline implementation strategy (including plans for auditing implementation).

### Data synthesis

Data synthesis is narrative due to heterogeneity in topics and reviews, supplemented by summary evidence tables where interventions demonstrating evidence of effect for our pre-specified primary and secondary outcomes is highlighted. We had initially planned to re-meta-analyse the data where two or more reviews reported on the same/similar intervention for outcomes relevant to our review and the reviews included different studies to each other; however due to the large numbers of reviews, and volume of individual studies in some of these, many of which were not accessible, this was not possible. Alternatively, for dichotomous outcomes, we narratively present the summary effect estimates provided in the review; that is, odds ratio (OR) or relative risk (RR), with 95% confidence intervals (CI). For continuous data, where possible, we narratively present the mean differences (MD) between the groups with 95% CI. Recommendations from guideline reports are summarised in an evidence table and compared and contrasted. Recommendations for practices for reducing caesarean section, based on high level (level 1 or level A) evidence are identified and highlighted.

## Results

### Search and selection

A total of 2196 citations across four databases and 27 citations from other sources were retrieved. Of these 2223 records 390 were identified as duplicates and excluded, leaving 1833 for title and abstract screening. Following title and abstract screening a further 1448 were excluded as they were either not relevant to the review or did not meet the inclusion criteria. This resulted in full-text screening of 385 records. Of these 216 were excluded for the following reasons; 82 included alternative controls and not ‘usual care’, 41 did not report our primary outcomes, 32 were not systematic review or guideline, 13 were an earlier version of a Cochrane review that had been replaced by a more recent version, 10 were non-English language publication, eight did not address all components of a systematic review, eight did not include an intervention that met our review’s eligibility criteria, five were duplicate publications, five focused on the postpartum period, four were guidelines that were withdrawn or earlier versions, four were published abstracts only, two were guideline recommendations not specific to caesarean birth, one did not include our population of interest and one was a protocol publication.

**Fig 1:**
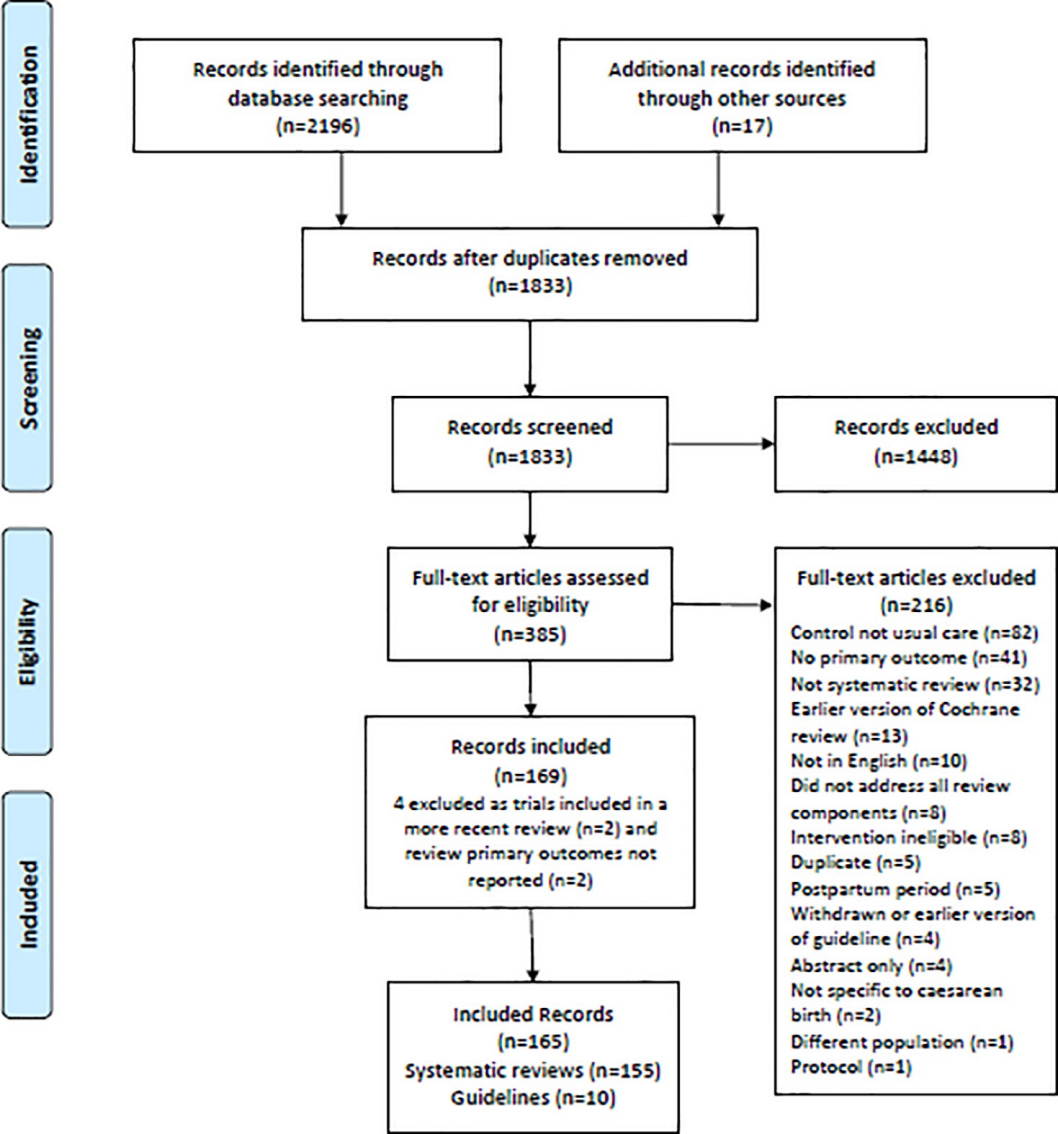
Search and selection flow diagram.

This resulted in 169 reports; 159 systematic reviews and 10 practice guidelines. On further review of the 159 systematic reviews, another four were subsequently excluded as two included trials all of which were included in a more recent review [19, 20], and two did not report on any of our pre-specified primary outcomes [21, 22]. This resulted in the inclusion of 155 systematic reviews. The search and selection process is illustrated in Fig 1. S1 File provides the list of references of these 155 reviews and 10 practice guidelines.

### Quality assessment

Of the 155 included systematic reviews, 66, 14 and 21, respectively, received a high, moderate and low rating on AMSTAR-2 assessment (see S1 Table for details). The remaining 54 reviews received a critically low rating on AMSTAR-2, and were subsequently excluded from the analyses (S2 Table). The AGREE II overall quality scores for the 10 included guidelines, based on four assessors (VS, LG, CB & MC) ranged from 28% to 86% (S3 Table).

### Characteristics of included reviews and guidelines

S1 Table provides the Summary Characteristics, including AMSTAR-2 ratings and the reported results, for our pre-specified outcomes that were reported in the 101 included systematic reviews. Twenty-five included reviews evaluated various methods (pharmacological, non-pharmacological or mechanical) for induction of labour (IOL), third trimester cervical ripening or augmentation of labour. Methods for fetal surveillance, for example, various modalities for fetal heart rate (FHR) monitoring, ultrasound in pregnancy, fetal movement counting, and biophysical profile, were the second most commonly evaluated interventions in the included reviews (14 reviews). Examples of other areas of clinical care commonly assessed in the reviews included interventions for preventing and treating gestational diabetes mellitus (GDM) (10 reviews), pharmacological, non-pharmacological, and alternative or complementary methods for pain relief in labour (9 reviews), interventions for preventing and treating preterm birth (6 reviews), oral supplements in pregnancy (6 reviews), and alternative programmes for antenatal care (6 reviews). Only one included review focused exclusively on fear of childbirth [23].

S3 Table presents a summary of the key practice recommendations related to caesarean birth from the 10 national/international practice guidelines. The publication dates for the 10 included guidelines ranged from 2009 to 2017. Seven guidelines were from the US, one from France, one from Canada and the remaining guideline was a FIGO international guideline. Four guidelines focused on vaginal birth after caesarean (VBAC), and one each focused on external cephalic version (ECV), safe prevention of primary caesarean birth, the Ten-Group Classification System for caesarean birth, breech vaginal birth, post-term pregnancy, and approaches to limit interventions during labour and birth.

### Results for primary outcome measures

#### Caesarean birth

Ninety-nine of the 101 included reviews reported on the outcome caesarean birth (S1 Table). The interventions evaluated in the 99 reviews were broadly varied and focused on multiple clinical topics (Table 2).

**Table 2:** Clinical topics across the included reviews that reported on caesarean birth.

*Clinical topic*	*No*. *of reviews*
Induction of labour (IOL), third trimester cervical ripening or augmentation of labour	25
Methods of fetal surveillance (e.g. FHR monitoring, ultrasound in pregnancy, fetal movement counting)	14
Prevention or treatment of gestational diabetes mellitus (GDM)	10
Pharmacological, non-pharmacological, and alternative or complementary methods for pain relief in labour (e.g. Epidural, TENS, hypnosis, acupressure)	9
Oral supplements (e.g. Vitamin C, Vitamin D, Zinc)	6
Preventing or treating preterm birth	6
Types of or alternative methods for delivering antenatal care	6
Antenatal or intrapartum support (e.g. continuous one-to-one care in labour, additional social support)	3
External cephalic version	3
Pre-eclampsia	3
Amnioinfusion	2
Information about caesarean birth	2
Antenatal pelvimetry	1
Birth settings	1
Fundal pressure in the second stage of labour	1
Immersion in water	1
Manual rotation for malposition	1
Non-clinical interventions for reducing unnecessary caesarean birth	1
Partogram	1
Pre-labour rupture of membranes at term	1
Weight gain during pregnancy	1

Of the 99 reviews, 25 reported a difference between the groups under investigation on caesarean birth; 17 reviews reported a reduced risk of caesarean with 25 interventions, and eight reviews reported an increased risk with nine interventions (Table 3 and Table 4, respectively, listed alphabetically by author).

**Table 3:** Interventions shown to reduce rates of caesarean birth.

*Author*, Year*	*Intervention and comparator*	*Caesarean birth (CB); RR/OR (95% CI)*, *number of trials; n = number of participants*
Alfirevic 2014	Oral misoprostol for IOL versus placebo/no treatment in pregnant women	RR 0.72 (0.54 to 0.95), 8 trials, n = 1029
Alfirevic 2016	All pharmacological (all routes and doses), mechanical and complementary methods used for IOL versus placebo/no intervention in pregnant women carrying a viable fetus and who are eligible for any method of third-trimester cervical ripening or IOL	586 trials, n = 96771 - Vaginal PGE2 (gel) OR 0.79 (0.65 to 0.94) - Intracervical PGE2 OR 0.83 (0.69 to 0.98) - Vaginal misoprostol tablet < 50μg OR 0.70 (0.57 to 0.85) - Vaginal misoprostol tablet ≥ 50μg OR 0.73 (0.59 to 0.88) - Oral misoprostol tablet ≥ 50μg OR 0.72 (0.58 to 0.88) - Titrated (low-dose) oral misoprostol solution OR 0.62 (0.47 to 0.80)Foley catheter OR 0.76 (0.61 to 0.95) - Membrane sweeping OR 0.74 (0.53 to 0.99) - Buccal/sublingual misoprostol OR 0.68 (0.51 to 0.89)
Bohren 2017	Continuous, one-to-one intrapartum support versus usual care in any birth setting	RR 0.75 (0.64 to 0.88), 24 trials, n = 15347
Cluver 2015	Interventions to facilitate ECV at term versus placebo/no intervention	Beta stimulants RR 0.77 (0.67 to 0.88), 6 trials, n = 742; Tocolytics in nulliparous RR 0.85 (0.75 to 0.97), 2 trials, n = 170
Dodd 2017	Progestogen by any route (IV, IM, oral or vaginal) for the prevention of preterm birth versus placebo or no treatment	Vaginal progesterone RR 0.93 (0.88 to 0.98), 6 trials, n = 2143
East 2014	Use of fetal pulse oximetry (FPO) with or without concurrent use of conventional fetal monitoring versus conventional only (no pulse oximetry) in women in labour where fetal monitoring is clinically indicated	FPO and CTG vs CTG only from 36 weeks; FBS prior to study entry RR 0.44 (0.24 to 0.81), 1 trial, n = 146
Gulmezoglu 2012	Labour induction versus expectant management in pregnant women at or beyond term at low risk for complications	All women RR 0.89 (0.81 to 0.97), 21 trials, n = 8749; 41 weeks RR 0.74 (0.58 to 0.96), 4 trials, n = 998; Cervix unfavourable RR 0.88 (0.80 to 0.98), 8 trials, n = 5051
Hapangama 2009	Mifepristone for third trimester cervical ripening or IOL versus placebo/no treatment in pregnant women due for third trimester IOL carrying a viable fetus	Mifepristone all doses RR 0.74 (0.60 to 0.92), 9 trials, n = 1043
Hodnett 2010	Standardized or individualized programs of additional social support versus usual care for pregnant women believed at high risk for giving birth to babies that are either preterm or weigh less than 2500 gm, or both, at birth	RR 0.87 (0.78 to 0.97), 9 trials, n = 4522
Hofmeyr 2012b	Amnioinfusion (AI) versus no AI in women whose babies were considered to be at increased risk of, or had FHR patterns suggestive of, umbilical cord compression in labour	Transcervical AI RR 0.62 (0.46 to 0.83), 13 trials, n = 1493; CS for suspected fetal distress RR 0.46 (0.31to 0.68), 12 trials, n = 1588
Hofmeyr 2014	Amnioinfusion for meconium-stained liquor in labour versus no amnioinfusion in women in labour with moderate or thick meconium staining of the amniotic fluid	RR 0.59 (0.41 to 0.84), 3 trials, n = 1137 (limited peripartum surveillance)
Hofmeyr 2015	ECV versus no ECV in pregnant women with babies in the breech presentation at or near term	RR 0.57 (0.40 to 0.82), 8 trials, n = 1305
Kavanagh 2006	Hyaluronidase versus placebo/no treatment for third trimester cervical ripening or IOL in pregnant women due for third trimester induction of labour, carrying a viable fetus	All women RR 0.37 (0.22 to 0.61), 1 trial, n = 168; Primiparae RR 0.43 (0.23 to 0.81), 1 trial, n = 94; Multiparae RR 0.28 (0.12 to 0.67), 1 trial, n = 74; Women with previous caesarean RR 0.35 (0.15 to 0.81), 1 trial, n = 29
Lavender 2013	Labour management using a partogram versus no partogram in women with singleton pregnancies, cephalic in spontaneous labour at term	Low-resource setting RR 0.38 (0.24 to 0.61), 1 trial, n = 434
Smith 2006	Complementary and alternative therapies used in labour (but not biofeedback) with or without concurrent use of pharmacological or non-pharmacological interventions versus placebo or no treatment in women in spontaneous or induced labour, for pain management in labour	Hypnosis RR 0.46 (0.30 to 0.72), 1 trial, n = 520
Smith 2011a	Acupuncture or acupressure versus placebo, no treatment for pain management in labour	Acupressure RR 0.24 (0.11 to 0.54), 1 trial, n = 120
Wei 2013	Early augmentation with amniotomy and oxytocin versus conservative form of management in pregnant women in spontaneous labour	Prevention studies RR 0.87 (0.77 to 0.99), 11 trials, n = 7753

*See S1 File for full references to the reviews

**Table 4:** Interventions shown to increase rates of caesarean birth.

*Author*, Year*	*Intervention and comparator*	*Caesarean birth (CB); RR/OR (95% CI)*, *number of trials; n = number of participants*
Alfirevic 2009	Intravenous oxytocin alone for third trimester cervical ripening or IOL versus placebo /expectant management in pregnant women	RR 1.17 (1.01 to 1.35), 24 trials, n = 6620
Alfirevic 2017	Continuous CTG during labour versus no fetal monitoring or IA with Pinard stethoscope or hand-held Doppler ultrasound device	CTG versus IA: All women RR 1.63 (1.29 to 1.33), 11 trials, n = 18861; High-risk women RR 1.91 (1.39 to 2.61), 6 trials, n = 2069; Low-risk women RR 2.06 (1.24 to 3.45), 2 trials, n = 1431
Bond 2017	Planned early birth (IOL or CS) versus expectant management for women with preterm pre-labour rupture of the membranes between 24 and 37 weeks’ gestation	RR 1.26 (1.11 to 1.44), 12 trials, n = 3620
Dodd 2015	Antenatal care specifically designed for women with a multiple pregnancy versus usual care	RR 1.38 (1.06 to 1.81), 1 trial, n = 162
Martis 2017	IA during labour versus another method of IA in pregnant women	Intermittent CTG versus Pinard - Caesarean for fetal distress RR 2.92 (1.78 to 4.80), 1 trial, n = 633 - Overall RR 1.92 (1.39 to 2.64), 1 trial, n = 633 - Doppler versus Pinard - Caesarean for fetal distress RR 2.71 (1.64 to 4.48), 1 study, n = 627
Pattinson 2017	Pelvimetry versus no pelvimetry in pregnant women with a singleton, cephalic presentation fetus who have or have not had a previous caesarean section	All women RR 1.34 (1.19 to 1.52), 5 trials, n = 1159; Women with no previous caesarean RR 1.24 (1.02 to 1.52), 3 trials, n = 769; Women with previous caesarean RR 1.45 (1.26 to 1.67), 2 trials, n = 390
Stock 2016	Immediate delivery versus deferred delivery for a set period of time, until test results worsen, or expectant management in pregnant women at > 36 weeks’ in whom there is clinical suspicion of fetal compromise	RR 1.15 (1.07 to 1.24), 1 trial, n = 547
Till 2015	Direct incentives explicitly linked to initiation and frequency of prenatal care (e.g. cash, vouchers, coupons or products not generally offered to patients as a standard of prenatal care) versus no incentives to increase utilization of timely prenatal care among pregnant women	RR 1.97 (1.18 to 3.30), 1 study, n = 979

*See S1 File for full references to the reviews

Interventions shown to reduce caesarean birth (Table 3) were varied methods of IOL (e.g. vaginal and intracervical PGE2, Foley catheter, oral misoprostol, mifepristone), continuous, one-to-one intrapartum support, external cephalic version (ECV) with/without beta-stimulants and tocolytics, vaginal progestogen for preventing preterm birth, fetal pulse oximetry and cardiotocography (CTG) from 36 weeks’ gestation, standardised or individualised programmes of additional social support for pregnant women believed at high risk of giving birth to babies that are either preterm or weigh less than 2500gm, transcervical amnioinfusion in women whose babies were considered to be at increased risk of, or had FHR patterns suggestive of, umbilical cord compression in labour, amnioinfusion for meconium-stained liquor in women receiving limited peripartum surveillance, partogram use in low resource setting, hypnosis and acupressure for pain management during labour, and early augmentation with amniotomy and oxytocin.

Interventions shown to increase caesarean birth (Table 4) were intravenous oxytocin alone for third trimester cervical ripening or IOL, continuous CTG during labour, planned early birth in women with preterm pre-labour rupture of the membranes between 24 and 37 weeks’ gestation, antenatal care specifically designed for women with a multiple pregnancy, IA with Doppler or intermittent CTG compared to Pinard, antenatal pelvimetry, immediate delivery in pregnant women at > 36 weeks’ gestation in whom there is clinical suspicion of fetal compromise, and direct incentives explicitly linked to initiation and frequency of prenatal care.

#### Vaginal birth (spontaneous or instrumental assisted)

Fifty-seven reviews reported on the outcome vaginal birth, described variously in the reviews as spontaneous vaginal birth, normal vaginal birth, vaginal birth after previous caesarean section (VBAC), vaginal breech birth, instrumental vaginal birth, operative vaginal birth, assisted vaginal birth and vacuum extraction (S1 Table).

Interventions found to reduce the risk of instrumental vaginal birth were:

- Vaginal PGE2 pessary (slow release) for pregnant women who are eligible for any method of third-trimester cervical ripening or IOL OR 0.72 (0.50 to 0.99);
- Foley catheter for pregnant women who are eligible for any method of third-trimester cervical ripening or IOL OR 0.68 (0.50 to 0.91);
- Continuous, one-to-one intrapartum support RR 0.90 (0.85 to 0.96), 19 trials, n = 14118;
- Mifepristone for third trimester IOL RR 1.43 (1.04 to 1.96), 7 trials, n = 814);
- Alternative institutional birth setting compared to conventional setting for pregnant women at low risk of obstetric complications RR 0.89 (0.79 to 0.99), 8 trials, n = 11202
- Amnioinfusion for women in labour with moderate or thick meconium staining of the amniotic fluid RR 0.68 (0.50to 0.91), 9 trials, n = 2059
- Any type of fetal ECG waveform analysis, alone or in combination with another method for pregnant women with a perceived need for continuous EFM RR 0.92 (0.86 to 0.99), 6 trials, n = 26446
- Vaginal prostaglandins F2a for pregnant women due for third trimester IOL, carrying a viable fetus RR 0.63 (0.47, 0.84), 3 trials, n = 435.

Interventions found to increase the risk of instrumental/assisted vaginal birth were:

- Continuous CTG during labour in low-risk women RR 1.15 (1.01 to 1.33), 10 trials, n = 18615
- Epidural for pain relief in labour RR 1.42 (1.28 to 1.57), 23 trials, n = 7935.

Interventions found to increase spontaneous vaginal birth were:

- Immersion in water in first stage RR 1.26 (1.09 to 1.45), 1 trial, n = 106
- Alternative institutional birth setting compared to conventional setting for pregnant women at low risk of obstetric complications RR 1.03 (1.01, 1.05), 8 trials, n = 11202
- Opinion leaders (VBAC) RR 1.74 (1.45 to 2.09), 1 study, n = 1972;
- Hypnosis for pain relief in labour RR 1.32 (1.19 to 1.46), 3 studies, n = 645.

Interventions found to reduce spontaneous vaginal birth were:

- Continuous CTG during labour for low-risk women RR 0.91 (0.86 to 0.96), 11 trials, n = 18861
- Planned early birth in women with preterm pre-labour rupture of membranes (PPROM) before 37 weeks RR 0.94 (0.91 to 0.97), 12 trials, n = 3618;
- X-ray pelvimetry as an assessment for suitability for VBAC RR 0.38 (0.25 to 0.58), 1 study, n = 288.

#### Fear of childbirth

Three reviews only reported on the outcome fear of childbirth [15, 23–24], of which one focused exclusively on planned interventions for pregnant women with tokophobia who have requested a caesarean [23]. The interventions assessed in the reviews were multiple and included education, audit and feedback, psychological support, quality improvement strategies, financial incentives for procedures, mandatory second opinions, and hypnosis with and without concurrent use of other pharmacological or non-pharmacological methods of pain relief.

The interventions shown to be effective for reducing fear of childbirth were intensive group therapy for reducing fear of pain in labour (p = 0.041) and fear of obstetrician’s unfriendly behaviour (p = 0.054) [15], use of hypnosis with or without other methods of pain relief (Wijma score at 6 weeks postpartum MD -4.60 [-8.34 to -0.86], 1 trial, 678 women) [24], and intensive therapy compared with conventional therapy for reducing birth related concern (p = 0.022; 1 study, n = 176) [23].

### Secondary outcome measures

#### Adverse effects associated with the intervention

Sixty-five of the 101 included reviews reported on the outcome of adverse effects (S1 Table). These reviews centred on the following antenatal or intrapartum clinical topics; IOL or third trimester cervical ripening (14 reviews), methods of fetal surveillance (13 reviews), prevention and treatment of GDM (8 reviews), interventions for preventing or treating preterm birth (6 reviews), supplements in pregnancy (4 reviews), antenatal care (e.g. alternative pathways) (4 reviews), VBAC/information regarding caesarean birth (3 reviews), ECV (3 reviews), support in labour (2 reviews), and one review each on immersion in water, twin pregnancy, birth setting, fundal pressure in second stage of labour, amnioinfusion, amniotomy, manual rotation for malposition, and management of pain in labour.

Perinatal death was the most commonly reported adverse outcome, reported in 54 reviews, followed by stillbirth (22 reviews), and neonatal death (21 reviews). The remaining reported adverse effects were maternal death (15 reviews), and serious maternal morbidity (e.g. ICU admission, infection, cardiac arrest, uterine rupture) (12 reviews).

Of the 65 reviews, six demonstrated evidence of adverse effects (Table 5). These were an increased risk of neonatal death with planned early birth compared to expectant management in women with PPROM [25], an increased risk of perinatal death with a programme of reduced antenatal visits compared to standard care [26], and an increased risk of maternal side effects with mifepristone compared to placebo, although these were mainly minor gastro-intestinal upsets (nausea, diarrhoea and vomiting) [27]. Contrastingly, the risk of perinatal death was reduced with computerised CTG compared to traditional CTG [28], with IOL at term or post-term compared with expectant management in pregnant women at low risk for complications [29], and when amnioinfusion was used during labour in women with moderate or thick meconium staining of the amniotic fluid [30].

**Table 5:** Interventions shown to affect adverse effects positively or negatively.

*Author*, *Year*	*Intervention and comparator*	*Adverse effect; RR/OR (95% CI)*, *number of trials; n = number of participants*
Bond 2017	Planned early birth (IOL or CS) versus expectant management for women with PPROM 24 and 37 weeks’ gestation	Neonatal death RR 2.55 (1.17 to 5.56), 11 trials, n = 3316
Dowswell 2015	Antenatal care programmes with reduced visits for low-risk women with standard/usual care	Perinatal death RR 1.15 (1.01 to 1.32), 3 cluster trials
Grivell 2015	Antenatal CTG (both traditional and computerised assessments) in improving outcomes for pregnant women and their babies	Perinatal death RR 0.20 (0.04 to 0.88) 2 trials, n = 469
Gulmezoglu 2012	Policy of IOL at term or post-term compared with awaiting spontaneous labour or later IOL in pregnant women at or beyond term at low risk for complications	Perinatal death RR 0.31 (0.12 to 0.81), 17 trials, n = 7407
Hapangama 2009	Mifepristone for third trimester cervical ripening or IOL versus placebo/no treatment in pregnant women due for third trimester IOL carrying a viable fetus	Maternal adverse effects RR 1.51 (1.06 to 2.15), 4 trials, n = 734
Hofmeyr 2014	Amnioinfusion for meconium-stained liquor versus no amnioinfusion in women in labour with moderate or thick meconium staining of the amniotic fluid	Perinatal death RR 0.35 (0.18 to 0.66), 10 trials, n = 3913

#### Satisfaction with the intervention (women and clinicians)

Twenty-nine of the 101 reviews reported on the outcome of satisfaction (S1 Table). Clinical topics assessed in these reviews were methods of IOL and/or third trimester cervical ripening (7 reviews), pharmacological (e.g. epidural) or non-pharmacological/alternative methods for pain relief/management during labour (9 reviews), additional forms of social or decisional aid support programmes (4 reviews), specialised antenatal care (e.g. group antenatal care) compared to usual antenatal care (3 reviews), methods of fetal surveillance (2 reviews), and one review on each of the following; continuous one-to-one intrapartum support, alternative birth settings, active management of labour package, interventions to facilitate ECV, and immersion in water. Table 6 presents the interventions shown to affect satisfaction with the intervention.

**Table 6:** Interventions shown to affect satisfaction positively or negatively.

*Author* *Year*	*Intervention and comparator*	*Satisfaction; RR/OR (95% CI)*, *number of trials; n = number of participants*
Alfirevic 2009	Intravenous oxytocin alone versus placebo/expectant management for third trimester cervical ripening and IOL	Women were less likely to be dissatisfied with IOL compared with expectant management; 5.9% versus 13.7%, RR 0.43 (0.33 to 0.56), 1 trial, n = 5041
Bohren 2017	Continuous, one-to-one intrapartum support versus usual care	Negative feelings about birth experience RR 0.69 (0.59 to 0.79), 11 studies, n = 11133
Cluver 2015	Interventions to facilitate ECV versus placebo	Systemic opioids RR 2.60 (1.25 to 3.95), 1 trial, n = 60
Dowswell 2015	Provision of a schedule of reduced number of visits, with or without goal-oriented antenatal care versus usual care in low-risk pregnant women attending ANC	Satisfied with quality of prenatal care MD -0.20 (-0.28 to -0.11), 2 trials, n = 2198, and would choose same schedule in future (yes) RR 1.12 (1.05 to 1.20), 1 trial, n = 1862
Dowswell 2009a	TENS (any model or type) versus placebo TENS or routine care on pain in labour	TENS to acupoints RR 4.1 (1.81 to 9.29), 1 trial, n = 90
Dowswell 2009b	Antenatal day care: admission and discharge home with no overnight stay *versus* Inpatient care or routine management (which includes the option of inpatient care)	“I am satisfied with the care I received” (number disagreeing or not sure) RR 0.40 (0.18 to 0.88), 1 trial, n = 350
Hodnett 2012	Alternative institutional birth setting *versus* conventional hospital setting	Very positive views of care RR 1.96 (1.78 to 2.15), 2 trials, n = 1207
Kobayashi 2017	Assessment programmes in early labour versus no intervention or usual care for low risk women during early labour	MD 16.00 (7.53 to 24.47), 1 study, n = 201
Khunpradit 2011b	Directed interventions versus usual care non-clinical interventions for reducing unnecessary caesarean section rates.	Decision analysis Adj diff 0.14 (0.02 to 0.27) P = 0.022
Smith 2011c	Relaxation techniques versus placebo/no treatment/usual care for pain management in labour	RR 8.0 (1.10 to 58.19), 1 trial, n = 40
Whitworth 2015	Routine US versus selective US Women with early pregnancies, i.e. less than 24 weeks’ gestation	Mother not satisfied with care RR 0.80 (0.65 to 0.99), 1 trial, n = 634

#### Adherence/Compliance to the intervention

Only one of the 101 reviews reported on adherence to the intervention. Han *et al*. [31] evaluated any physical exercise for pregnant women with pre-existing type 1 or type 2 diabetes for preventing gestational diabetes mellitus compared to usual care (5 studies, 1115 women). The results found ‘Excellent’ adherence to the intervention in four of the five included trials.

#### Costs

Four reviews reported on the outcome of cost [24, 32–34]. Alfirevic’s network meta-analysis [32], which evaluated all pharmacological (all routes and doses), mechanical and complementary methods used for IOL found that all methods of induction had lower expected total costs than placebo because they reduce costly outcomes such as vaginal birth after 24 hours, caesarean birth and admission to neonatal intensive care unit. Vogel, however, who assessed pharmacological and mechanical interventions for IOL in outpatient settings found no difference in the total cost of care package (GBP) with vaginal isosorbide mononitrate compared with placebo (MD 11.98, 95% CI -105.34 to 129. 30, 1 study, 350 women) [34]. No differences in costs were found when antenatal day care units (admission and discharge home with no overnight stay) were compared with hospital admission for women with complicated pregnancy (average total cost Australian $; MD 415.10, 95% CI -603.86, 1434.06, 1 trial, n = 395 women) [33], or when hypnosis compared to standard care was used for pain management during labour [24].

#### Evidence based recommendations for reducing CS

The levels of evidence provided by the 10 included guidelines ranged from professional consensus through to level I/level A evidence (S3 Table). Recommendations based on the highest level evidence include offering ECV to women who are near term with breech presentations if there are no contraindications, counselling and offering most women with one previous caesarean with a low-transverse incision a planned VBAC, not using misoprostol for cervical ripening or IOL in women at term who have had a caesarean or major uterine surgery, monitoring FHR decelerations as this may safely reduce the rate of caesarean, and performing (or offering) IOL at, or beyond, 41 weeks to reduce the risk of caesarean and the risk of perinatal morbidity and mortality.

## Discussion

A systematic review led by the World Health Organization (WHO) showed that, when caesarean section rates rose above 9–16% at population level, they did not appear to result in any decrease in maternal or neonatal mortality [7]. Given the increased levels of maternal and neonatal morbidity linked to caesarean section, described above, it is imperative now that all clinicians actively work to decrease high caesarean section rates. Our overview has identified twenty-five interventions of varied topics and practices that can be considered for implementing in practice in an effort to halt, or reverse, the rise in individual maternity unit, regional or national caesarean birth rates. These interventions should be considered in the context of birth settings, resources and practices already implemented. All interventions were found to be as safe as the ‘usual care’ given in control groups, which provides reassurance to women and clinicians that they can be implemented to reduce caesarean section rates safely.

One of the strengths of our review was that we restricted our search date to systematic reviews published from 2000 only, as these would include more modern trials based on clinical practices that are still being used. A further strength of our review is that the evidence of effect for our pre-specified outcomes is from reviews that were mostly of high or moderate quality (80% of the included reviews) indicating that they provide/may provide an accurate summary of the results of their included studies [18]. Consideration should be given, however, to the possibility that additional trials conducted since the search date of the included reviews, if added to the reviews, could potentially alter their findings. In this sense, interventions highlighted as currently being effective, may no longer be comparatively effective as additional data are added, and some that may appear to be ineffective now may become so as new trial results are added. This is particularly so for interventions where the evidence of effect is based on single (or very few) studies with few participants, and clinical decision-makers may wish to factor this in when/if implementing the results of this overview. One substantive review and one international guideline that assessed non-clinical interventions for reducing unnecessary caesarean section have been published since our search date [35. 36]. The first of these is an update of an included Cochrane review [15]. Additional evidence from this review to that already provided in our review of reviews demonstrated that childbirth training workshops for mothers alone (RR 0.55, 95% CI 0.33 to 0.89) and for couples (RR 0.59, 95% CI 0.37 to 0.94) may reduce caesarean sections [35]. The guideline, published by the World Health Organisation, and drawing largely on the evidence from the updated Cochrane review, presents a series of recommendations related to interventions targeted at women, healthcare professionals, and health organisations, facilities or systems [36].

## Conclusion

This overview of reviews and practice based recommendations identifies and highlights antenatal and intrapartum interventions and practices that have been shown to be effective in reducing caesarean birth, promoting vaginal birth and reducing fear of childbirth in low and high-risk maternity populations. These interventions and practices which may be feasible or applicable to all countries and settings or to some only, include various methods of IOL, continuous one-to-one intrapartum support, ECV, standardised or individualised programmes of additional social support for high risk pregnant women, partogram use in low resource setting, hypnosis and acupressure for pain management during labour, alternative institutional birth setting for low risk women, and intensive group therapy. The overview, in bringing all of the contemporary evidence together in one place, provides a valuable, extensive resource for clinicians to consider within the context of their healthcare settings, when making decisions on practices that should be implemented to reduce unnecessary caesarean births safely.

## Supporting information

S1 File Complete reference list of the 155 systematic reviews and 10 practice guidelines. (DOCX)

S1 Table Summary characteristics of the 101 included systematic reviews based on AMSTAR-2 ratings of high, moderate or low. (DOCX)

S2 Table Summary characteristics of the 54 excluded reviews based on an AMSTAR-2 ‘critically low’ rating. (DOCX)

S3 Table Summary characteristics of the 10 included practice guidelines. (DOCX)

## References

[1] American College of Obstetricians and Gynecologists (ACOG). Safe prevention of the primary cesarean delivery. Obstetric Care Consensus No. 1. American College of Obstetricians and Gynecologists. Obstet Gynecol 2014; 123: 693–711. 10.1097/01.AOG.0000444441.04111.1d 24553167

[2] Liu S, Liston RM, Joseph KS, Heaman M, Sauve R, Kramer MS. Maternal mortality and severe morbidity associated with low-risk planned cesarean delivery versus planned vaginal delivery at term. Can Med Assoc J. 2007; 4: 455–460. 10.1503/cmaj.060870 PMC1800583 17296957

[3] van den Berg A, van Elburg R, van Geijn HP, Fetter WPF. Neonatal respiratory morbidity following elective caesarean section in term infants. A 5-year retrospective study and a review of the literature. EJOG Reprod Biol. 2001; 98: 9–13. 10.1016/s0301-2115(01)00292-5 11516792

[4] Lobel M, Deluca RS. Psychological sequelae of caesarean delivery: review and analysis of their causes and implications. Soc Sci Med. 2007; 64: 2272–84. 10.1016/j.socscimed.2007.02.028 17395349

[5] Kenny C, Devane D, Normand C, Clarke M, Howard A, Begley C. A cost-comparison of midwife-led compared with consultant-led maternity care in Ireland (the MidU study). Midwifery. 2015; 31:1032–1038 10.1016/j.midw.2015.06.012 26381076

[6] EURO-PERISTAT Project with SCPE and EUROCAT. European Perinatal Health Report. Nov 2018. Retrieved from www.europeristat.com, March 2019.

[7] Betran AP, Torloni MR, Zhang J, Ye J, Mikolajczyk R, Deneux-Tharaux C, et al What is the optimal rate of caesarean section at population level? Reproductive Health, 2015; 12: 57 10.1186/s12978-015-0043-6 26093498 PMC4496821

[8] Panda S, Begley C, Daly D. Clinicians' views of factors influencing decision-making for caesarean section: a systematic review and metasynthesis of qualitative, quantitative and mixed methods studies. PLoS ONE. 2018a 13:7: e0200941. 30052666 10.1371/journal.pone.0200941 PMC6063415

[9] Panda S, Begley C, Daly D, Karlström A, Larson B, Back L, Hildingsson I. Factors influencing decision-making for caesarean section in Sweden—a qualitative study. BMC Pregnancy and Childbirth. 2018b; 18(1): 377 10.1186/s12884-018-2007-7 30223780 PMC6142337

[10] Nilsson C, Hessman E, Sjöblom H, Dencker A, Jangsten E, Mollberg M, et al Definitions, measurements and prevalence of Fear of Childbirth: A systematic review. BMC Pregnancy and Childbirth. 2018, 18(1): 28 10.1186/s12884-018-1659-7 29329526 PMC5766978

[11] Dencker A, Nilsson C, Begley C, Jangsten E, Mollberg M, Patel H, et al Causes and outcomes in studies of fear of childbirth: a systematic review. Women and Birth. 2018, pii: S1871– 10.1016/j.wombi.2018.07.004 30115515

[12] Visser GHA, Ayres-de-Campos D, Barnea ER, de Bernis L, Di Renzo GC, Vidarte MFE, et al FIGO position paper: how to stop the caesarean section epidemic. The Lancet. 2018, 382: 10155, 1286–1287. 10.1016/S0140-6736(18)32113-5 30322563

[13] Liberati A, Altman DG, Tetzlaff J, Mulrow C, Gøtzsche PC, Ioannidis JP, et al The PRISMA statement for reporting systematic reviews and meta-analyses of studies that evaluate healthcare interventions: explanation and elaboration. BMJ. 2009; 21: 339. 10.1136/bmj.b2700 PMC2714672 19622552

[14] Horey D, Weaver J, Russell H. Information for pregnant women about caesarean birth. Cochrane Database Syst Rev. 2004, 1:CD003858. 10.1002/14651858.CD003858.pub2 PMC8745610 14974041

[15] Khunpradit S, Tavender E, Lumbiganon P, Laopaiboon M, Wasiak J, Gruen RL. Non-clinical interventions for reducing unnecessary caesarean section. Cochrane Database Syst Rev. 2011, 6:CD005528 10.1002/14651858.CD005528.pub2 21678348

[16] Lundgren I, Smith V, Nilsson C, Vehvilainen-Julkunen K, Nicoletti J, Devane D, et al Clinician-centred interventions to increase vaginal birth after caesarean section (VBAC): a systematic review. BMC Pregnancy Childbirth. 2015; 15:16 10.1186/s12884-015-0441-3 25652550 PMC4324420

[17] Nilsson C, Lundgren I, Smith V, Vehvilainen-Julkunen K, Nicoletti J, Devane D, et al Women-centred interventions to increase vaginal birth after caesarean section (VBAC): A systematic review. Midwifery. 2015, 31(7): 657–63. 10.1016/j.midw.2015.04.003 25931275

[18] Shea BJ, Reeves BC, Wells G, Thuku M, Hamel C, Moran J, et al AMSTAR 2: a critical appraisal tool for systematic reviews that include randomised or non-randomised studies of healthcare interventions, or both. BMJ. 2017; 358:j4008 10.1136/bmj.j4008 28935701 PMC5833365

[19] Gourounti K, Sandall J. Admission cardiotocography versus intermittent auscultation of fetal heart rate: effects on neonatal Apgar score, on the rate of caesarean sections and on the rate of instrumental delivery—a systematic review. Int J Nurs Stand. 2007; 44(6), 1029–35. 10.1016/j.ijnurstu.2006.06.002 16919279

[20] Catling-Paull C, Johnston R, Ryan C, Foureur MJ, Homer CS. Non-clinical interventions that increase the uptake and success of vaginal birth after caesarean section: a systematic review. J Adv Nurs. 2011l 67(8):1662–76. 10.1111/j.1365-2648.2011.05662.x 21535091

[21] Van den Berg I, Bosch JL, Jacobs B, Bouman I, Duvekot JJ, Hunik MG. Effectiveness of acupuncture-type interventions versus expectant management to correct breech presentation: a systematic review. Complement Ther Med. 2008; 16(2), 92–100. 10.1016/j.ctim.2008.01.001 18514911

[22] Wilcox CB, Nassar N, Roberts CL. Effectiveness of nifedipine tocolysis to facilitate external cephalic version: a systematic review. BJOG. 2011; 118(4): 423–8. 10.1111/j.1471-0528.2010.02824.x 21199292

[23] Weaver J, Browne J, Aras-Payne A, Magill-Cuerden J. A comprehensive systematic review of the impact of planned interventions offered to pregnant women who have requested a caesarean section as a result of tokophobia (fear of childbirth). JBI Libr Syst Rev. 2012; 10(28 Suppl): 1–20. 10.11124/jbisrir-2012-322 27820402

[24] Madden K, Middleton P, Cyna AM, Matthewson M, Jones L. Hypnosis for painmanagement during labour and childbirth. Cochrane Database Syst Rev 2016, Issue 5 Art. No.: CD009356 10.1002/14651858.CD009356.pub3 27192949 PMC7120324

[25] Bond DM, Middleton P, Levett KM, van der HamDP, Crowther CA, Buchanan SL, et al Planned early birth versus expectant management for women with preterm prelabour rupture of membranes prior to 37 weeks’ gestation for improving pregnancy outcome. Cochrane Database Syst Rev 2017, Issue 3 Art. No.: CD004735. 28257562 10.1002/14651858.CD004735.pub4 PMC6464692

[26] Dowswell T, Carroli G, Duley L, Gates S, Gülmezoglu AM, Khan-Neelofur D, et al Alternative versus standard packages of antenatal care for low-risk pregnancy. Cochrane Database Syst Rev 2015, Issue 7 Art. No.: CD000934 10.1002/14651858.CD000934.pub3 26184394 PMC7061257

[27] Hapangama D, Neilson JP. Mifepristone for induction of labour. Cochrane Database Syst Rev 2009, Issue 3 Art. No.: CD002865 10.1002/14651858.CD002865.pub2 19588336 PMC3992376

[28] Grivell RM, Alfirevic Z, Gyte GML, Devane D. Antenatal cardiotocography for fetal assessment. Cochrane Database Syst Rev 2015, Issue 9 Art. No.: CD007863 10.1002/14651858.CD007863.pub4 26363287 PMC6510058

[29] Gülmezoglu AM, Crowther CA, Middleton P, Heatley E. Induction of labour for improving birth outcomes for women at or beyond term. Cochrane Database Syst Rev 2012, Issue 6 Art. No.: CD004945 10.1002/14651858.CD004945.pub3 22696345 PMC4065650

[30] Hofmeyr GJ, Xu H, Eke AC. Amnioinfusion for meconium-stained liquor in labour. Cochrane Database Syst Rev 2014, Issue 1 Art. No.: CD000014 10.1002/14651858.CD000014.pub4 24453049 PMC7263444

[31] Han S, Middleton P, Crowther CA. Exercise for pregnant women for preventing gestational diabetes mellitus. Cochrane Database Syst Rev 2012, Issue 7 Art. No.: CD009021 10.1002/14651858.CD009021.pub2 22786521 PMC11534277

[32] Alfirevic Z, Keeney E, Dowswell T, Welton NJ, Medley N, Dias S, et al Which method is best for the induction of labour? A systematic review, network meta-analysis and cost-effectiveness analysis. Health Technol Assess. 2016; 20(65). 10.3310/hta20650 27587290 PMC5027380

[33] Dowswell T, Middleton P, Weeks A. Antenatal day care units versus hospital admission for women with complicated pregnancy. Cochrane Database Syst Rev 2009, Issue 4 Art. No.: CD001803 10.1002/14651858.CD001803.pub2 19821282 PMC4171387

[34] Vogel JP, Osoti AO, Kelly AJ, Livio S, Norman JE, Alfirevic Z. Pharmacological and mechanical interventions for labour induction in outpatient settings. Cochrane Database Syst Rev 2017, Issue 9 Art. No.: CD007701. 28901007 10.1002/14651858.CD007701.pub3 PMC6483740

[35] Chen I, Opiyo N, Tavender E, Mortazhejri S, Rader T, Petkovic J, et al Non-clinical interventions for reducing unnecessary caesarean section. Cochrane Database Syst Rev 2018, Issue 9 Art. No.: CD005528. 30264405 10.1002/14651858.CD005528.pub3 PMC6513634

[36] World Health Organisation. Recommendations non-clinical interventions to reduce unnecessary caesarean sections. Geneva: World Health Organization; 2018. Licence: CC BY-NC-SA 3.0 IGO.

