# Supplementary material for: Antenatal and intrapartum interventions for reducing caesarean section, promoting vaginal birth, and reducing fear of childbirth: An overview of systematic reviews
Source: PLoS One. 2019 Oct 24;14(10):e0224313. doi: 10.1371/journal.pone.0224313 (PMC6812784; doi:10.1371/journal.pone.0224313)
Supplement: S1 File — (DOCX) [file pone.0224313.s001.docx]

**S1 File: References for included and excluded systematic reviews (n=155), and practice guidelines (n=10)**

Included Reviews (n=101)

1. Abou El Senoun G, Dowswell T, Mousa HA. Planned home versus hospital care for preterm prelabour rupture of the membranes (PPROM) prior to 37 weeks' gestation. Cochrane Database Syst Rev, 2014; Issue 4. Art. No.: CD008053
2. Alfirevic Z, Kelly AJ, Dowswell T. Intravenous oxytocin alone for cervical ripening and induction of labour. Cochrane Database Syst Rev, 2009; Issue 4. Art. No.: CD003246.
3. Alfirevic Z, Aflaifel N, Weeks A. Oral misoprostol for induction of labour. Cochrane Database Syst Rev, 2014; Issue 6. Art. No.: CD001338.
4. Alfirevic Z, Keeney E, Dowswell T, Welton NJ, Medley N, Dias S, Leanne V Jones LV, Gillian Gyte, Caldwell DM. Which method is best for the induction of labour? A systematic review, network meta-analysis and cost-effectiveness analysis. Health Technol Assess, 2016;20(65):1-584. doi: 10.3310/hta20650
5. Alfirevic Z, Devane D, Gyte GML, Cuthbert A. Continuous cardiotocography (CTG) as a form of electronic fetal monitoring (EFM) for fetal assessment during labour. Cochrane Database Syst Rev, 2017; Issue 2. Art. No.: CD006066
6. Anim-Somuah M, Smyth RM, Jones L. Epidural versus non-epidural or no analgesia in labour. Cochrane Database Syst Rev, 2011; Issue 12. Art. No.:CD000331.
7. Bain E, Crane M, Tieu J, Han S, Crowther CA, Middleton P. Diet and exercise interventions for preventing gestational diabetes mellitus. Cochrane Database Syst Rev, 2015; Issue 4. Art. No.: CD010443
8. Barrett HL, Dekker Nitert M, Conwell LS, Callaway LK. Probiotics for preventing gestational diabetes. Cochrane Database Syst Rev, 2014; Issue 2. Art. No.: CD009951
9. Bohren MA, Hofmeyr GJ, Sakala C, Fukuzawa RK, Cuthbert A. Continuous support for women during childbirth. Cochrane Database Syst Rev, 2017; Issue 7. Art. No.: CD003766.
10. Bond DM, Middleton P, Levett KM, van der Ham DP, Crowther CA, Buchanan SL, Morris J. Planned early birth versus expectant management for women with preterm prelabour rupture of membranes prior to 37 weeks' gestation for improving pregnancy outcome. Cochrane Database Syst Rev, 2017; Issue 3. Art. No.: Cd004735
11. Boulvain M, Stan C, Irion O. Elective delivery in diabetic pregnant women. Cochrane Database Syst Rev, 2009; Issue 4. Art. No.: Cd001997
12. Boulvain M, Irion O, Doswell T, Thornton JG. Induction of labour at or near term for suspected fetal macrosomia. Cochrane Database Syst Rev, 2016; Issue 5. Art. No.: Cd000938
13. Bricker L, Luckas M. Amniotomy alone for induction of labour. Cochrane Database Syst Rev, 2000; Issue 4. Art. No.: CD002862
14. Bricker L, Medley N, Pratt JJ. Routine ultrasound in late pregnancy (after 24 weeks' gestation). Cochrane Database Syst Rev, 2015; Issue 6. Art. No.: CD001451
15. Brown HC, Paranjothy S, Dowswell T, Thomas J. Package of care for active management in labour for reducing caesarean section rates in low-risk women. Cochrane Database Syst Rev, 2013; Issue 9. Art. No.: CD004907.
16. Brown J, Alwan NA, West J, Brown S, McKinlay CJ, Farrar D, Crowther CA. Lifestyle interventions for the treatment of women with gestational diabetes. Cochrane Database Syst Rev, 2017a; Issue 5. Art. No.: Cd011970
17. Brown J, Grzeskowiak L, Williamson K, Downie MR, Crowther CA.Insulin for the treatment of women with gestational diabetes. Cochrane Database Syst Rev, 2017b; Issue 11. Art. No.: CD012037
18. Bugg GJ, Siddiqui F, Thornton JG. Oxytocin versus no treatment or delayed treatment for slow progress in the first stage of spontaneous labour. Cochrane Database Syst Rev, 2013; Issue 6. Art. No.: CD007123
19. Catling CJ, Medley N, Foureur M, Ryan C, Leap N, Teate A, Homer CSE. Group versus conventional antenatal care for women. Cochrane Database Syst Rev, 2015; Issue 2. Art. No.: CD007622.
20. Ceysens G, Rouiller D, Boulvain M. Exercise for diabetic pregnant women. Cochrane Database Syst Rev, 2006; Issue 3. Art No.: CD004225.
21. Cluett ER, Burns E. Immersion in water in labour and birth. Cochrane Database Syst Rev, 2009; Issue 2. Art. No.: CD000111
22. Cluver C, Gyte GML, Sinclair M, Dowswell T, Hofmeyr GJ. Interventions for helping to turn term breech babies to head first presentation when using external cephalic version. Cochrane Database Syst Rev; Issue 2015. Art. No.: CD000184
23. Costley PL, East CE. Oxytocin augmentation of labour in women with epidural analgesia for reducing operative deliveries. Cochrane Database Syst Rev, 2013; Issue 7. Art. No.: CD009241
24. Dawood F, Dowswell T, Quenby S. Intravenous fluids for reducing the duration of labour in low risk nulliparous women. Cochrane Database Syst Rev, 2013; Issue 6. Art. No.: CD007715.
25. De-Regil LM, Palacios C, Lombardo LK, Peña‐Rosas JP. Vitamin D supplementation for women during pregnancy. Cochrane Database Syst Rev, 2016; Issue 1. Art. No.: CD008873
26. Devane D, Lalor JG, Daly S, McGuire W, Cuthbert A, Smith V. Cardiotocography versus intermittent auscultation of fetal heart on admission to labour ward for assessment of fetal wellbeing. Cochrane Database Syst Rev, 2017; Issue 1. Art. No.: CD005122
27. Dodd JM, Grivell RM, CM OB, Dowswell T, Deussen AR. Prenatal administration of progestogens for preventing spontaneous preterm birth in women with a multiple pregnancy. Cochrane Database Syst Rev, 2017; Issue 10. Art. No.: CD012024
28. Dodd JM, Deussen AR, Grivell RM, Crowther CA. Elective birth at 37 weeks’ gestation for women with an uncomplicated twin pregnancy. Cochrane Database Syst Rev, 2014; Issue 2. Art. No.: CD003582
29. Dodd JM, Dowswell T, Crowther CA. Specialised antenatal clinics for women with a multiple pregnancy for improving maternal and infant outcomes. Cochrane Database Syst Rev, 2015; Issue 11. Art. No.: CD005300
30. Dowswell T, Carroli G, Duley L, Gates S, Gulmezoglu AM, Khan-Neelaofur D, Piaggio G. Alternative versus standard packages of antenatal care for low-risk pregnancy. Cochrane Database Syst Rev, 2015; Issue 7. Art. No.: CD000934
31. Dowswell T, Bedwell C, Lavender T, Neilson JP. Transcutaneous electrical nerve stimulation (TENS) for pain management in labour. Cochrane Database Syst Rev, 2009a; Issue 2. Art. No.: CD007214.
32. Dowswell T, Middleton P, Weeks A. Antenatal day care units versus hospital admission for women with complicated pregnancy. Cochrane Database Syst Rev, 2009b; Issue 4. Art. No.: CD001803
33. East CE, Begg L, Colditz PB, Lau R .Fetal pulse oximetry for fetal assessment in labour . Cochrane Database Syst Rev, 2014; Issue 10. Art. No.: CD004075
34. Falavigna M, Schmidt MI, Trujillo J, Alves LF, Wendland ER, Torloni MR, Colagiuri S, Duncan BB. Effectiveness of gestational diabetes treatment: a systematic review with quality of evidence assessment. Diabetes Res Clin Pract, 2012;98(3):396-405. doi: 10.1016/j.diabres.2012.09.002
35. Ghosh A, Lattey KR, Kelly AJ. Nitric oxide donors for cervical ripening and induction of labour. Cochrane Database Syst Rev, 2016; Issue 12. Art. No.: CD006901
36. Grivell RM, Alfirevic Z, Gyte GML, Devane D. Antenatal cardiotocography for fetal assessment. Cochrane Database Syst Rev, 2015; Issue 9. Art. No.: CD007863
37. Gulmezoglu AM, Crowther CA, Middleton P, Heatley E. Induction of labour for improving birth outcomes for women at or beyond term. Cochrane Database Syst Rev, 2012; Issue 6. Art. No.: CD004945.
38. Han S, Crowther CA, Middleton P. Interventions for pregnant women with hyperglycaemia not meeting gestational diabetes and type 2 diabetes diagnostic criteria. Cochrane Database Syst Rev, 2012a; Issue 1. Art. No.: CD009037
39. Han S, Middleton P, Crowther CA. Exercise for pregnant women for preventing gestational diabetes mellitus. Cochrane Database Syst Rev, 2012b; Issue 7. Art. No.: CD009021
40. Hapangama D, Neilson JP. Mifepristone for induction of labour. Cochrane Database Syst Rev, 2009; Issue 3. Art. No.: CD002865
41. Heazell AE, Whitworth M, Duley L, Thornton JG. Use of biochemical tests of placental function for improving pregnancy outcome. Cochrane Database Syst Rev, 2015; Issue 11. Art. No.: CD011202
42. Hodnett ED, Fredericks S, Weston J. Support during pregnancy for women at increased risk of low birthweight babies. Cochrane Database Syst Rev, 2010; Issue 6. Art. No.: CD000198
43. Hodnett ED, Downe S, Walsh D, Weston J. Alternative versus conventional institutional settings for birth. Cochrane Database Syst Rev, 2012; Issue 8. Art. No.: CD000012
44. Hofmeyr GJ, Gulmezoglu AM, Pileggi C. Vaginal misoprostol for cervical ripening and induction of labour. Cochrane Database Syst Rev, 2010; Issue 10. Art. No.: CD000941
45. Hofmeyr GJ, Kulier R. Piracetam for fetal distress in labour. Cochrane Database Syst Rev, 2012a; Issue 6. Art. No.: CD001064
46. Hofmeyr GJ, Lawrie TA . Amnioinfusion for potential or suspected umbilical cord compression in labour. Cochrane Database Syst Rev, 2012b; Issue 1. Art. No.: CD000013
47. Hofmeyr GJ, Xu H, Eke AC. Amnioinfusion for meconium-stained liquor in labour. Cochrane Database Syst Rev, 2014; Issue 1. Art. No.: CD000014
48. Hofmeyr GJ, Kulier R, West HM. External cephalic version for breech presentation at term. Cochrane Database Syst Rev, 2015; Issue 4. Art. No.: CD000083
49. Hofmeyr GJ, Vogel JP, Cuthbert A, Singata M. Fundal pressure during the second stage of labour. Cochrane Database Syst Rev, 2017; Issue 3. Art. No.: CD006067
50. Horey D, Weaver J, Russell H. Information for pregnant women about caesarean birth. Cochrane Database Syst Rev, 2004; Issue 1. Art. No.: CD003858
51. Horey D, Kealy M, Davey MA, Small R, Crowther CA. Interventions for supporting pregnant women's decision-making about mode of birth after a caesarean. Cochrane Database Syst Rev, 2013; Issue 7. Art. No.: CD010041
52. Hutton EK, Hofmeyr GJ, Dowswell T. External cephalic version for breech presentation before term. Cochrane Database Syst Rev, 2015; Issue 7. Art. No.: CD000084.
53. Jozwiak M, Bloemenkamp KW, Kelly AJ, Mol BW, Irion O, Boulvain M. Mechanical methods for Induction of Labour. Cochrane Database Syst Rev, 2012; Issue 3. Art. No.: CD001233
54. Kavanagh J, Kelly AJ, Thomas J. Breast stimulation for cervical ripening and induction of labour. Cochrane Database Syst Rev, 2005; Issue 3. Art. No.: CD003392
55. Kavanagh J, Kelly AJ, Thomas J. Hyaluronidase for cervical ripening and induction of labour. Cochrane Database Syst Rev, 2006; Issue 2. Art. No.: CD003097
56. Kelly AJ, Kavanagh J, Thomas J. Relaxin for cervical ripening and induction of labour. Cochrane Database Syst Rev, 2001; Issue 2. Art. No.: CD003103
57. Kelly AJ, Kavanagh J, Thomas J. Castor oil, bath and/or enema for cervical priming and induction of labour. Cochrane Database Syst Rev, 2013; Issue 7. Art. No.: CD003099
58. Khunpradit S, Lumbiganon P, Laopaiboon M. Admission tests other than cardiotocography for fetal assessment during labour. Cochrane Database Syst Rev, 2011a; Issue 6. Art. No.: CD008410
59. Khunpradit S, Tavender E, Lumbiganon P, Laopaiboon M, Wasiak J, Gruen RL. Non-clinical interventions for reducing unnecessary caesarean section. Cochrane Database Syst Rev, 2011b; Issue 6. Art. No.: CD005528
60. Kobayashi S, Hanada N, Matsuzaki M, Takehara K, Ota E, Sasaki H, Nagata C, Mori R*.* Assessment and support during early labour for improving birth outcomes. Cochrane Database Syst Rev, 2017; Issue 4. Art. No.: CD011516
61. Lalor JG, Fawole B, Alfirevic Z, Devane D. Biophysical profile for fetal assessment in high risk pregnancies. Cochrane Database Syst Rev, 2008; Issue 1. Art. No.: CD000038
62. Lauzon L, Hodnett E. Labour assessment programs to delay admission to labour wards. Cochrane Database Syst Rev, 2001; Issue 3. Art. No.: CD000936
63. Lavender T, Hart A, Smyth RM. Effect of partogram use on outcomes for women in spontaneous labour at term. Cochrane Database Syst Rev, 2013; Issue 7. Art. No.: CD005461
64. Lundgren I, Smith V, Nilsson C, Vehvilainen-Julkunen K, Nicoletti J, Devane D, Bernloehr A, van Limbeek E, Lalor J, Begley C. Clinician-centred interventions to increase vaginal birth after caesarean section (VBAC): a systematic review. BMC Pregnancy Childbirth, 2015;15(16):1-9. doi: 10.1186/s12884-015-0441-3
65. Lutomski JE, Meaney S, Greene RA, Ryan AC, Devane D. Expert systems for fetal assessment in labour. Cochrane Database Syst Rev, 2015; Issue 4. Art. No.: CD010708.
66. Madden K, Middleton P, Cyna AM, Matthewson M, Jones L. Hypnosis for pain management during labour and childbirth. Cochrane Database Syst Rev, 2016; Issue 5. Art. No.: CD009356
67. Madhuvrata P, Govinden G, Bustani R, Song S, Farrell TA. Prevention of gestational diabetes in pregnant women with risk factors for gestational diabetes: a systematic review and meta-analysis of randomised trials. Obstet Med, 2015;8(2):68-85. doi: 10.1177/1753495X15576673
68. Malin GL, Bugg GJ, Thornton J, Taylor MA, Grauwen N, Devlieger R, Kardel KR, Kubli M, Tranmer JE, Jones NW*.* Does oral carbohydrate supplementation improve labour outcome? A systematic review and individual patient data meta-analysis. BJOG, 2016;123:510–517. doi: 10.1111/1471-0528.13728
69. Mangesi L, Hofmeyr GJ, Smith V, Smyth RMD. Fetal movement counting for assessment of fetal wellbeing. Cochrane Database Syst Rev, 2015; Issue 10. Art. No.: CD004909
70. Martis R, Emilia O, Nurdiati DS, Brown J. Intermittent auscultation (IA) of fetal heart rate in labour for fetal well-being. Cochrane Database Syst Rev, 2017; Issue 2. Art. No.: CD008680
71. Meher S, Duley L. Exercise or other physical activity for preventing pre-eclampsia and its complications. Cochrane Database Syst Rev, 2006a; Issue 2. Art. No.: CD005942
72. Meher S, Duley L. Garlic for preventing pre-eclampsia and its complications. Cochrane Database Syst Rev, 2006b; Issue 3. Art. No.: CD006065
73. Meher S, Duley L. Rest during pregnancy for preventing pre-eclampsia and its complications in women with normal blood pressure. Cochrane Database Syst Rev, 2006c; Issue 2. Art. No.: CD005939
74. Middleton P, Shepherd E, Flenady V, McBain RD, Crowther CA. Planned early birth versus expectant management (waiting) for prelabour rupture of membranes at term (37 weeks or more). Cochrane Database Syst Rev, 2017; Issue 1. Art. No.: CD005302
75. Muktabhant B, Lawrie TA, Lumbiganon P, Laopaiboon M. Diet or exercise, or both, for preventing excessive weight gain in pregnancy. Cochrane Database Syst Rev, 2015; Issue 6. Art. No.: CD007145
76. Neilson JP. Fetal electrocardiogram (ECG) for fetal monitoring during labour. Cochrane Database Syst Rev, 2015; Issue 12. Art. No.: CD000116
77. Ota E, Mori R, Middleton P, Tobe‐Gai R, Mahomed K, Miyazaki C, Bhutta ZA. Zinc supplementation for improving pregnancy and infant outcome. Cochrane Database Syst Rev, 2015; Issue 2. Art. No.: CD000230
78. Pattinson RC, Cuthbert A, Vannevel V. Pelvimetry for fetal cephalic presentations at or near term for deciding on mode of delivery. Cochrane Database Syst Rev, 2017; Issue 3. Art. No.: CD000161
79. Phipps H, de Vries B, Hyett J, Osborn DA. Prophylactic manual rotation for fetal malposition to reduce operative delivery. Cochrane Database Syst Rev, 2014; Issue 12. Art. No.: CD009298
80. Rafael TJ, Berghella V, Alfirevic Z*.* Cervical stitch (cerclage) for preventing preterm birth in multiple pregnancy. Cochrane Database Syst Rev, 2014; Issue 9. Art. No.: CD009166
81. Raman P, Shepherd E, Dowswell T, Middleton P, Crowther CA*.* Different methods and settings for glucose monitoring for gestational diabetes during pregnancy. Cochrane Database Syst Rev, 2017; Issue 10. Art. No.: CD011069
82. Rumbold A, Ota E, Nagata C, Shahrook S, Crowther CA. Vitamin C supplementation in pregnancy. Cochrane Database Syst Rev, 2015a; Issue 9. Art. No.: CD004072
83. Rumbold A, Ota E, Hori H, Miyazaki C, Crowther CA. Vitamin E supplementation in pregnancy. Cochrane Database Syst Rev, 2015b; Issue 9. Art. No.: CD004069
84. Smith CA. Homoeopathy for induction of labour. Cochrane Database Syst Rev, 2003; Issue 4. Art. No.: CD003399
85. Smith CA, Collins CT, Cyna AM, Crowther CA. Complementary and alternative therapies for pain management in labour. Cochrane Database Syst Rev, 2006; Issue 4. Art. No.: CD003521
86. Smith CA, Collins CT, Crowther CA, Levett KM. Acupuncture or acupressure for pain management in labour. Cochrane Database Syst Rev, 2011a; Issue 7. Art. No.: CD009232
87. Smith CA, Collins CT, Crowther CA*.* Aromatherapy for pain management in labour. Cochrane Database Syst Rev, 2011b; Issue 7. Art. No.: CD009215
88. Smith CA, Levett KM, Collins CT, Crowther CA. Relaxation techniques for pain management in labour. Cochrane Database Syst Rev, 2011c; Issue 12. Art. No.: CD009514
89. Smith CA, Levett KM, Collins CT, Jones L. Massage, reflexology and other manual methods for pain management in labour. Cochrane Database Syst Rev, 2012; Issue 2. Art. No.: CD009290
90. Smith CA, Armour M, Dahlen HG. [update]; Acupuncture or acupressure for induction of labour. Cochrane Database Syst Rev, 2017; Issue 10. Art. No.: CD002962
91. Smyth RMD, Markham C, Dowswell T. Amniotomy for shortening spontaneous labour. Cochrane Database Syst Rev, 2013; Issue 6. Art. No.: CD006167
92. Stock SJ, Bricker L, Norman JE, West HM. Immediate versus deferred delivery of the preterm baby with suspected fetal compromise for improving outcomes. Cochrane Database Syst Rev, 2016; Issue 7. Art. No.: CD008968
93. Thomas J, Fairclough A, Kavanagh J, Kelly AJ. Vaginal prostaglandin (PGE2 and PGF2a) for induction of labour at term. Cochrane Database Syst Rev, 2014; Issue 6. Art. No.: CD003101
94. Tieu J, Shepherd E, Middleton P, Crowther CA. Dietary advice interventions in pregnancy for preventing gestational diabetes mellitus. Cochrane Database Syst Rev, 2017; Issue 1. Art. No.: CD006674.
95. Till SR, Everetts D, Haas DM. Incentives for increasing prenatal care use by women in order to improve maternal and neonatal outcomes. Cochrane Database Syst Rev, 2015; Issue 12. Art. No.: CD009916
96. Torvaldsen S, Roberts CL, Bell JC, Raynes-Greenow CH*.* Discontinuation of epidural analgesia late in labour for reducing the adverse delivery outcomes associated with epidural analgesia. Cochrane Database Syst Rev, 2004; Issue 4. Art. No.: CD004457
97. Vogel JP, Osoti AO, Kelly AJ, Livio S, Norman JE, Alfirevic Z. Pharmacological and mechanical interventions for labour induction in outpatient settings. Cochrane Database Syst Rev, 2017; Issue 9. Art. No.: CD007701
98. Weaver J, Browne J, Aras-Payne A, Magill‐Cuerden J. A comprehensive systematic review of the impact of planned interventions offered to pregnant women who have requested a caesarean section as a result of tokophobia (fear of childbirth). JBI Database System Rev Implement Rep, 2013;11(1):70-122. doi: 10.11124/jbisrir-2013-606
99. Wei S, Wo BL, Qi HP, Xu H, Luo ZC, Roy C, Fraser, WD. Early amniotomy and early oxytocin for prevention of, or therapy for, delay in first stage spontaneous labour compared with routine care. Cochrane Database Syst Rev, 2013; Issue 8. Art. No.: CD006794
100. Whitworth M, Bricker L, Mullan C. Ultrasound for fetal assessment in early pregnancy. Cochrane Database Syst Rev, 2015; Issue 7. Art. No.: CD007058
101. Wojcieszek AM, Stock OM, Flenady V. Antibiotics for prelabour rupture of membranes at or near term. Cochrane Database Syst Rev, 2014; Issue 10. Art. No.: CD001807

**Excluded reviews (Critically low on AMSTAR-2) (n=54)**

1. Al-Mandeel H, Alhindi MY, Sauve R. Effects of intentional delivery on maternal and neonatal outcomes in pregnancies with preterm prelabour rupture of membranes between 28 and 34 weeks of gestation: A systematic review and meta-analysis. J Matern Fetal Neonatal Med, 2013;26(1):83-89. doi: 10.3109/14767058.2012.718388
2. Alwan N, Tuffnell DJ, West J. Treatments for gestational diabetes. Cochrane Database Syst Rev, 2009; Issue 3. Art.No.: CD003395
3. Blix E, Brurberg KG, Reierth E, Reinar LM, Øian P. ST waveform analysis versus cardiotocography alone for intrapartum fetal monitoring: a systematic review and meta-analysis of randomized trials. Acta Obstet Gynecol Scand, 2016;95(1):16-27. doi: 10.1111/aogs.12828
4. Boulvain M, Stan CM, Irion O. Membrane sweeping for induction of labour. Cochrane Database Syst Rev, 2005; Issue 1. Art. No.: CD000451
5. Boulvain M, Kelly A, Irion O. Intracervical prostaglandins for induction of labour. Cochrane Database Syst Rev, 2008; Issue 1. Art. No.: CD006971
6. Brancato RM, Church S, Stone PW. A meta-analysis of passive descent versus immediate pushing in nulliparous women with epidural analgesia in the second stage of labor. J Obstet Gynecol Neonatal Nurs. 2008;37(1):4-12. doi: 10.1111/j.1552-6909.2007.00205.x
7. Cantor AG, Bougatsos C, Dana T, Blazina I, McDonagh M. Routine iron supplementation and screening for iron deficiency anemia in pregnancy: a systematic review for the U.S. Preventive Services Task Force. Ann Intern Med, 2015;162(8):566-576. doi: 10.7326/M14-2932.
8. Catling-Paull C, Johnston R, Ryan C, Foureur MJ, Homer CS. Clinical interventions that increase the uptake and success of vaginal birth after caesarean section: a systematic review. J Adv Nurs, 2011;67(8):1646-61. doi: 10.1111/j.1365-2648.2011.05635.x
9. Caughey AB, Sundaram V, Kaimal AJ, Cheng YW, Gienger A, Little SE, Lee JF, Wong L, Shaffer BL, Tran SH, Padula A, McDonald KM, Long EF, Owens DK, Bravata DM. Maternal and neonatal outcomes of elective induction of labor. Evid Rep Technol Assess (Full Rep), 2009;176:1-257.
10. Chaillet N, Dumont A. Evidence-based strategies for reducing cesarean section rates: a meta-analysis. Birth, 2007;34(1):53-64. doi: 10.1111/j.1523-536X.2006.00146.x
11. Dodd JM, Grivell RM, Crowther CA, Robinson JS. Antenatal interventions for overweight or obese pregnant women: a systematic review of randomised trials. BJOG, 2010;117(11):1316-1326. doi: 10.1111/j.1471-0528.2010.02540.x
12. Domenjoz I, Kayser B, Boulvain M. Effect of physical activity during pregnancy on mode of delivery. Am J Obstet Gynecol, 2014;211(4):401.e1-11. doi: 10.1016/j.ajog.2014.03.030
13. Duley L, Gulmezoglu AM, Henderson-Smart DJ, Chou D. Magnesium sulphate and other anticonvulsants for women with pre-eclampsia. Cochrane Database Syst Rev, 2010; Issue 11. Art. No.: CD000025
14. Fortier JH, Godwin M. Doula support compared with standard care: Meta-analysis of the effects on the rate of medical interventions during labour for low-risk women delivering at term. Can Fam Physician 2015;61(6):e284-e292.
15. Hartling L, Chari R, Friesen C, Vandermeer B, Lacaze-Masmonteil T. A systematic review of intentional delivery in women with preterm prelabor rupture of membranes. J Matern Fetal Neonatal Med, 2006;19(3):177-187. doi: 10.1080/14767050500451470
16. Howarth GR, Botha DJ. Amniotomy plus intravenous oxytocin for induction of labour. Cochrane Database Syst Rev, 2001; Issue 3. Art. No.: CD003250.
17. Hunter S, Hofmeyr GJ, Kulier R. Hands and knees posture in late pregnancy or labour for fetal malposition (lateral or posterior). Cochrane Database Syst Rev, 2007; Issue 4. Art. No.: CD001063
18. Hutton E, Mozurkewich E. Extra-amniotic prostaglandin for induction of labour. Cochrane Database Syst Rev, 2001; Issue 2. Art. No.: CD003092
19. Hutton EK, Kasperink M, Rutten M, Reitsma A, Wainman B. Sterile water injection for labour pain: a systematic review and meta-analysis of randomised controlled trials. BJOG, 2009;116(9):1158-1166. doi: 10.1111/j.1471-0528.2009.02221.x
20. Kehl S, Weiss C, Rath W. Balloon catheters for induction of labor at term after previous cesarean section: a systematic review. Eur J Obstet Gynecol Reprod Biol, 2016;204:44-50. doi: 10.1016/j.ejogrb.2016.07.505
21. Kraemer DF, Berlin M, Guise JM. The relationship of health care delivery system characteristics and legal factors to mode of delivery in women with prior cesarean section: a systematic review. Women's Health Issues, 2004;14(3):94-103. doi: 10.1016/j.whi.2004.04.002
22. Magro-Malosso ER, Saccone G, Di Mascio D, Di Tommaso M, Berghella V. Exercise during pregnancy and risk of preterm birth in overweight and obese women: a systematic review and meta-analysis of randomized controlled trials. Acta Obstet Gynecol Scand, 2017a;96(3):263-273. doi: 10.1111/aogs.13087
23. Magro-Malosso ER, Saccone G, Chen M, Navathe R, Di Tommaso M, Berghella V. Induction of labour for suspected macrosomia at term in non-diabetic women: a systematic review and meta-analysis of randomized controlled trials. BJOG, 2017b;124(3):414-421. doi: 10.1111/aogs.13087
24. Makvandi S, Mirzaiinajmabadi K, Sadeghi R, Mahdavian M, Karimi L. Meta-analysis of the effect of acupressure on duration of labor and mode of delivery. Int J Gynaecol Obstet, 2016; 135(1). doi: 10.1016/j.ijgo.2016.04.017
25. McDonagh MS, Osterweil P, Guise JM. The benefits and risks of inducing labour in patients with prior caesarean delivery: a systematic review. BJOG, 2005;112(8):1007-101. doi: 10.1111/j.1471-0528.2005.00623.x
26. Mishanina E, Rogozinska E, Thatthi T, Uddin-Khan R, Khan KS, Meads C. Use of labour induction and risk of cesarean delivery: a systematic review and meta-analysis. CMAJ, 2014;186(9):665-673. doi: 10.1503/cmaj.130925
27. Nilsson C, Lundgren I, Smith V, Vehvilainen-Julkunen K, Nicoletti J, Devane D, Bernloehr A, van Limbeek E, Lalor J, Begley C. Women-centred interventions to increase vaginal birth after caesarean section (VBAC): A systematic review. Midwifery, 2015;31(7):657-63. doi: 10.1016/j.midw.2015.04.003
28. O'Brien CM, Grivell RM, Dodd JM. Systematic review of antenatal dietary and lifestyle interventions in women with a normal body mass index. Acta Obstet Gynecol Scand, 2016;95(3):259-69. doi: 10.1111/aogs.12829
29. Oteng-Ntim E, Varma R, Croker H, Poston L, Doyle P. Lifestyle interventions for overweight and obese pregnant women to improve pregnancy outcome: systematic review and meta-analysis. BMC Med, 2012;10:47. doi: 10.1186/1741-7015-10-47
30. Pérez-López FR, Pasupuleti V, Mezones-Holguin E, Benites-Zapata VA, Thota P, Deshpande A, Hernandez A. Effect of vitamin D supplementation during pregnancy on maternal and neonatal outcomes: a systematic review and meta-analysis of randomized controlled trials. Fertil Steril, 2015;103(5):1278-1288. doi: 10.1016/j.fertnstert.2015.02.019
31. Poolsup N, Suksomboon N, Amin M. Effect of treatment of gestational diabetes mellitus: a systematic review and meta-analysis. PLoS One, 2014;9(3):e92485. doi: 10.1371/journal.pone.0092485
32. Poyatos-León R, Garcia-Hermoso A, Sanabria-Martinez G, Alvarez-Bueno C, Sanchez-Lopez M, Martinez-Vizcaino V. Effects of exercise during pregnancy on mode of delivery: A meta-analysis. Acta Obstet Gynecol Scand, 2015;94(10):1039-1047. doi: 10.1111/aogs.12675
33. Rossi AC, Prefumo F. Pregnancy outcomes of induced labor in women with previous cesarean section: A systematic review and meta-analysis. Arch Gynecol Obstet, 2015;291:273–280. doi: 10.1007/s00404-014-3444-9.
34. Rossignol M, Chaillet N, Boughrassa F, Moutquin JM. Interrelations between four antepartum obstetric interventions and cesarean delivery in women at low risk: a systematic review and modeling of the cascade of interventions. Birth, 2014;41(1):70-78. doi: 10.1111/birt.12088
35. Saccone G, Berghella V. Induction of labor at full term in uncomplicated singleton gestations: a systematic review and metaanalysis of randomized controlled trials. Am J Obstet Gynecol, 2015;213(5):629-636. doi: 10.1016/j.ajog.2015.04.004
36. Saccone G, Schuit E, Amer-Wåhlin I, Xodo S, Berghella V*.* Electrocardiogram ST analysis during Labor: A Systematic Review and Meta-analysis of Randomized Controlled Trials. Obstet Gynecol, 2016;127(1):127-135. doi: 10.1097/AOG.0000000000001198
37. Salmelin A, Wiklund I, Bottinga R, Brorsson B, Ekman-Ordeberg G, Grimfors EE, Hanson U, Blom M, Persson E. Fetal monitoring with computerized ST analysis during labor: a systematic review and meta-analysis. Acta Obstet Gynecol Scand, 2013;92(1):28-39. doi: 10.1111/aogs.12009
38. Sanchez-Ramos L, Bernstein S, Kaunitz AM. Expectant management versus labor induction for suspected fetal macrosomia: a systematic review. Obstet Gynecol, 2002;100(5):997-1002. doi: 10.1016/S0029-7844(02)02321-9
39. Sanchez-Ramos L, Olivier F, Delke I, Kaunitz AM. Labor induction versus expectant management for postterm pregnancies: a systematic review with meta-analysis. Obstet Gynecol, 2003;101(6):1312-1318.
40. Schuit E, Amer-Wahlin I, Ojala K, Vayssière C, Westerhuis ME, Maršál K, Tekay A, Saade GR, Visser GH, Groenwold RH, Moons KG, Mol BW, Kwee A. Effectiveness of electronic fetal monitoring with additional ST analysis in vertex singleton pregnancies at >36 weeks of gestation: an individual participant data metaanalysis. Am J Obstet Gynecol, 2013;208(3):187.e1-187.e13. doi: 10.1016/j.ajog.2013.01.028
41. Stoll K, Swift E, Fairbrother N, Nethery E, Janssen P*.* A systematic review of nonpharmacological prenatal interventions for pregnancy-specific anxiety and fear of childbirth. Birth, 2017; 45(Suppl 1): 1–12. doi: 10.1111/birt.12316
42. Thangaratinam S, Rogozińska E, Jolly K, Glinkowski S, Duda W, Borowiack E, Rosebloom T, Tomlinson J, Walczak J, Kunz R, Mol BW, Coomarasamy A, Khan KS. Interventions to reduce or prevent obesity in pregnant women: a systematic review. Health Technol Assess, 2012;16(31):iii-iv, 1-191. doi: 10.3310/hta16310
43. Thomas J, Kelly AJ, Kavanagh J. Oestrogens alone or with amniotomy for cervical ripening or induction of labour. Cochrane Database Syst Rev, 2001, Issue 4. Art. No.: CD003393
44. Thorne-Lyman A, Fawzi WW. Vitamin D during Pregnancy and Maternal, Neonatal and Infant Health Outcomes: A Systematic Review and Meta-analysis. Paediatr Perinat Epidemiol, 2012;26(Suppl.1):75–90. doi: 10.1111/j.1365-3016.2012.01283.x.
45. Tuuli MG, Frey HA, Odibo AO, Macones GA, Cahill AG. Immediate compared with delayed pushing in the second stage of labor: a systematic review and meta-analysis. Obstet Gynecol, 2012;120(3):660-668. doi: 10.1097/AOG.0b013e3182639fae
46. Waldenström U, Turnbull, D. A systematic review comparing continuity of midwifery care with standard maternity services. Br J Obstet Gynaecol. BJOG, 1998;105(11):1160-70. doi: 10.1111/j.1471-0528.1998.tb09969.x
47. Walker R, Turnbull D, Wilkinson C. Strategies to address global cesarean section rates: a review of the evidence. Birth, 2002;29(1):28-39. doi: 10.1046/j.1523-536X.2002.00153.x
48. Walker KF, Malin G, Wilson P, Thornton JG. Induction of labour versus expectant management at term by subgroups of maternal age: an individual patient data meta-analysis. Eur J Obstet Gynecol Reprod Biol, 2016;197:1-5. doi: 10.1016/j.ejogrb.2015.11.004
49. Wei SQ, Luo ZC, Xu H, Fraser WD. The effect of early oxytocin augmentation in labor: a meta-analysis.Obstet Gynecol, 2009;114(3):641-649. doi: 10.1097/AOG.0b013e3181b11cb8
50. Wennerholm UB, Hagberg H, Brorsson B, Bergh C. Induction of labor versus expectant management for post-date pregnancy: is there sufficient evidence for a change in clinical practice?. Acta Obstet Gynecol Scand, 2009;88(1):6-17. doi: 10.1080/00016340802555948.
51. Whitworth M, Quenby S, Cockerill RO, Dowswell T. Specialised antenatal clinics for women with a pregnancy at high risk of preterm birth (excluding multiple pregnancy) to improve maternal and infant outcomes. Cochrane Database Syst Rev, 2011; Issue 9. Art. No.: CD006760.
52. Witkop CT, Neale D, Wilson LM, Bass EB, Nicholson WK. Active compared with expectant delivery management in women with gestational diabetes: a systematic review. Obstet Gynecol, 2009;113(1):206-217. doi: 10.1097/AOG.0b013e31818db36f
53. Wood S, Cooper S, Ross S. Does induction of labor increase the risk for caesarean section? A systematic review and meta-analysis of trials in women with intact membranes. Obstet Gynecol Surv, 2014;69(9):519-521. doi: 10.1097/OGX.0000000000000100
54. Xu H, Hofmeyr J, Roy C, Fraser W. Intrapartum amnioinfusion for meconium-stained amniotic fluid: a systematic review of randomised controlled trials. BJOG, 2007;114(4):383-390. doi: 10.1111/j.1471-0528.2007.01262.x

**Included Guidelines (n=10)**

1. ACOG. Practice Bulletin No. 161: External Cephalic Version. Obstet Gynecol, 2016;127(2):e54-61. doi: 10.1097/AOG.0000000000001312
2. ACOG Practice Bulletin No. 2 Vaginal Birth after previous cesarean delivery. October 1998. Replaced by update: ACOG Updated Guidance No. 184. Obstet Gynecol, 2017;130(5):e217-e233. doi: 10.1097/AOG.0000000000002398.
3. ACOG Committee Opinion No. 687: Approaches to limit intervention during labor and birth. Obstet Gynecol, 2017a, 129(2), e20-e28. doi: 10.1097/AOG.0000000000001905
4. Caughey AB. Safe Prevention of Primary Cesarean Delivery in the United States: Why and How? Clin Obstet Gynecol, 2015;58(2):207-210. doi: 10.1097/GRF.0000000000000111
5. FIGO Working Group. Best practice advice on the 10-Group Classification System for cesarean deliveries. Int J Gynaecol Obstet, 2016;135(2):232-233. doi: 10.1016/j.ijgo.2016.08.001.
6. Hauk L. Planning for Labor and Vaginal Birth After Cesarean Delivery: Guidelines from the AAFP. Am Fam Physician, 2015;91(3):197-8.
7. Kotaska A, Menticoglou S, Gagnon R. Vaginal delivery of breech presentation. J Obstet Gynaecol Can, 2009;31(6):557-566. doi: 10.1016/S1701-2163(16)34221-9.
8. Mandruzzato G, Alfirevic Z, Chervenak F, Gruenebaum A, Heimstad R, Heinonen S, Levene M, Salvesen K, Saugstad O, Skupski D, Thilaganathan B. Guidelines for the management of post-term pregnancy. J Perinat Med, 2010;38(2):111-9. doi: 10.1515/JPM.2010.057
9. Sentilhes L, Vayssière C, Beucher G, Deneux-Tharaux C, Deruelle P, Diemunsch P, Gallot D, Haumonté JB, Heimann S, Kayem G, Lopez E, Parant O, Shmitz T, Sellier Y, Rozenberg P, d’Ercole C. Delivery for women with a previous caesarean: guidelines for clinical practice from the French College of Gynecologists and Obstetricians (CNGOF). Eur J Obstet Gynecol Reprod Biol, 2013;170(1):25-32. Art. No.: doi: 10.1016/j.ejogrb.2013.05.015
10. SOGC. Guidelines for vaginal birth after previous caesarean birth. Number 155 (Replaces guideline Number 147). J Obstet Gynaecol Ca, 2005;27(2):164–174 . doi: 10.1016/S1701-2163(16)30188-8.
