# Supplementary material for: Antenatal and intrapartum interventions for reducing caesarean section, promoting vaginal birth, and reducing fear of childbirth: An overview of systematic reviews
Source: PLoS One. 2019 Oct 24;14(10):e0224313. doi: 10.1371/journal.pone.0224313 (PMC6812784; doi:10.1371/journal.pone.0224313)
Supplement: S1 Table — (DOCX) [file pone.0224313.s002.docx]

**S1 Table: Summary characteristics of the 101 included reviews**

**(Green text = statistically significant results)**

| ***Author***  ***Year*** | ***Aim*** | ***Population*** | ***Intervention versus comparator*** | ***Reported outcomes and results*** | ***AMSTAR-2*** |
| --- | --- | --- | --- | --- | --- |
| Abou El Senoun 2014 | To assess the effects of planned home versus hospital care for women with preterm prelabour rupture of the membranes (PPROM) prior to 37 weeks’ gestation on fetal, infant and maternal wellbeing. | Women with preterm PPROM before 37 weeks’ gestation with no specific maternal or fetal contraindications to expectant management. | PPROM at home *versus* PPROM in hospital | ***Caesarean section:***  RR 0.28 [0.07 to 1.15], 2 trials, n=116  ***Adverse effect:***  *Perinatal mortality (up to 7 days):* RR 1.93 [0.19 to 20.05], 1 trial, n=55  ***Cost:***  *Total hospital care cost:* SMD -0.80 [-1.41 to -0.19], 2 trials, n=116 | High  Score = 15  1 non-critical weakness on 10 |
| Alfirevic 2009 | To determine the effects of oxytocin alone for third trimester cervical ripening and IOL in comparison with other methods of IOL or placebo/no treatment | Pregnant women and their babies. | Intravenous oxytocin alone *versus* placebo/expectant management | ***Caesarean section:***  *10.4% versus 9.0%:* **RR 1.17 [1.01 to 1.35), 24 trials, n=6620**  ***Instrumental vaginal birth:***  RR 1.06 [0.94 to 1.19], 14 trials, n=5275  ***Adverse effects:***  17 cases of serious neonatal morbidity or perinatal death: RR 0.63 [0.26 to 1.51], 10 trials, n=4816 *Maternal mortality:* RR 0.00 [0.00 to 0.00], 1 trial, n=40  ***Satisfaction:***  Women were less likely to be dissatisfied with IOL compared with expectant management (5.9% versus 13.7%): **RR 0.43 [0.33 to 0.56], 1 trial, n=5041** | Moderate  Score = 14  2 non-critical weaknesses on 5 and 10 |
| Alfirevic 2014 | To assess the use of oral misoprostol for IOL in women with a viable fetus. | Pregnant women and their babies. | Oral misoprostol *versus* placebo/no treatment | ***Caesarean section:***  **RR 0.72 [0.54 to 0.95],** 8 trials, n=1029  ***Instrumental vaginal birth:***  RR 0.65 [0.37 to 1.17], 5 trials, n=379  ***Adverse effects:***  *Serious neonatal morbidity, perinatal death or maternal mortality*: 0, 1 trial, n=80 | Low  Score = 14  1 critical flaw on 15; 1 non-critical flaw on 10 |
| Alfirevic 2016 | To assess the relative effectiveness, safety and cost-effectiveness of IOL methods and, data permitting, effects in different clinical subgroups. | Pregnant women carrying a viable fetus and who are eligible for any method of third-trimester cervical ripening or IOL. | All pharmacological (all routes and doses), mechanical and complementary methods used for IOL *versus* placebo/no intervention | ***Caesarean section:***  Vaginal PGE2 (gel): **OR 0.79 [0.65 to 0.94]**  Intracervical PGE2: **OR 0.83 [0.69 to 0.98]**  Vaginal misoprostol tablet: **< 50μg OR 0.70 [0.57 to 0.85]**  Vaginal misoprostol tablet: **≥ 50μg OR 0.73 [0.59 to 0.88]**  Oral misoprostol tablet: **≥ 50μg OR 0.72 [0.58 to 0.88]**  Titrated (low-dose) oral misoprostol solution: **OR 0.62 [0.47 to 0.80]**  Foley catheter: **OR 0.76 [0.61 to 0.95]**  Membrane sweeping: **OR 0.74 [0.53 to 0.99]**  Buccal/ sublingual Misoprostol: **OR 0.68 [0.51 to 0.89**], **586 trials, n= 96771**  ***Instrumental birth:***  Reduction with vaginal PGE2 pessary (slow release): **OR 0.72 [0.50 to 0.99]**  Foley catheter: **OR 0.68 [0.50 to 0.91]**  ***Adverse effects:***  131/611 trials reported perinatal deaths with incidence of 0.3% (94/32,248).  77/611 trials (12.6%) reported a total of 20 maternal deaths or serious morbidity [5 deaths, 14 uterine ruptures and 1 intensive care unit (ICU) admission for infection]=incidence of 0.1%.  ***Costs:***  **All methods of IOL have lower expected total costs than placebo** (no intervention) because they reduce costly outcomes (VD in > 24 hours, CS and NICU admission). 19/34 trials were included in the cost analysis. | High  Score = 15  1 non-critical weakness on 10 |
| Alfirevic 2017 | To evaluate the effectiveness and safety of continuous CTG when used as a method to monitor fetal wellbeing during labour. | Pregnant women in labour and their babies. | Continuous CTG during labour *versus* no fetal monitoring *or* IA with Pinard stethoscope *or* hand-held Doppler ultrasound device | ***Caesarean section:***  CTG vs IA overall: **RR 1.63 [1.29 to 1.33], 11 trials, n=18861**  High-risk women: **RR 1.91 [1.39 to 2.61], 6 trials, n=2069**  Low-risk women: **RR 2.06 [1.24 to 3.45],** **2 trials, n=1431**  *Mixed risk or not specified:* RR 1.14 [0.95 to 1.36], 3 trials, n=15361  ***Instrumental birth:***  **RR 1.15 [1.01 to 1.33],** **10 trials, n=18615**  ***Spontaneous vaginal birth:***  **RR 0.91 [0.86 to 0.96], 11 trials, n=18861**  ***Adverse effects:***  *Perinatal death:* RR 0.86 [0.59 to 1.24], 11 trials, n=33513 | High  Score = 16  No weaknesses |
| Anim-Somuah 2011 | To assess the effects of all modalities of epidural analgesia (including combined-spinal-epidural) on the mother and the baby, when compared with non-epidural or no pain relief during labour. | Pregnant women requesting pain relief in labour, regardless of parity and whether labour was spontaneous or induced. | All forms of epidural *versus* any form of pain relief not involving regional blockade, *or* no pain relief | ***Caesarean section:***  *Overall:* RR 1.10 [0.97 to 1.25], 27 trials, n=8417  CS for fetal distress*:* **RR 1.43 [1.03 to 1.97], 11 trials, n=4816**  *CS for dystocia:* RR 0.90 [0.73 to 1.12], 12 trials, n=5001  ***Instrumental vaginal birth:***  **RR 1.42 [1.28 to 1.57],** **23 trials, n=7935**  ***Satisfaction:***  *With pain relief:* RR 1.31 [0.84 to 2.05], 7 trials, n= 2929  *With childbirth experience:* RR 0.95 [0.87 to 1.03], 1 trial, n=332 | High  Score = 15  1 non-critical flaw on 10 |
| Bain 2015 | To assess the effects of combined diet and exercise interventions for preventing GDM and associated adverse health consequences for  women and their babies. | Pregnant women and their babies. | A diet and exercise intervention *versus* no intervention | ***Caesarean section:***  RR 0.92 [0.83 to 1.01], 7 trials, n=3246  ***Spontaneous vaginal birth:***  RR 1.25 [0.70 to 2.23], 1 trial, n=51  ***Vacuum extraction:***  RR 1.00 [0.20 to 4.93], 1 trial, n=51  ***Adverse effects:***  *Perinatal death:* RR 0.99 [0.32 to 3.07], 1 trial, n=2202 | High  Score = 15  1 non-critical flaw on 10 |
| Barrett 2014 | To assess the effects of probiotic supplementation when compared with other methods for the prevention of GDM. | Pregnant women, no DM or GDM. | Probiotic supplementation *versus* placebo | ***Caesarean section:***  *Probiotic:* RR 1.26 [0.51 to 3.11], 1 trial, n=109  ***Adverse effects:***  *Miscarriage/IUFD/Stillbirth/Neonatal death:* RR 2.00 [0.35 to 11.35], 1 study, n=256 | High  Score = 12  1 non-critical flaw on 10; no meta-analysis |
| Bohren 2017 | To assess the effects, on women and their babies, of continuous, one-to-one intrapartum support compared with usual care, in any setting. | Pregnant women in labour. | Continuous, one-to-one intrapartum support *versus* usual care | ***Caesarean section:***  Overall: **RR 0.75 [0.64 to 0.88],** **24 trials, n=15347** *Policy regarding presence of companion:* RR 0.78 [0.67 to 0.91], 22 trials, n=15175  *Support people were hospital staff:* RR 0.94 [0.84 to 1.05], 9 trials, n=10786  Support people were not hospital staff and not chosen by woman: **RR 0.61 [0.45 to 0.83], 9 trials, n=2502**  ***Spontaneous vaginal birth:***  **RR 1.08 [1.04 to 1.12], 21 trials, n=14369**  ***Instrumental birth:***  **RR 0.90 [0.85 to 0.96], 19 trials, n=14118**  ***Satisfaction:***  Negative feelings about birth experience: **RR 0.69 [0.59 to 0.79],** **11 trials, n=11133** | High  Score = 15  1 non-critical flaw on 10 |
| Bond 2017 | To assess the effect of planned early birth versus expectant management for women with preterm prelabour rupture of the membranes between 24 and 37 weeks’ gestation for fetal, infant and maternal wellbeing. | Women with PPROM before 37 weeks. | Planned early birth (IOL or CS) *versus* expectant management | ***Caesarean section:***  Overall: **RR 1.26 [1.11 to 1.44],** **12 trials, n=3620**  *CS for fetal distress:* RR 0.89 [0.66 to 1.20], 7 trials, n=2918  ***Vaginal birth:***  **RR 0.94 [0.91 to 0.97**], **12 trials, n=3618**  ***Operative vaginal birth:***  RR 0.85 [0.67 to 1.10], 4 trials, n=2685  ***Adverse effects:***  *Perinatal mortality:* RR 1.76 [0.89 to 3.50], 11 trials, n=3319 babies  *IUD:* RR 0.45 [0.13 to 1.55], 11 trials, n=3321 Neonatal death: **RR 2.55 [1.17 to 5.56], 11 trials, n=3316**  ***Maternal satisfaction:***  *With birth experience:* RR 0.99 [0.86 to 1.13], 1 trial, n=493 | High  Score = 15  1 non-critical on 10 |
| Boulvain 2001 | To assess the effect of a policy of elective delivery, as compared to expectant management, in term diabetic pregnant women, on maternal and perinatal mortality and morbidity. | Pregnant women at term, with diabetes, and their babies. | Elective delivery *versus* expectant management | ***Caesarean section:***  RR 0.81 [0.52 to 1.26], 1 trial, n=200  *Previous CS:* RR 0.53 [0.27 to 1.05], 1 trial, n=31  *No previous CS:* RR 1.28 [0.70 to 2.37], 1 trial, n=169  ***Adverse effects:***  *Perinatal mortality:* RR 0.00 [0.00 to 0.00], 1 trial, n=200 | High  Score = 12  1 non-critical flaw on 10; no meta-analysis |
| Boulvain 2016 | To assess the effects of a policy of IOL at or shortly before term (37 to 40 weeks) for suspected fetal macrosomia on the way of giving birth and maternal or perinatal morbidity. | Pregnant women with suspected fetal macrosomia and their babies. | IOL at or shortly before term (37 to 40 weeks) *versus* expectant management | ***Caesarean section:***  RR 0.91 [0.76 to 1.09], 4 trials, n=1190  ***Spontaneous vaginal birth:***  RR 1.09 [0.99 to 1.20], 4 trials, n=1190  ***Instrumental birth:***  RR 0.86 [0.65 to 1.13], 4 trials, n=1190  ***Adverse effects:***  *Perinatal mortality:* RR 0.00 [0.00 to 0.00], 3 trials, n=917  Serious neonatal morbidity or mortality (composite outcome): **RR 0.32 [0.15 to 0.71], 1 trial, n=818** | High  Score = 15  1 non-critical flaw on 10 |
| Bricker 2000 | To determine the effects of amniotomy alone for third trimester IOL in women with a live fetus. | Pregnant women and their babies. | Amniotomy alone for third trimester cervical ripening *or* IOL versus Placebo/no treatment | ***Caesarean section:***  RR 9.0 [0.55 to 147.95], 1 trial, n=20 | Moderate  Score = 10  3 non-critical on 5, 6, 10; no meta-analysis |
| Bricker 2015 | To assess the effects on obstetric practice and pregnancy outcome of routine late pregnancy ultrasound, defined as greater than 24 weeks gestation, in women with either unselected or low-risk pregnancies. | Pregnant women (either unselected or low-risk pregnancies) and their babies. | Routine late pregnancy ultrasound (> 24 weeks gestation) *versus* no, concealed or selective ultrasound > 24 weeks | ***Caesarean section:***  RR 1.03 [0.92 to 1.15], 6 trials, n=27461  *Elective CS:* RR 1.09 [0.89 to 1.34], 4 trials, n=5884 *Emergency CS:* RR 1.03 [0.89 to 1.20], 5 trials, n=12310  ***Instrumental birth:***  RR 1.05 [0.95 to 1.16], 5 trials, n=12310  ***Adverse effects:***  *Perinatal mortality:* RR 1.01 [0.67 to 1.54], 8 trials, n=30675  *Stillbirths (non-prespecified):* RR 1.18 [0.51 to 2.70], 6 trials, n=28107  *Stillbirths (excluding congenital abnormalities):* RR 0.05 [0.00 to 0.90], 2 trials, n=2902  *Neonatal deaths:* RR 1.04 [0.58 to 1.85], 5 trials, n=21708 | High  Score = 15  1 non-critical flaw on 10 |
| Brown 2013 | To determine whether active management of labour reduces CS rates in low-risk women and improves satisfaction. | Nulliparous women in labour and their babies. | Active management (described) of labour package *versus* usual care | ***Caesarean section:***  RR 0.88 [0.77 to 1.01], 7 trials, n=5390  *Sensitivity analysis, 1 study excluded due to 1/3rd of women being excluded (in both intervention and control groups) before the onset of labour:* **RR 0.77 [0.63 to 0.94], 6 trials, n=3475**  ***Assisted vaginal birth:***  RR 0.99 [0.87 to 1.14], 6 trials, n=3475  ***Satisfaction:***  *With care:* RR 1.04 [0.94 to 1.15], 1 trial, n=468 | Low  Score = 14  1 critical flaw on 15; 1 non-critical on 10 |
| Brown 2017a | To evaluate the effects of combined lifestyle interventions with or without pharmacotherapy in treating women with gestational diabetes. | Pregnant women with GDM and their babies. | Lifestyle intervention *versus* Usual care (another intervention) | ***Caesarean section:***  RR 0.90 [0.78 to 1.05], 10 trials, n=3545  ***Adverse effects:***  *Stillbirth:* RR 0.15 [0.01 to 2.86], 4 trials, n=2355 *Neonatal death:* RR 0.73 [0.22 to 2.42], 5 trials, n=3055  *Perinatal (fetal and neonatal mortality) and later infant mortality:* RR 0.09 [0.01 to 1.70], 2 trials, n=1988 | High  Score = 16  No flaws |
| Brown 2017b | To evaluate the effects of insulin in treating women with gestational diabetes. | Pregnant women with GDM and their babies. | Insulin *versus* Diet /standard care (3 trials, n=369) *or* Usual antenatal care | ***Caesarean section:***  RR 0.85 [0.50 to 1.42], 2 trials, n=133  ***Adverse effects:***  *Perinatal (fetal and neonatal mortality) and later infant mortality:* RR 0.74 [0.41 to 1.33], 4 trials, n=1137  *Neonatal mortality:* RR 0.72 [0.23 to 2.23], 1 trial, n=611 | High  Score = 16  No flaws |
| Bugg 2013 | To determine if the use of oxytocin for the treatment of slow progress in the first stage of spontaneous labour is associated with a reduction in the incidence of CS or maternal and fetal morbidity compared to situations where it is not used or where its administration is delayed. | Low-risk women (37-42 weeks) in labour and their babies. | Oxytocin for the treatment of slow progress in the first stage of spontaneous labour *versus* Placebo *or* no treatment (or delayed oxytocin) | ***Caesarean section:***  RR 0.84 [0.36 to 1.96], 3 trials, n=138  ***Instrumental birth:***  RR 1.04 [0.45 to 2.41], 3 trials, n=138  ***Spontaneous Vaginal Birth:***  RR 1.02 [0.84 to 1.25], 3 trials, n=138 | High  Score = 15  1 non-critical on 10 |
| Catling 2015 | To compare the effects of group antenatal care versus conventional antenatal care on psychosocial, physiological, labour and birth outcomes for women and their babies (and on care provider satisfaction). | Pregnant women accessing antenatal care. |  | ***Caesarean section:***  RR 0.83 [0.68 to 1.02], 2 trials, n=842  ***Spontaneous vaginal birth:***  RR 0.96 [0.80 to 1.15], 1 trial, n=322  ***Operative vaginal birth:***  RR 1.83 [0.75 to 4.48], 1 trial, n=322  ***Adverse effects:***  *Perinatal mortality:* RR 0.63 [0.32 to 1.25], 3 trials, n=1943  ***Satisfaction:***  *With antenatal care*: RR 4.90 [3.10 to 6.70], 1 trial, n=993 | High  Score = 15  1 non-critical on 10 |
| Ceysens 2006 | To evaluate the effect of exercise programs, alone or in conjunction with other therapies, compared to no specific program or to other therapies, in pregnant women with diabetes on perinatal and maternal morbidity and on the frequency of prescription of insulin to control glycaemia. | Pregnant women with gestational diabetes and their babies | Exercise programs, alone or in conjunction with other therapies *versus* no specific programs | ***Caesarean section:***  *Exercise + diet vs diet alone*: RR 0.93 [0.22 to 3.88], 1 trial, n=29  ***Adverse effects:***  *Stillbirth:* RR 0.00 [0.00 to 0.00], 2 trials, n=48 | Low  Score = 13  1 critical flaws on 15, 2 non-critical on 5, 10 |
| Cluett & Burns 2009 | To assess the evidence from randomised controlled trials about immersion in water during labour and water birth on maternal, fetal, neonatal and caregiver outcomes. | Pregnant women, singleton, in labour and their babies. | Immersion in water 1^st^ or 2^nd^ stage *versus* no immersion | ***Caesarean section:***  *Immersion in 1^st^ stage:* RR 1.21 [0.87 to 1.68], 8 trials, n=2712  *Immersion in 2^nd^ stage*: RR 0.33 [0.07 to 1.52], 2 trials, n=180  ***Assisted vaginal birth:***  *Immersion in 1^st^ stage:* RR 0.86 [0.71 to 1.05], 7 trials, n=2628  *Immersion in 2^nd^ stage:* RR 0.73 [0.21 to 2.54], 2 trials, n=180  ***Spontaneous vs operative vaginal birth:***  Immersion in 1^st^ stage: **RR 1.26 [1.09 to 1.45], 1 trial, n=106**  ***Adverse effects:***  *Immersion in 2^nd^ stage - Perinatal mortality:* RR 3.0 [0.12 to 72.20], 1 trial, n=120  ***Satisfaction:***  *Immersion in 2^nd^ stage- Satisfaction with labour and birth on scale of 0-6 where 0 is not at all satisfied:* RR 0.03 [-0.64 to 0.70], 1 trial, n=60 | Low  Score = 12  1 critical flaw on 15; 1 non-critical on 10 |
| Cluver 2015 | To assess the effects of interventions such as tocolysis, acoustic stimulation for midline spine position, regional analgesia (epidural or spinal), transabdominal amnio-infusion, systemic opioids and hypnosis, or the use of abdominal lubricants, on ECV at term for successful version, presentation at birth, method of birth and perinatal and maternal morbidity and mortality. | Women with singleton breech presentations at term and no contraindications to ECV. | Interventions to facilitate ECV *versus* placebo | ***Caesarean section:***  Beta stimulants: **RR 0.77 [0.67 to 0.88], 6 trials, n=742**  *Calcium channel blockers:* RR 1.11 [0.88 to 1.40], 1 trial, n=310  *Nitric oxide donors:* RR 0.83 [0.67 to 1.02], 1 trial, n=125  *Systemic opioids:* RR 0.99 [0.63 to 1.57], 1 trial, n=60  Tocolytics Nulliparae: **RR 0.85 [0.75 to 0.97], 2 trials, n=170**  *Tocolytics Multiparae:* RR 0.67 [0.38 to 1.17], 2 trials, n=79  ***Operative vaginal birth:***  *Calcium channel blockers:* RR 0.34 [0.09 to 1.22], 1 trial, n=310  *Systemic opioids:* RR 0.94 [0.20 to 4.27], 1 trial, n=60  ***Adverse effects:***  *Calcium channel blockers - Perinatal death:* RR 0.00 [0.00 to 0.00], 1 trial, n=310  ***Satisfaction:***  *Systemic opioids:* **RR 2.60 [1.25 to 3.95], 1 trial, n=60** | High  Score = 15  1 non-critical flaw on 10 |
| Costley & East 2013 | To determine whether augmentation of women using epidural analgesia with oxytocin will decrease the incidence of operative deliveries and thereby reduce fetal and maternal morbidity. | All women in SOL with epidural analgesia. | Augmentation with oxytocin *versus* placebo (saline) | ***Caesarean section:***  RR 0.95 [0.42 to 2.12], 2 trials, n=319  *Cervical dilatation < 10cm:* RR 1.17 [0.46 to 2.96], 1 trial, n=93  *Cervical dilatation 10cm:* RR 0.55 [0.10 to 2.92], 1 trial, n=226  ***Instrumental birth:***  RR 0.88 [0.72 to 1.08], 2 trials, n=319  *Cervical dilatation < 10 cm:* RR 0.95 [0.67 to 1.34], 1 trial, n=93  *Cervical dilatation 10 cm:* RR 0.85 [0.66 to 1.09], 1 trial, n=226 | High  Score = 15  1 non-critical flaw on 10 |
| Dawood 2013 | To evaluate whether the routine administration of IV fluids to low-risk nulliparous labouring women reduces the duration of labour and to evaluate the safety of IV fluids on maternal and neonatal health. | Nulliparous women, terms, low risk, singleton, cephalic presentation | IV fluids *versus* no IV fluids/oral intake alone | ***Caesarean section:***  *IV + oral vs oral intake alone*: RR 0.73 [0.49 to 1.08], 2 trials, n=315 | High  Score = 15  1 non-critical flaw on 10 |
| De-Regil 2016 | To examine whether oral supplements with vitamin D alone or in combination with calcium or other vitamins and minerals given to women during pregnancy can safely improve maternal and neonatal outcomes. | Pregnant women and their babies (pregnant women with pre-existing conditions were excluded) | Vitamin D or vitamin D plus calcium *versus* no treatment/placebo | ***Caesarean section:***  *Vitamin D alone vs placebo:* RR 0.95 [0.69 to 1.31], 2 trials, n=312  ***Adverse effects:***  *Maternal mortality:* RR 0.00 [0.00 to 0.00], 1 trial, n=180  *Stillbirth:* RR 0.35 [0.06 to 1.99], 3 trials, n=540  *Neonatal mortality:* RR 0.27 [0.04 to 1.67], 2 trials, n=282 | High  Score = 16  0 flaws |
| Devane 2017 | To compare the effects of admission CTG with IA of the FHR on maternal and infant outcomes for pregnant women without risk factors on their admission to the labour ward. | Low-risk pregnant women in labour at 37 to 42 weeks gestation and their babies. | Admission CTG *versus* IA | ***Caesarean section:***  RR 1.20 [1.00 to 1.44], 4 trials, n=11338  ***Instrumental birth:***  RR 1.10 [0.95 to 1.27], 4 trials, n=11338  ***Adverse effects***:  *Fetal/neonatal mortality:* RR 1.01 [0.30 to 3.47], 4 trials, n=11339 | High  Score = 16  0 flaws |
| Dodd 2014 | To assess a policy of elective delivery from 37 weeks gestation compared with an expectant approach for women with an otherwise uncomplicated twin pregnancy. | Pregnant women with uncomplicated twin pregnancy and their babies. | Elective birth at 37 weeks gestation *versus* expectant management | ***Caesarean section:***  *Overall:* RR 1.05 [0.83 to 1.32], 2 trials, n=271  *CS for fetal distress:* RR 0.79 [0.29 to 2.15], 2 trials, n=271  ***Instrumental vaginal birth:***  RR 0.63 [0.35 to 1.15], 1 trial, n=470  ***Adverse effects:***  *Perinatal mortality or serious morbidity:* RR 0.34 [0.01 to 8.35], 2 trials, n=542  *Maternal mortality or serious morbidity:* RR 0.29 [0.06 to 1.38], 1 trial, n=235 | High  Score = 15  1 non-critical flaw on 10 |
| Dodd 2015 | To assess the value of ‘specialised’ antenatal care for women with a multiple pregnancy when compared with ‘standard’ antenatal care. | Pregnant women with a multiple pregnancy. | Antenatal care specifically designed (study defined) for women with a multiple pregnancy *versus* usual care | ***Caesarean section:***  **RR 1.38 [1.06 to 1.81], 1 trial, n=162**  ***Adverse effects:***  *Perinatal mortality:* RR 1.03 [0.26 to 4.03], 1 trial, n=324  *Stillbirth:* RR 0.68 [0.12 to 4.04], 1 trial, n=324  *Neonatal mortality:* RR 2.05 [0.19 to 22.39], 1 trial, n=324  ***Satisfaction:***  *Overall:* RR 1.28 [0.91 to 1.79], 1 trial, n=141  *With antenatal care:* RR 1.29 [0.99 to 1.67], 1 trial, n=133 | High  Score = 12  1 non-critical flaw on 10; no meta-analysis |
| Dodd 2017 | To assess the benefits and harms of progesterone administration for the prevention of preterm birth in women with a multiple pregnancy. | Pregnant women with a multiple pregnancy and their babies. | Progestogen by any route (IV, IM) oral or vaginal for the prevention of preterm birth *versus* placebo *or* no treatment | ***Caesarean section:***  *IM progesterone:* RR 1.01 [0.95 to 1.08], 7 trials, n=2222  Vaginal progesterone: **RR 0.93 [0.88 to 0.98], 6 trials, n=2143**  *IM progesterone: Multiple pregnancy and short cervix*: RR 1.14 [0.88 to 1.49], 1 trial, n=161  *Vaginal progesterone vs no treatment: multiple pregnancy and short cervix:* RR 0.99 [0.89 to 1.11], 1 trial, n=224  ***Adverse effects***:  *IM progesterone:*  *Perinatal mortality:* RR 1.45 [0.60 to 3.51], 6 trials, n=3089  *- Fetal mortality:* RR 0.93 [0.39 to 2.20], 4 trials, n=3536  *- Neonatal mortality:* RR 0.92 [0.44 to 1.91], 7 trials, n=3399  *Vaginal progesterone:*  *- Perinatal mortality:* RR 1.23 [0.74 to 2.06], 3 trials, n=2287  *-Fetal mortality:* RR 1.38 [0.65 to 2.90], 3 trials, n=2328  *- Neonatal mortality:* RR 1.53 [0.75 to 3.15], 3 trials, n=2905  ***Satisfaction:***  *Vaginal progesterone, satisfaction with therapy:* MD 0.00 [-0.35 to 0.35], 1 trial, n=494 | High  Score = 16  0 flaws |
| Dowswell 2015 | To compare the effects of antenatal care programmes with reduced visits for low-risk women with standard care. | Low-risk pregnant women and their babies, attending ANC. | Provision of a schedule of reduced number of visits, with or without goal-oriented antenatal care *versus* usual care | ***Caesarean section:***  RR 0.99 [0.91 to 1.08], 4 trials  ***Adverse effects***:  *Maternal mortality:* RR 1.13 [0.50 to 2.57], 3 trials, n=51504  *Perinatal mortality with ICC 0.0003:* RR 1.14 [1.00 to 1.31], 5 trials n=56431  *Individual:* RR 0.90 [0.45 to 1.80], 2 trials  Cluster: **RR 1.15 [1.01 to 1.32],** **3 trials**  Perinatal mortality with ICC truncated to zero: **RR 1.15 [1.02 to 1**.**30], 5 trials**  *Individual:* RR 0.90 [0.45 to 1.80], 2 trials  Cluster: **RR 1.16 [1.02 to 1.31], 3 trials**  ***Satisfaction:***  *Satisfied with amount of visit time:* MD 0.00 [-0.27 to 0.27], 1 trial, n=331  *Quality of prenatal care:* MD -0.20 [-0.28 to -0.11], 2 trials, n=2198  *Get questions answered:* MD 0.00 [0.26 to 0.26], 1 trial, n=331  *Attitude of care giver (friendliness and courtesy):* MD 0.10 [-0.14 to 0.34], 1 trial, n=331  *Would choose same schedule in future (yes):* RR 1.12 [1.05 to 1.20], 1 trial n=1862 | High  Score = 15  1 non-critical on 10 |
| Dowswell 2009a | To assess the effects of TENS on pain in labour. | Women in labour. | TENS (any model or type) *versus* placebo TENS or routine care   1. TENS to back 2. TENS to acu-points 3. Limoge to cranium | ***Caesarean section:***   1. RR 1.35 [0.84 to 2.17], 8 trials, n=868 2. RR 1.5 [0.26 to 8.60], 1 trial, n=100 3. RR 1.0 [0.07 to 13.87], 1 trial, n=20   ***Instrumental:***   1. RR 0.82 [0.56 to 1.19], 7 trials, n=840 2. RR 4.5 [1.02 to 19.79], 1 trial, n=100 3. RR 0.67 [0.14 to 3.17], 1 trial, n=20   ***Satisfactions (with pain relief):***   1. RR 1.25 [0.98 to 1.60], 5 trials, n=452 2. RR 4.1 [1.81 to 9.29], 1 trial, n=90 | High  Score = 15  1 non-critical weakness on 10 |
| Dowswell 2009b | To examine whether antenatal day care units improve outcomes for women with complicated pregnancy compared with hospital admission or routine care. | Pregnant women with complication that would have ordinarily led to hospitalisation. | Antenatal day care: admission and discharge home with no overnight stay *versus* Inpatient care *or* routine management (which includes the option of inpatient care) | ***Caesarean section:***  RR 0.53 [0.10 to 2.94], 1 trial, n=54  ***Normal vaginal birth:***  RR 0.94 [0.66 to 1.35], 1 trial, n=54  ***Instrumental birth:***  *Forceps:* RR 1.6 [0.55 to 4.68], 1 trial, n=54  ***Adverse effects:***  *Maternal mortality:* RR 0.00 [0.00 to 0.00], 2 trials, n=449  *Perinatal mortality:* RR 0.00 [0.00 to 0.00], 2 trials, n=449  ***Satisfaction:***  *Dissatisfaction with care:*  *“spent too much time in hospital”:* RR 0.35 [0.08 to 1.62], 1 trial, n=45  *“I am satisfied with the care I received” (number disagreeing or not sure):* RR 0.40 [0.18 to 0.88], 1 trial, n=350  ***Costs:***  *Public health cost: average total cost (Australian $):* MD 415.10 [-603.86 to 1434.06], 1 trial, n=395 | High  Score = 15  1 non-critical weakness on 10 |
| East 2014 | To compare the effectiveness and safety of fetal intrapartum pulse oximetry with other surveillance techniques. | Women in labour with a live baby where fetal monitoring is clinically indicated. | Use of fetal pulse oximetry with or without concurrent use of conventional fetal monitoring *versus* conventional only (no pulse oximetry)  FPO and CTG vs CTG only   1. From 34 weeks, FBS not required prior to study entry 2. From 36 weeks, FBS prior to study entry 3. From 28 weeks, FBS not required prior to study entry 4. From 36 weeks, non-reassuring fetal status not required prior to study entry | ***Caesarean section:***   1. RR 0.99 [0.86 to 1.13], 4 trials, n=4008 2. **RR 0.44 [0.24 to 0.81], 1 trial, n=146** 3. RR 0.93 [0.76 to 1.14], 1 trial, n=327 4. RR 0.96 [0.87 to 1.04], 1 trial, n=5341   ***Operative birth (CS, forceps, vacuum):***   1. RR 1.03 [0.92 to 1.15], 3 trials, n=1840 2. **RR 0.51 [0.36 to 0.73], 1 trial, n=146** 3. RR 0.96 [0.90 to 1.03], 1 trial, n=5341 4. **RR 0.74 [0.62 to 0.89], 2 trials, n=1610**   ***Adverse effects:***  *Maternal mortality:*   1. RR 0.00 [0.00 to 0.00], 2 trials, n=1789 2. RR 0.00 [0.00 to 0.00], 1 trial, n=146   *Perinatal mortality:*   1. RR 0.89 [0.20 to 3.97], 2 trials, n=1789 2. RR 0.00 [0.00 to 0.00], 1 trial, n=146 3. RR 0.34 [0.01 to 8.44], 1 trial, n=5341   ***Satisfaction:***  *With labour:* RR 0.20 [-0.16 to 0.56], 1 trial, n=448;  *With fetal monitoring in labour:* RR 0.40 [-0.05 to 0.85], 1 trial, n=448 | High  Score = 15  1 non-critical flaw on 10 |
| Falavigna 2012 | To evaluate the effectiveness of gestational diabetes (GDM) treatment compared to usual antenatal care, in the prevention of adverse pregnancy outcomes. | Pregnant women with a diagnosis of GDM according to the individual study definitions. | GDM treatment *versus* usual antenatal care | ***Caesarean section:***  RR 0.90 (0.78 to 1.05), 5 trials, n=2514  ***Adverse effects:***  *Perinatal mortality:* RR 0.62 [0.31 to 1.24], 3 trials, n=1856 and RR 0.62 [0.31–1.24], 7 trials, n=3396 | Low  Score = 14  1 critical flaw on 2 (p-yes), 1 non-critical on 10 |
| Ghosh 2016 | To determine the effects of NO donors (isosorbide mononitrate (ISMN), isosorbide dinitrate (ISDN), nitroglycerin and sodium nitroprusside) for third trimester cervical ripening or IOL, in comparison with placebo or no treatment. | Pregnant women due for third trimester IOL, carrying a viable fetus. | Nitric oxide donors *versus* placebo/no treatment | ***Caesarean section:***  *All:* RR 0.99 [0.88 to 1.11], 9 trials, n=2624  *All women, unfavourable cervix:* RR 0.97 [0.82 to 1.15], 8 trials, n=1262  *Primiparae:* RR 1.08 [0.89 to 1.31], 4 trials, n=683  ***Instrumental vaginal birth:***  RR 0.96 [0.83 to 1.10], 4 trials, n=1835  ***Adverse effects:***  *Perinatal mortality:* RR 0.33 [0.01 to 7.94], 2 trials, n=1712  *Serious neonatal morbidity/perinatal mortality:* RR 1.61 [0.08 to 33.26], 2 trials, n=1712  *Serious maternal morbidity or mortality:* RR 0.00 [0.00 to 0.00], 1 trial, n=1362  ***Satisfaction:***  *Women not satisfied:* RR 1.06 [0.82 to 1.38], 1 trial, n=1362 | High  Score = 15  1 non-critical on 10 |
| Grivell 2015 | To assess the effectiveness of antenatal CTG (both traditional and computerised assessments) in improving outcomes for mothers and their babies. | Pregnant women and their babies. | 1. Traditional antenatal CTG *versus* no AN CTG 2. Computerised antenatal CTG versus traditional antenatal CTG | ***Caesarean section:***  1. RR 1.06 [0.88 to 1.28], 3 trials, n=1279  2. RR 0.87 [0.61 to 1.24], 1 trial, n=59  ***Adverse effects:***  *Perinatal mortality:*  1. RR 2.05 [0.95 to 4.42], 4 trials, n=1627  2. **RR 0.20 [0.04 to 0.88], 2 trials, n=469** | High  Score = 15  1 non-critical on 10 |
| Gulmezoglu 2012 | To evaluate the benefits and harms of a policy of IOL at term or post-term compared with awaiting spontaneous labour or later IOL. | Pregnant women at or beyond term at low risk for complications. | IOL *versus* expectant management | ***Caesarean section:***  All: **RR 0.89 [0.81 to 0.97], 21 trials, n=8749**  *37-39 weeks:* RR 0.58 [0.30 to 1.11], 1 trial, n=716  *39-40 weeks:* RR 0.74 [0.38 to 1.41], 3 trials, n=810  *< 41 weeks:* RR 1.49 [0.90 to 2.47], 1 trial, n=231  41 weeks: **RR 0.74 [0.58 to 0.96], 4 trials, n=998**  *Cervix favourable:* RR 1.12 [0.76 to 1.65], 3 trials, n=831  Cervix unfavourable: **RR 0.88 [0.80 to 0.98], 8 trials, n=5051**  ***Instrumental vaginal birth:***  *All:* RR 1.10 [1.00 to 1.21], 12 trials, n=6227  ***Adverse effects:***  Perinatal death: **RR 0.31 [0.12 to 0.81], 17 trials, n=7407**  *Stillbirth:* RR 0.30 [0.08 to 1.08], 17 trials, n=7407 *Neonatal death:* RR 0.37 [0.10 to 1.38], 17 trials, n=7407 | High  Score = 15  1 non-critical on 10 |
| Han 2012a | To assess the effects of different types of management strategies for pregnant women with hyperglycaemia not meeting diagnostic criteria for GDM and T2DM. | Pregnant women with hyperglycaemia and their babies. | Any form of management for women with pregnancy hyperglycaemia not meeting GDM diagnostic criteria *versus* routine care | ***Caesarean section:***  RR 0.93 [0.68 to 1.27], 3 trials, n=509  *Primary CS:* RR 0.99 [0.42 to 2.33], 2 trials, n=209 *Repeat CS:* RR 0.46 [0.17 to 1.26], 2 trials, n=209  ***Operative vaginal birth:***  *Unspecified:* RR 1.37 [0.20 to 9.27], 1 trial, n=83  *Vacuum only:* RR 2.74 [0.26 to 29.07], 1 trial, n=83 *Forceps:* RR 0.45 [0.02 to 10.82], 1 trial, n=83 | High  Score = 15  1 non-critical on 10 |
| Han 2012b | To assess the effects of physical exercise for pregnant women for preventing glucose intolerance or GDM. | Pregnant women excluding women with pre-existing type 1 and type 2 diabetes. | Any exercise intervention *versus* usual care | ***Caesareans section:***  RR 1.33 [0.97 to 1.84], 2 trials, n=934  ***Instrumental birth:***  RR 0.83 [0.58 to 1.17], 2 trials, n=934  ***Adherence:***  ‘Excellent in 4/5 included trials’ | High  Score = 15  1 non-critical on 10 |
| Hapangama 2009 | To determine the effects of mifepristone for third trimester cervical ripening or IOL. | Pregnant women due for third trimester IOL carrying a viable fetus. | Mifepristone *versus* placebo/no treatment | ***Caesarean section:***  Mifepristone all doses, all women: **RR 0.74 [0.60 to 0.92], 9 trials, n=1043**  CS for unsuccessful IOL: **RR 0.43 [0.23 to 0.81], 5 trials, n=759**  *CS for CTG abnormalities:* RR 1.35 [0.85 to 2.13], 7 trials, n=844  All women, unfavourable cervix: **RR 0.77 [0.61 to 0.96], 8 trials, n=919**  *Primiparae:* RR 0.71 [0.47 to 1.06], 3 trials, n=254  *Mifepristone single dose (50 mg):* RR 0.64 [0.39 to 1.04], 2 trials, n=171  *Mifepristone single dose (200 mg):* RR 0.88 [0.59 to 1.30], 3 trials, n=352  *Mifepristone single dose (400 mg):* RR 0.94 [0.67 to 1.31], 4 trials, n=282  *Mifepristone single dose (600 mg):* RR 0.75 [0.44 to 1.25], 1 trial, n=116  *Mifepristone single dose (100 mg):* RR 0.80 [0.48 to 1.34], 1 trial, n=112  ***Instrumental vaginal birth:***  **RR 1.43 [1.04 to 1.96], 7 trials, n=814**  ***Adverse effects:***  *Perinatal mortality:* RR 0.00 [0.00 to 0.00], 7 trials, n=869  Maternal adverse effects (all): **RR 1.51 [1.06 to 2.15], 4 trials, n=734** | High  Score = 15  1 non-critical on 10 |
| Heazell 2015 | To assess whether clinicians’ knowledge of the results of biochemical tests of placental function is associated with improvement in fetal or maternal outcome of pregnancy. | Pregnant women in labour and their babies. | Tests of placental function *versus* standard care | ***Caesarean section:***  RR 0.48 [0.15 to 1.52], 1 trial, n=118  ***Adverse effects:***  *Stillbirth:* RR 0.56 [0.16 to 1.88], 2 trials, n=740 *Neonatal mortality:* RR 1.62 [0.39 to 6.74], 2 trials, n=740 | High  Score = 15  1 non-critical on 10 |
| Hodnett 2010 | To assess effects of programs offering additional social support compared with routine care, for pregnant women believed at high risk for giving birth to babies that are either preterm or weigh less than 2500 gm, or both, at birth. | Pregnant women judged to be at risk of having preterm or growth-restricted babies, or both. | Standardized or individualized programs of additional social support *versus* usual care | ***Caesarean section:***  **RR 0.87 [0.78 to 0.97], 9 trials, n=4522**  ***Adverse effects:***  *Stillbirth/neonatal mortality:* RR 0.96 [0.74 to 1.26], 11 trials, n=7522  ***Satisfaction:***  *Less than very satisfied with antenatal care:* RR 1.13 [0.76 to 1.67], 1 trial, n=1887 | Low  Score = 14  1 critical flaw on 15; 1 non-critical flaw on 10 |
| Hodnett 2012 | To assess the effects of care in an alternative institutional birth environment compared to care in a conventional setting. | Pregnant women at low risk of obstetric complications. | Alternative institutional birth setting *versus* conventional hospital setting | ***Caesarean section:***  **RR 0.88 [0.78 to 1.00], 9 trials, n=11350**  ***Spontaneous vaginal birth:***  **RR 1.03 [1.01 to 1.05], 8 trials, n=11202**  ***Instrumental vaginal birth:***  **RR 0.89 [0.79 to 0.99], 8 trials, n=11202**  ***Adverse effects:***  *Perinatal mortality:* RR 1.67 [0.93 to 3.00], 8 trials, n=11206  *Serious perinatal morbidity or mortality:* RR 1.17 [0.51 to 2.67], 5 trials, n=6385  *Serious maternal morbidity or mortality:* RR 1.11 [0.23 to 5.36], 4 trials, n=6334  ***Satisfaction:***  Very positive views of care*:* **RR 1.96 [1.78 to 2.15], 2 trials, n=1207** | Low  Score = 14  1 critical flaw on 15; 1 non-critical flaw on 10 |
| Hofmeyr 2010 | To determine the effects of vaginal misoprostol for third trimester cervical ripening or IOL. | Pregnant women due for third trimester IOL (multiple pregnancies are included). | Vaginal misoprostol *versus* placebo/no treatment | ***Caesarean section:***  RR 0.81 [0.63 to 1.05], 10 trials, n=1141  *All women, unfavourable cervix:* RR 0.95 [0.69 to 1.30], 7 trials, n=862  *Primiparae:* RR 1.09 [0.49 to 2.41], 1 trial, n=39  ***Instrumental vaginal birth:***  RR 1.07 [0.65 to 1.77], 3 trials, n=184  ***Adverse effects:***  *Perinatal mortality:* RR 0.34 [0.01 to 8.14], 2 trials, n=122  *Maternal mortality:* RR 0.00 [0.00 to 0.00], 1 trial, n=45  *Serious maternal complication:* RR 0.68 [0.12 to 3.87], 3 trials, n=272 | Low  Score = 12  1 critical on 13, 3 non-critical on 5, 6, 10 |
| Hofmeyr 2012a | To assess the effects of piracetam for suspected fetal distress in labour on method of delivery and perinatal morbidity. | Women with suspected fetal distress in labour. | Piracetam for suspected fetal distress *versus* placebo | ***Caesarean section:***  RR 0.57 [0.32 to 1.03], 1 trial, n=96 | High  Score = 12  1 non-critical on 10; no meta-analysis |
| Hofmeyr 2012b | To assess the effects of amnioinfusion for potential or suspected umbilical cord compression on maternal and perinatal outcome. | Women whose babies were considered to be at increased risk of, or had FHR patterns suggestive of, umbilical cord compression in labour. | Amnioinfusion (AI) *versus* no AI | ***Caesarean section:***  *Transcervical AI* CS overall: **RR 0.62 [0.46 to 0.83], 13 trials, n=1493**  CS for suspected fetal distress: **RR 0.46 [0.31 to 0.68], 12 trials, n=1588**  *Transabdominal amnioinfusion CS overall:* RR 0.43 [0.17 to 1.10], 1 trial, n=79  CS for suspected fetal distress: **RR 0.20 [0.05 to 0.74], 2 trials, n=110** | High  Score = 15  1 non-critical on 10 |
| Hofmeyr 2014 | To assess the effects of amnioinfusion for meconium-stained liquor on perinatal outcome. | Women in labour with moderate or thick meconium staining of the amniotic fluid. | Amnioinfusion for meconium-stained liquor in labour *versus* no amnioinfusion | ***Caesarean section:***  *Standard peripartum surveillance:* RR 0.78 [0. 60 to 1.02], 11 trials, n=3380  **Limited peripartum surveillance: RR 0.59 [0.41 to 0.84], 3 trials, n=1137**  ***Instrumental vaginal birth:***  **RR 0.68 [0.50 to 0.91], 9 trials, n=2059**  ***Adverse effects:***  Perinatal mortality: **RR 0.35 [0.18 to 0.66], 10 trials, n=3913**  *Maternal mortality or serious morbidity (post hoc) standard peripartum surveillance:* RR 1.00 [0.49 to 2.04], 1 trial, n=1975 | High  Score = 15  1 non-critical on 10 |
| Hofmeyr 2015 | To assess the effects of ECV at or near term on measures of pregnancy outcome. | Pregnant women with babies in the breech presentation at or near term. | ECV *versus* no ECV | ***Caesarean section:***  **RR 0.57 [0.40 to 0.82], 8 trials, n=1305**  High quality trials: **RR 0.46 [0.27 to 0.79], 5 trials, n=428**  ***Adverse effects:***  *Perinatal mortality:* RR 0.39 [0.09 to 1.64], 8 trials, n=1305 | High  Score = 15  1 non-critical on 10 |
| Hofmeyr 2017 | To determine if fundal pressure is effective in achieving SVD and preventing prolonged second stage or the need for operative birth, and to explore maternal and neonatal adverse effects related to fundal pressure. | Women in the second stage of labour with singleton, cephalic presentation. | Manual fundal pressure or by inflatable belt *versus* no fundal pressure | ***Caesarean section:***  *Manual fundal pressure:* RR 1.10 [0.07 to 17.27], 1 trial, n=197  *Fundal pressure by inflatable belt:* RR 0.56 [0.14 to 2.26], 4 trials, n=891  ***Instrumental birth:***  *Manual fundal pressure:* RR 3.28 [0.14 to 79.65], 1 trial, n=197  *Fundal pressure by inflatable belt:* RR 0.73 [0.52 to 1.02], 4 trials, n=891  ***Adverse effects:***  *Neonatal mortality:* RR 0.00 [0.00 to 0.00], 2 trials, n=2445 | High  Score = 15  1 non-critical on 10 |
| Horey 2004 | To examine the effectiveness of information about caesarean birth. | Pregnant women and their babies. | Any intervention designed to inform women about CS *versus* usual care | ***Caesarean section:***  RR 0.90 [0.65 to 1.24], 1 trial, n=176  RR 0.92 [0.82 to 1.03], 1 trial, n=1275 | High  Score = 12  1 non-critical on 10; no meta-analysis |
| Horey 2013 | To examine the effectiveness of interventions to support decision-making about vaginal birth after a caesarean birth. | Pregnant women who previously have had a CS. | Interventions designed specifically to support pregnant women who have previously had a CS make decisions about their options for birth *versus* usual care/no formal decision support | ***Caesarean section:***  RR 0.96 [0.84 to 1.10], 3 trials, n=2071  ***Vaginal birth:***  *SVD:* RR 0.97 [0.89 to 1.06], 3 trials, n=2190  *VBAC:* RR 1.03 [0.97 to 1.10], 3 trials, n=2071  ***Adverse effects:***  *Perinatal mortality:* RR 0.50 [0.09 to 2.69], 1 trial, n=1280  ***Satisfaction:***  *With decision-making process:* MD 0.06 [-0.09 to 0.20], 2 trials, n=797 | High  Score = 16  No weaknesses |
| Hutton 2015 | To assess the effectiveness of a policy of beginning external cephalic version (ECV) before term for breech presentation on fetal presentation at birth, method of delivery, and the rate of preterm birth, perinatal morbidity, stillbirth and neonatal mortality. | Women with a live singleton fetus in breech presentation before term. | External cephalic version attempt before term (37 weeks’ gestation) *versus* no ECV or ECV at term | ***Caesarean section:***  *ECV before term*: RR 1.82 [0.57 to 5.84], 1 trial, n=102  ***Vaginal breech birth:***  *ECV before term:* RR 0.87 [0.49 to 1.52], 1 trial, n=102  ***Adverse effects:***  *Perinatal mortality ECV before term:* RR 0.35 [0.04 to 3.22], 1 trial, n=102  *Stillbirth and neonatal mortality < 7 days:* RR 0.34 [0.01 to 8.16], 1 trial, n=179 | High  Score = 15  1 non-critical on 10 |
| Jozwiak 2012 | To determine the effects of mechanical methods for third trimester cervical ripening or IOL in comparison with placebo/no treatment, prostaglandins (vaginal and intracervical prostaglandin E2 (PGE2), misoprostol) and oxytocin. | Pregnant women due for third trimester IOL, carrying a viable fetus | Any mechanical method (all women) *versus* placebo | ***Caesarean section:***  *Any method:* RR 1.00 [0.76 to 1.30], 6 trials, n=416 *Balloon catheter:* RR 1.17 [0.47 to 2.92], 1 trial, n=44 | High  Score = 15  1 non-critical on 10 |
| Kavanagh 2005 | To determine the effectiveness of breast stimulation for third trimester cervical ripening or IOL in comparison with placebo/no intervention or other methods of IOL. | Pregnant women due for third trimester IOL, carrying a viable fetus. | Breast stimulation either by self-stimulation through massage or by a mechanical method *versus* no intervention | ***Caesarean section:***  RR 0.90 [0.38 to 2.12], 1 trial, n=200  ***Adverse effects:***  *Perinatal mortality:* RR 8.17 [0.45 to 147.76], 3 trials, n=337 | Low  Score = 11  1 critical on 15; 1 non-critical on 10 |
| Kavanagh 2006 | To determine the effects of hyaluronidase for third trimester cervical ripening or IOL in comparison with other methods of IOL. | Pregnant women due for third trimester IOL, carrying a viable fetus. | Hyaluronidase *versus* placebo/no treatment | ***Caesarean section:***  All women: **RR 0.37 [0.22 to 0.61], 1 trial, n=168**  Primiparae: **RR 0.43 [0.23 to 0.81], 1 trial, n=94**  Multiparae: **RR 0.28 [0.12 to 0.67], 1 trial , n=74**  All women with previous CS: **RR 0.35 [0.15 to 0.81], 1 trial, n=29** | Moderate  Score = 10  3 non-critical on 8 (p-yes), 10, 14; no meta-analysis |
| Kelly 2001 | To determine the effects of relaxin (both purified porcine and recombinant human) for third trimester cervical ripening or IOL in comparison with other methods of IOL. | Pregnant women due for third trimester IOL, carrying a viable fetus. | Relaxin (both purified porcine and recombinant human) *versus* placebo | ***Caesarean section:***  *All women:* RR 0.79 [0.42 to 1.50], 4 trials, n=267  *Unfavourable cervix:* RR 0.88 [0.45 to 1.73], 3 trials, n=207  *Instrumental birth:* RR 1.19 [0.76 to 1.86], 3 trials, n=196  ***Adverse effects:***  *Perinatal mortality:* RR 0.00 [0.00 to 0.00], 2 trials, n=136 | Low  Score = 15  1 critical flaw on 15; 2 non-critical on 6, 10 |
| Kelly 2013 | To determine the effects of castor oil or enemas for third trimester cervical ripening or IOL in comparison with other methods of cervical ripening or IOL. | Pregnant women due for third trimester IOL, carrying a viable fetus. | Castor oil or enemas for cervical ripening or IOL *versus* placebo/no treatment | ***Caesarean section:***  *All women:* RR 2.04 [0.92 to 4.55], 2 trials, n=180  *All women, unfavourable cervix:* RR 2.04 [0.92 to 4.55], 2 trials, n=180  ***Instrumental vaginal birth:***  *All women:* RR 0.46 [0.10 to 2.26], 1 trial, n=80  ***Adverse effects:***  *Serious maternal complications:* RR 0.17 [0.01 to 3.10], 1 trial, n=80 | Moderate  Score = 13  3 non-critical weaknesses on 8, 10, 14 |
| Kobayashi 2017 | To investigate the effect of assessment and support interventions for women during early labour on the duration of labour, the rate of obstetric interventions, and on other maternal and neonatal outcomes. | Pregnant women (high risk excluded). | Assessment programmes in early labour *versus* no intervention or usual care. | ***Caesarean section:***  *Assessment vs Direct Admission:* RR 0.72 [0.30 to 1.72], 1 trial, n=209  *Home vs Telephone triage:* RR 1.05 [0.95 to 1.17], 3 trials, n=5170  *1-1 structured care vs usual care:* RR 0.93 [0.84 to 1.02], 1 study, n=4996  *1-1 structured care in early labour vs usual care:* RR 0.93 [0.84 to 1.02], 1 study, n=4996  ***Instrumental vaginal birth:***  *Home assessment vs Telephone triage:* RR 0.95 [0.79 to 1.15], 2 trials, n=4933  *1-1 structured care in early labour:* RR 0.94 [0.82 to 1.08], 1 study, n=4996  ***Adverse effects:***  *Home assessment vs Telephone triage:*  *- Serious maternal morbidity (PPH > 1L, postnatal fever, blood transfusion and maternal mortality):* RR 0.93 [0.61 to 1.42], 1 trial, n=3474  *- Perinatal mortality:* RR 1.00 [0.42 to 2.40], 1 trial, n=3474 infants  *1-1 structured care - Serious maternal morbidity*: RR 1.13 [0.84 to 1.52], 1 study, n=4996  ***Maternal satisfaction:***  *Assessment vs Direct admission:* MD 16.00 [7.53 to 24.47], 1 study, n=201;  *Home assessment vs Telephone triage:* MD 3.47 [1.00 to 5.94], 1 trial, n=423 | High  Score = 15  1 non-critical weakness on 14 |
| Khunpradit 2011a | To assess the effectiveness of admission tests other than cardiotocography in preventing adverse perinatal outcomes. | Pregnant women at admission to labour room. | Labour admission tests including vibroacoustic stimulation, biophysical profile (BPP), modified BPP, rapid BPP, Doppler scans  of the umbilical artery, and sonographic assessment of amniotic fluid *versus* no sonographic measurement | ***Caesarean section for fetal distress:***  RR 2.02 [1.08 to 3.77], 1 study, n=883 | High  Score = 13  No meta-analysis conducted |
| Khunpradit 2011b | To determine the effectiveness and safety of non-clinical interventions for reducing unnecessary caesarean section rates. | Pregnant women and their families, healthcare providers who work with expectant mothers, communities and advocacy groups. | Patient directed interventions;  professional, including education, audit and feedback, practice guidelines; organisational, e.g. quality improvement strategies; Financial, e.g. incentives for certain procedures; and  Regulatory, e.g. mandatory second opinions *versus* usual care | ***Caesarean section:***  *Information programme:* OR 0.93 [0.61 to 1.41] *Decision analysis:* OR 1.42 [0.94 to 2.14]  *Peer review and feedback change in level:*   - 1 month -3.7% [-6.4% to -1.1%] - 6 months -4.4% [-6.9% to -1.8%] - 12 months -5.1% [-7.8% to -2.4%]   *Intensive group therapy:* Absolute difference: -4.9% *Decision aid booklet- Elective repeat caesarean section:*   - Absolute difference post intervention: 2.8% - Absolute change from baseline: 22.3% versus 26.2%   *Legislation based practice and guideline- Total caesarean section rate:*   - *3 months change in level:* -0.001% [-0.8% to 0.8%] - *6 months:* -0.14% [-0.8% to 0.6%]   ***Instrumental vaginal birth:***  *Relaxation education:* 21.2% vs 48.1%, P = 0.002, RD: -26.9%  ***Fear of Childbirth:***  *Intensive group therapy:*  Fear of pain in labour decreased significantly (4.7 (SD = 0.6) to 4.4 (SD = 1.0) versus (4.4 (SD = 0.9) to 4.5 (SD = 1.1), P = 0.041).  Fear of obstetrician’s unfriendly behaviour decreased (2.9 (SD) = 1.4 to 2.6 (SD) = 1.2) versus (2.8 (SD) = 1.3 to 2.9 (SD) = 1.4, P = 0.054).  ***Adverse effects:***  *Evidence based guideline with mandatory 2nd opinion:*  *- Neonatal mortality:* Absolute difference: -0.1%  - *Perinatal mortality:* Absolute difference: -0.5 Relative % difference: -17.2%, Absolute change from baseline Adj RD: -0.4; Relative rate reduction: 11.8  *Prenatal education and support* - *Perinatal deaths:* Intervention: 2/643, Control: 4/637  *Peer review and feedback on use of guidelines* :  - *Perinatal mortality:* Pre: Int: 77/6862 (1.12%), Post: Int: 52/6050 (0.86%) Relative % difference: -32.14%; -*Neonatal mortality:* Pre: Int: 25/6862 (0.36%), Post: Int: 14/6050 (0.23%) Risk difference: -0.13%, Relative % difference: -36.1%  -*Fetal mortality:* Pre: Int: 51/6862 (0.72%), Post: Int: 38/6050 (0.63%), Relative % difference: -12.5%  ***Satisfaction:***  *Information programme - Satisfaction with decision:* Adj diff 0.06 [-0.06 to 0.19], P = 0.31  *Decision analysis versus usual care - Satisfaction with decision:* Adj diff 0.14 [0.02 to 0.27], P = 0.022  - *Decision-aid booklet - Satisfaction scores (out of 10):* Int: 7.70 Con: 7.90 | High  Score = 12  1 non-critical on 10; no meta-analysis |
| Lalor 2008 | To assess the effects of the BPP when compared with conventional monitoring (CTG only or MBPP) on pregnancy outcome in high-risk pregnancies. | Pregnant women with singleton, high-risk pregnancies at greater than 24 completed weeks gestation not in labour, and their babies. | Biophysical profile *versus* conventional monitoring | ***Caesarean section:***  RR 1.18 [0.90 to 1.54], 4 trials, n=2239  *CS for fetal distress:* RR 1.18 [0.83 to 1.68], 2 trials, n=1452  ***Adverse effects:***  *Perinatal mortality including major malformations:* RR 1.33 [0.60 to 2.98], 4 trials, n=2839 | Moderate  Score = 12  2 non-critical weaknesses |
| Lauzon 2001 | To assess the effects of labour assessment programs that aim to delay hospital admission until labour is in the active phase. | All pregnant women at term gestation. | Any hospital or community-based programs that aim to delay hospital or labour ward admission until active labour *versus* Direct admission | ***Caesarean section:***  RR 0.70 [0.27 to 1.79], 1 trial, n=209  ***Forceps/Vacuum:***  RR 0.79 [0.45 to 1.41], 1 trial, n=209 | High  Score = 13  1 non-critical; no meta-analysis |
| Lavender 2013 | To determine the effect of use of partogram on perinatal and maternal morbidity and mortality. | Women with singleton pregnancies, cephalic in spontaneous labour at term. | Labour management using a partogram *versus* no partogram used | ***Caesarean section:***  *Overall:* RR 0.64 [0.24 to 1.70], 2 trials, n=1590  Low-resource setting: **RR 0.38 [0.24 to 0.61], 1 trial, n=434**  *High-resource setting:* RR 1.03 [0.82 to 1.28], 1 trial, n=1156  ***Instrumental vaginal delivery:***  RR 1.00 [0.85 to 1.17], 2 trials, n=1590 | High  Score = 15  1 non-critical weakness on 10 |
| Lundgren 2015 | To evaluate clinician-centred interventions designed to increase the rate of VBAC in women with a previous CS birth(s). | Women with a previous CS. | Any clinician-centred intervention designed to increase the rate of VBAC *versus* no intervention | ***VBAC***:  X-ray Pelvimetry as an assessment for suitability for VBAC: **RR 0.38 [0.25 to 0.58], 1 study, n=288**  Opinion leaders: **RR 1.74 [1.45 to 2.09], 1 study, n=1972**  *Audit and feedback of CS:* RR 0.82 [0.62 to 1.07], 1 study, n=1757  *Peer review:* MD -1.90 [-5.39 to 1.59], 1 study, n=165 | Low |
| Lutomski 2015 | To evaluate the effectiveness of continuous or intermittent CTG  monitoring during labour with an ES. | Pregnant women in labour and their babies. | Continuous CTG monitoring with an ES or Intermittent CTG monitoring with an ES *versus* monitoring with no ES | ***Caesarean section:***  *CTG with ES vs CTG with FBS*: RR 0.61 [0.35 to 1.04], 1 trial, n=220  ***Forceps-assisted vaginal birth:***  RR 0.50 [0.05 to 5.43], 1 trial, n=220  ***Adverse effects:***  *Fetal mortality:* 0, 1 trial, n=220 | High  Score = 12  1 non-critical weakness; no meta-analysis conducted |
| Madden 2016 | To examine the effectiveness and safety of hypnosis for pain management during labour and childbirth. | Pregnant women. | Preparation for labour using hypnosis and/or use of hypnosis during labour, with or without concurrent use of pharmacological or  non-pharmacological pain-relief methods *versus* placebo/no treatment/standard care | ***Caesarean section:***  *Self-hypnosis or hypnotherapy vs standard care:* RR 1.17 [0.97 to 1.42], 3 trials, n=1700  *Nurse/CD hypnosis vs standard care:* RR 0.91 [0.56 to 1.48], 1 trial, n=294.  ***Assisted vaginal birth:***  *Self-hypnosis or hypnotherapy vs standard care:* RR 0.88 [0.72 to 1.06], 4 trials, n=1765  *Nurse/CD hypnosis versus standard care:* RR 1.20 [0.78 to 1.85], 1 trial, n=294  ***Fear of Childbirth:***  *Wijmas score at 6 weeks:* PP MD -4.60 [-8.34 to -0.86], 1 trial, n=678  ***Adverse effects:***  *Stillbirth:* RR 2.91 [0.30 to 27.79], 1 trial, n=522  ***Satisfaction:***  *Self-hypnosis or hypnotherapy versus standard care*: RR 1.36 [0.52 to 3.59], 2 trials, n=370  *Nurse/CD hypnosis vs standard care:* RR 0.94 [0.83 to 1.07], 1 trial, n=294  ***Cost:***  *Self-hypnosis or hypnotherapy vs standard care:* MD 4.83 [-257.78 to 267.44], 1 trial, n=672 | Low  Score = 15  1 non-critical weakness on 10 |
| Madhuvrata 2015 | To see if there was any intervention that could be used for primary prevention of gestational diabetes mellitus in women with risk factors for gestational diabetes mellitus. | Women with risk factors for GDM. | Diet (4 trials), exercise (3 trials), lifestyle changes (5 trials) and metformin (5 trials) *versus* standard care | ***Caesarean section:***  *Diet:* OR 1.36 [0.77 to 2.41], 2 trials, n=282 *Exercise:* OR 0.98 [0.32 to 3.00], 1 trial, n=85 *Lifestyle changes:* OR 0.90 [0.52 to 1.55], 4 trials, n=605  *Metformin:* OR 1.15 [0.63 to 2.08], 1 trial, n=270 | Moderate  Score = 14  1 critical partial yes on 4; 1 non-critical on 10 |
| Malin  2016 | To assess the effect of oral carbohydrate supplementation on labour outcomes. | Women in labour (<6 cm dilated). | Oral carbohydrate supplementation introduced in the latent or early active stage of labour *versus* placebo or standard care | ***Caesarean section:***  RR 1.15 [0.83 to 1.61], 4 trials, n= 698  ***Instrumental birth rate vs SVD:***  RR 1.26 [0.96 to 1.65], 4 trials, n=649 | Low  Score = 12  1 critical weakness on 7, 3 non-critical on 3, 10, 16 |
| Mangesi 2015 | To assess outcomes of pregnancy where fetal movement counting was done routinely, selectively or was not done at all; and to compare different methods of fetal movement counting. | Pregnant women who had reached the gestational age of fetal viability. | Routine or selective FM counting *versus* no counting, alternative method of counting | ***Caesarean section:***  *FMc vs undefined FMc*: RR 0.93 [0.60 to 1.44], 1 study, n=1076  ***Assisted vaginal birth:***  RR 1.04 [0.65 to 1.66], 1 study, n=1076  ***Adverse effects:***  *Stillbirth rate/cluster:* MD 0.23 [-0.61 to 1.07] , 1 study, n=66  *Perinatal mortality:* RR 0.00 [0.00 to 0.00], 1 study, n=1076 | Low  Score = 13  1 non-critical weakness; no meta-analysis |
| Martis 2017 | To evaluate the effectiveness of different tools for intermittent ausculation (IA) of the fetal heart rate (FHR) during labour including frequency and duration of auscultation. | Pregnant women and their babies. | IA during labour *versus* another method of IA | ***Caesarean section:***  Intermittent CTG vs Pinard for fetal distress: **RR 2.92 [1.78 to 4.80], 1 trial, n=633**  Overall CS: **RR 1.92 [1.39 to 2.64], 1 trial, n=633**  Doppler versus Pinard CS for fetal distress: **RR 2.71 [1.64 to 4.48], 1 study, n=627**  *Overall CS:* RR 1.29 [0.81 to 2.05], 2 studies, n=2598  *Intensive Pinard vs routine Pinard CS for fetal distress:* RR 0.70 [0.35 to 1.38], 1 study, n=625  *Overall CS:* RR 0.71 [0.46 to 1.08], 1 study, n=625  ***Instrumental vaginal birth:***  *Intermittent CTG vs Pinard:* RR 1.46 [0.86 to 2.49], 1 study, n=633  *Doppler vs Pinard:* RR 1.35 [0.78 to 2.32], 1 study, n=627  *Intensive Pinard vs Routine Pinard:* RR 1.21 [0.69 to 2.11], 1 study, n=625  ***Adverse effects:***  *Intermittent CTG vs Pinard- Perinatal mortality:* RR 0.88 [0.34 to 2.25], 1 trial, n=633  *Doppler vs Pinard- Perinatal mortality:* RR 0.69 [0.09 to 5.40], 2 studies, n=2597  *Intensive Pinard vs Routine Pinard - Perinatal mortality:* RR 0.56 [0.19 to 1.67], 1 study, n=625 | High  Score = 15  1 non-critical on 3 |
| Meher 2006a | To assess the effects of exercise and other physical activity on the risk of pre-eclampsia and its complications. | Pregnant women. | Any type of exercise, increased physical activity or advice to exercise *versus* no exercise or normal physical activity | ***Caesarean section:***  *Regular aerobic exercise:* RR: 0.93 [0.22 to 3.88], 1 trial, n=29 | High  Score = 12  1 non-critical; no meta-analysis |
| Meher 2006b | To assess the effects of garlic on prevention of pre-eclampsia and its complications. | Pregnant women, regardless of gestation | Garlic *versus* placebo or no intervention | ***Caesarean section:***  RR 1.35 [0.93 to 1.95], 1 trial, n=100 | Low  Score = 12  1 non-critical weakness; no meta-analysis |
| Meher 2006c | To assess the effects of rest or advice to reduce physical activity during pregnancy for preventing pre-eclampsia and its complications in women with normal blood pressure. | Pregnant women with normal blood pressure, regardless of gestation. | Rest or advice to restrict activity, and combined with other interventions versus unrestricted activity | ***Caesareans section:***  *Rest plus nutrient supplementation vs unrestricted activity and placebo*: RR 0.82 (0.48 to 1.41), 1 triaL, n=74 | High  Score = 13  1 non-critical weakness on 10; no meta-analysis |
| Middleton 2017 | To assess the effects of planned early birth (immediate intervention or intervention within 24 hours) when compared with expectant management (no planned intervention within 24 hours) for women with term PROM on maternal, fetal and neonatal outcomes. | Women with PROM of at least 37 weeks gestation with no specific maternal or fetal contraindications to expectant management. | Planned early birth *versus* expectant management | ***Caesarean section:***  *Any method of IOL:* RR 0.84 [0.69 to 1.04], 23 trials, n=8576  *CS for fetal distress:* RR 0.94 [0.60 to 1.49], 11 trials, n=1851  *Nulliparae:* RR 0.95 [0.76 to 1.18], 6 trials, n=3519  *Multiparae:* RR 0.48 [0.21 to 1.10], 6 trials, n=2370  ***Adverse effects:***  *Perinatal mortality (stillbirth or neonatal mortality):* RR 0.47 [0.13 to 1.66], 8 trials, n=6392  *Serious maternal morbidity or mortality (e.g. death, cardiac arrest, respiratory arrest, admission to intensive care unit):* RR 0.00 [0.00 to 0.00], 3 trials, n=425  *Stillbirth:* RR 0.20 [0.01 to 4.18], 3 trials, n=5314  *Neonatal mortality:* RR 0.20 [0.01 to 4.18], 7 trials, n=6352 | High  Score = 15  1 non-critical weakness on 10 |
| Muktabhant 2015 | To evaluate the effectiveness and safety of diet or exercise, or both, interventions for preventing excessive weight gain during pregnancy. | Pregnant women of any BMI. | Any diet or exercise, or both, intervention (e.g. healthy eating plan, low glycaemic diet, exercise intervention, health education, lifestyle counselling) *versus* standard or routine care | ***Caesarean section:***  *Any intervention:* RR 0.95 [0.88 to 1.03], 28 trials, n=7534  *Low GI diet:* RR 0.99 [0.33 to 3.01], 2 trials, n=133  *Diet and exercise counselling:* RR 0.89 [0.80 to 1.00], 9 trials, n=3406  *Exercise only* *Low risk population:* RR 0.88 [0.63 to 1.21], 1 trial, n=687  *Mixed risk population:* RR 0.96 [0.76 to 1.22], 6 trials, n=2263  *High risk population:* RR 0.98 [0.81 to 1.20], 5 trials, n=645  *Diet and supervised exercise:* RR 1.00 [0.69 to 1.45], 3 trials, n=607  *Diet counselling:* RR 1.06 [0.93 to 1.21], 5 trials, n=754 | High  Score = 15  1 non-critical on 10 |
| Neilson 2015 | To compare the effects of analysis of fetal ECG waveforms during labour with alternative methods of fetal monitoring. | Pregnant women in labour, with a perceived need for continuous EFM. | Any type of fetal ECG waveform analysis, alone or in combination with another method of fetal assessment *versus* alternative method | ***Caesarean section:***  *Fetal ECG plus CTG vs CTG alone:* RR 1.02 [0.96 to 1.08], 6 trials, n=26446  ***Operative delivery:***  **RR 0.92 [0.86 to 0.99], 6 trials, n=26446**  ***Adverse effects:***  *Perinatal mortality:* RR 1.71 [0.67 to 4.33], 6 trials, n=26446 | High  Score = 15  1 non-critical weakness |
| Ota 2015 | To compare the effects on maternal, fetal, neonatal and infant outcomes in healthy pregnant women receiving zinc supplementation, no zinc supplementation, or placebo. | Normal pregnant women with no systemic illness. Women may have had normal zinc levels or they may have been, or likely to have been, zinc deficient. | Zinc supplementation *versus* no supplementation/placebo | ***Caesarean section:***  RR 0.95 [0.58 to 1.53], 6 trials, n=2164  ***Instrumental vaginal birth:***  RR 1.12 [0.79 to 1.59], 1 trial, n=1206  ***Adverse effects:***  *Stillbirth or neonatal mortality:* RR 1.12 [0.86 to 1.46], 8 trials, n=5100 | Moderate  Score = 14  2 non-critical weaknesses on 3, 10 |
| Pattinson 2017 | To assess the effects of pelvimetry (performed antenatally, or intrapartum) on the method of birth, on perinatal mortality and morbidity, and on maternal morbidity. | Pregnant women with a singleton, cephalic presentation fetus who have or have not had a previous caesarean section. | Pelvimetry *versus* no pelvimetry | ***Caesarean section:***  All women: **RR 1.34 [1.19 to 1.52], 5 trials, n=1159**  Women with no previous CS: **RR 1.24 [1.02 to 1.52], 3 trials, n=769**  Women with previous CS: **RR 1.45 [1.26 to 1.67], 2 trials, n=390**  ***Adverse effects:***  *Perinatal mortality*: RR 0.53 [0.19 to 1.45], 5 trials, n=1159 infants | High  Score = 15  1 non critical on 3 |
| Phipps 2014 | To assess the effect of prophylactic manual rotation for women with malposition in labour on mode of delivery, and maternal and neonatal outcomes. | Women at term planning a vaginal birth with a cephalic singleton fetal malposition in labour. | Prophylactic manual rotation in labour for fetal malposition *versus* expectant management | ***Caesarean section:***  RR 1.33 [0.36 to 4.97], 1 study, n=30  ***Operative delivery (any):***  RR 1.08 [0.79 to 1.49], 1 study, n=30  ***Adverse effects:***  *Maternal mortality:* 0, 1 study  *Perinatal mortality:* 0, 1 study | High  Score = 13  1 non-critical on 10, no meta-analysis conducted |
| Rafael 2014 | To assess whether the use of a cervical stitch in multiple gestations at high risk of pregnancy loss based on woman’s history, ultrasound findings of ‘short cervix’, or physical exam changes in the cervix improves obstetrical and perinatal outcomes. | All women with multiple gestations. | Cervical cerclage *versus* no cerclage | ***Caesarean section:***  RR 1.24 [0.65 to 2.35], 3 trials, n=77  ***Adverse effects:***  *Perinatal mortality:* RR 1.74 [0.92 to 3.28], 5 trials, n=262  *Serious neonatal morbidity:* RR 0.96 [0.13 to 7.10], 3 trials, n=116  *Stillbirth (fetal demise after 20 weeks’ gestation, prior to delivery):* RR 0.26 [0.01 to 5.26], 4 trials, n=188  *Neonatal mortality (after birth, and before 29 days of neonatal life or discharge from hospital):* RR 1.60 [0.69 to 3.74], 4 trials, n=188 | Low  Score = 13  1 critical on 15, 2 non-critical weaknesses on 3, 10 |
| Raman 2017 | To compare the effects of different methods and settings for glucose monitoring for women with GDM on maternal and fetal, neonatal, child and adult outcomes, and use and costs of health care. | Women diagnosed with GDM during their current pregnancy. | Different methods (including timings and frequencies) or settings, or both, for blood glucose monitoring. | ***Caesarean section:***  *Telemedicine vs standard care:* RR 1.05 [0.72 to 1.53], 5 trials, n=478  ***Operative vaginal birth:***  *Telemedicine vs standard care*: RR 0.50 [0.11 to 2.30], 1 trial, n=47  ***Adverse effects:***  *Perinatal mortality:* RR 0.00 [0.00 to 0.00], 2 trials, n=131  *Death or serious morbidity composite:* RR 1.06 [0.68 to 1.66], 1 trial, n=57  *Stillbirth:* RR 0.41 [0.02 to 9.55], 3 trials, n=178 *Neonatal mortality:* RR 0.0 [0.00 to 0.00], 2 trials, n=131 | High  Score = 15  1 non-critical weaknesses |
| Rumbold 2015a | To evaluate the effects of vitamin C supplementation, alone or in combination with other separate supplements on pregnancy outcomes, adverse events, side effects and use of health resources. | All pregnant women receiving either vitamin C supplementation or control either in areas where there is inadequate dietary intake or where there is presumed adequate intake. | Vitamin C supplementation, alone or in combination with other separate supplements *versus* placebo/no placebo | ***Caesarean section:***  RR 1.02 [0.97 to 1.07], 9 trials, n=16459  *Pre-labour CS:* RR 1.15 [0.85 to 1.56], 2 trials, n=1932  ***Adverse effects:***  *Stillbirth:* RR 1.15 [0.89 to 1.49], 11 trials, n=20038 *Neonatal mortality:* RR 0.79 [0.58 to 1.08], 11 trials, n=19575  *Perinatal mortality:* RR 1.07 [0.77 to 1.49], 7 trials, n=17271  *Maternal mortality (up to 6 weeks’ postpartum):* RR 0.60 [0.14 to 2.51], 7 trials, n=17120  *Serious maternal morbidity eclampsia:* RR 1.42 [0.72 to 2.78], 9 trials, n=20304 | Moderate  Score = 14  2 non-critical weaknesses on 3, 10 |
| Rumbold 2015b. | To assess the effects of vitamin E supplementation, alone or in combination with other separate supplements, on pregnancy outcomes, adverse events, side effects and use of health services. | Pregnant women receiving vitamin E supplementation or control, living in areas where there is either inadequate dietary intake of vitamin E or where there is presumed adequate intake. | Vitamin E supplementation, alone or in combination with other separate supplements *versus* placebo/no placebo | ***Caesarean section:***  *Pre-labour CS:* RR 1.15 [0.85 to 1.56], 2 trials, n=1932  *Overall:* RR 1.02 [0.97 to 1.07], 6 trials, n=15297  ***Adverse effects:***  *Stillbirth:* RR 1.17 [0.88 to 1.56], 9 trials, n=19023;  *Neonatal mortality:* RR 0.81 [0.58 to 1.13], 9 trials, n=18617  *Perinatal mortality:* RR 1.09 [0.77 to 1.54], 6 trials, n=16923  *Infant mortality:* RR 3.02 [0.12 to 74.12], 1 trial, n=2694  *Measures of serious maternal morbidity (eclampsia):* RR 1.67 [0.82 to 3.41], 8 trials, n=19471 | Moderate  Score = 14  2 non-critical weaknesses on 3, 10 |
| Smith 2003 | To determine, the effectiveness and safety of homoeopathy for third trimester cervical ripening and IOL. | Pregnant women due for 3^rd^ trimester IOL. | Homoeopathy *versus* placebo | ***Caesarean section:***  RR 5.0 [0.26 to 98.00], 1 trial, n=40  ***Instrumental delivery:***  RR 1.0 [0.54 to 1.86], 1 trial, n=40 | Low  Score = 11  1 critical flaw on 4 (p-yes); 2 non-critical on 3, 10; no meta-analysis |
| Smith 2006 | To examine the effects of complementary and alternative therapies for pain management in labour. | All women in spontaneous or induced labour, in the first and second stage of labour. | Complementary and alternative therapies used in labour (but not biofeedback) with or without concurrent use of pharmacological or non-pharmacological interventions *versus* placebo or no treatment | ***Caesarean section:***  *Acupuncture:* RR 0.96 [0.06 to 14.83], 1 trial, n=90  *Aromatherapy:* RR 2.54 [0.11 to 56.25], 1 trial, n=22  Hypnosis: **RR 0.46 [0.30 to 0.72], 1 trial, n= 520**  ***Instrumental vaginal birth:***  *Acupuncture:* RR 0.95 [0.45 to 2.00], 2 trials, n=288 *Aromatherapy:* RR 0.83 [0.06 to 11.70], 1 trial, n=22  *Relaxation:* RR 0.16 [0.01 to 2.68], 1 trial, n=34  ***Spontaneous vaginal birth:***  *Acupuncture:* RR 0.98 [0.89 to 1.08], 1 trial, n=90  *Aromatherapy:*  RR 0.93 [0.67 to 1.28], 1 trial, n=22  Hypnosis: **RR 1.32 [1.19 to 1.46], 3 trials, n=645**  ***Satisfaction:***  *Acupuncture:* RR 1.08 [0.95 to 1.22], 1 trial, n=90  *Hypnosis:* RR 2.33 [1.15 to 4.71], 1 trial, n=125  *Massage:* MD -0.47 [-1.07 to 0.13], 1 trial, n=60 | Low  Score=11  1 critical on 15, 4 non-critical weaknesses on 3, 10, 12, 14 |
| Smith 2011a | To examine the effects of acupuncture and acupressure for pain management in labour. | Women in labour. | Acupuncture or acupressure *versus* placebo, no treatment | ***Caesarean section:***  *Acupuncture vs Placebo:* RR 1.39 [0.62 to 3.10], 3 trials, n=448  *Standard care:* RR 0.86 [0.47 to 1.60], 2 trials, n=506  *No treatment:* RR 0.76 [0.35 to 1.83], 1 trial, n=163 Acupressure vs Placebo***:* RR 0.24 [0.11 to 0.54], 1 trial, n=120**  ***Assisted vaginal birth:***  *Acupuncture vs placebo:* RR 0.64 [0.27 to 1.50], 1 trial, n=208  *Standard care:* RR 0.67 [0.46 to 0.98], 3 trials, n=704 *No treatment:* RR 0.49 [0.18 to 1.38], 1 trial, n=163  *Acupressure vs placebo:* RR 1.00 [0.87 to 1.14], 2 trials, n=325  *Standard care:* RR 0.98 [0.89 to 1.08], 1 trial, n=90  ***Satisfaction:***  *Acupressure vs placebo:* MD 4.80 [-2.29 to 11.89], 1 trial, n=211 | Moderate  Score = 14  2 non-critical |
| Smith 2011b | To examine the effects of aromatherapy for pain management in labour on maternal and perinatal morbidity. | Women in labour. | Aromatherapy *versus* placebo/no treatment | ***Caesarean section:***  RR 0.98 [0.49 to 1.94], 1 trial, n=513  ***Assisted vaginal birth:***  *Aromatherapy vs standard care:* RR 1.04 [0.48 to 2.28], 1 trial, n=513 | High  Score = 12  1 non-critical; no meta-analysis conducted |
| Smith 2011c | To examine the effects of relaxation techniques for pain management in labour on maternal and perinatal morbidity. | Women in labour. | Relaxation techniques *versus* placebo/no treatment/usual care | ***Caesarean section:***  *Music vs control:* RR 1.25 [0.37 to 4.21], 1 trial, n=60  *Relaxation x 3 trials, but data not pooled:*  i) RR 0.13 [0.02 to 0.93], n=52  ii) RR 5.71 [0.71 to 45.84], n=34  iii) *Cluster trial:* RR 0.91 [0.71 to 1.18], n=904  ***Assisted vaginal birth:***  *Relaxation:* RR 0.07 [0.01 to 0.50], 2 trials, n=86  *Cluster trial:* RR 1.15 [0.82 to 1.61], 1 trial, n=904  ***Satisfaction:***  *Relaxation satisfaction with pain relief in labour*: RR 8.0 [1.10 to 58.19], 1 trial, n=40  *Satisfaction with childbirth experience:* MD -0.40 [-3.47 to 2.67], 1 trial, n=904 | High  Score = 15  1 non-critical weakness on 10 |
| Smith 2012 | To assess the effect, safety and acceptability of massage, reflexology and other manual healing methods to manage pain in labour. | Women in labour. | Any type of manual  healing method *versus* Placebo/no treatment or usual care | ***Caesarean section:***  *Massage vs Usual care:* RR 0.73 [0.24 to 2.22], 2 trials, n=105  ***Assisted vaginal birth:***  RR 0.46 [0.14 to 1.50], 2 trials, n=105  ***Satisfaction with pain relief:***  MD 0.47 [-0.13 to 1.07], 1 study and MD -14.40 [-32.70 to 3.90], 1 trial | Moderate  Score = 14  2 non-critical on 3, 10 |
| Smith 2017 | To determine the effectiveness and safety of acupuncture and acupressure for third trimester cervical ripening or IOL. | Pregnant women carrying a viable fetus due for third trimester IOL. | Manual, laser, or electro-acupuncture or acupressure *versus* placebo, no treatment, sham-acupuncture /acupressure | ***Caesarean section:***  *Acupuncture vs Sham control:* RR 0.80 [0.56 to 1.15], 8 trials, n=789  *Acupuncture vs usual care:* RR 0.77 [0.51 to 1.17], 8 trials, n=760  *Acupressure vs sham control:* RR 1.02 [0.68 to 1.53], 2 trials, n=151  ***Instrumental vaginal birth:***  *Acupuncture vs Sham:* RR 1.16 [0.83 to 1.62], 5 trials, n=610  *Acupuncture vs usual care:* RR 0.77 [0.51 to 1.17], 8 trials, n=760  ***Spontaneous vaginal birth:***  *Acupuncture vs sham:* RR 1.21 [0.93 to 1.57], 3 trials, n=495  *Acupuncture vs usual care:* RR 1.44 [0.70 to 2.98], 2 trials, n=117  *Acupressure vs usual care:* RR 0.97 [0.69 to 1.34], 2 trials, n=151  ***Adverse effects:***  *Maternal or perinatal mortality:* RR 0.00 [0.00 to 0.00], 1 trial, n=364  ***Satisfaction:***  *Acupuncture vs sham:* RR 1.29 [0.99 to 1.67], 1 trial, n=67 | High  Score = 16  No weaknesses |
| Smyth 2013 | To determine the effectiveness and safety of amniotomy alone for routinely shortening all labours that start spontaneously. | Pregnant women with singleton pregnancies regardless of parity  and gestation in spontaneous labour. | Amniotomy *versus* no amniotomy | ***Caesarean section:***  RR 1.27 [0.99 to 1.63], 9 trials, n=5021  *Primiparae:* RR 1.15 [0.88 to 1.51], 6 trials, n=2674  *Multiparae:* RR 1.76 [0.65 to 4.76], 2 trials, n=1473  ***CS for fetal distress:***  RR 3.21 [0.66 to 15.60], 2 trials, n=690  ***CS for prolonged labour:***  RR 0.45 [0.07 to 3.03], 1 trial, n=690  ***Instrumental vaginal birth:***  RR 0.99 [0.87 to 1.13], 10 trials, n=5121  ***Adverse effects:***  *Maternal mortality:* RR 3.01 [0.12 to 73.61], 3 trials, n=1740  *Perinatal mortality:* RR 3.01 [0.12 to 73.59], 8 trials, n=3397  ***Maternal satisfaction:***  *With childbirth experience:* MD -1.10, [-7.15 to 4.95], 1 trial, n=84 | Low  Score = 13  1 critical weakness on 15, 2 non-critical weakness on 12, 14 |
| Stock 2016 | To assess the effects of immediate versus deferred delivery of preterm babies with suspected fetal compromise on neonatal, maternal and long-term outcomes. | Pregnant women at > 36 weeks’ in whom there is clinical suspicion of fetal compromise | Immediate delivery *versus* deferred delivery for a set period of time, until test results worsen, or expectant management. | ***Caesarean section:***  **RR 1.15 [1.07 to 1.24], 1 trial, n=547**  ***Adverse effects:***  *Extended perinatal mortality (IUD death in the first 28 days of life):* RR 1.17 [0.67 to 2.04], 1 trial, n=587  *Composite outcome of death or disability at or after two years:* RR 1.22 [0.85 to 1.75], 1 trial, n=573  Stillbirth: **RR 0.22 [0.05 to 1.00], 1 trial, n=587** *Neonatal mortality:* RR 1.84 [0.93 to 3.62], 1 trial, n=576. | High  Score = 13  1 non-critical on 10; no meta-analysis |
| Thomas 2014 | To determine the effects of vaginal prostaglandins E2 and F2a for third trimester cervical ripening or IOL. | Pregnant women due for third trimester IOL, carrying a viable fetus. | Vaginal prostaglandins E2 and F2a *versus* placebo/no treatment | ***Caesarean section:***  *PGE2 vs Placebo:* 13.5% versus 14.8%, RR 0.91 [0.81 to 1.02], 36 trials, n=6599  *Primiparae:* RR 0.93 [0.77 to 1.12], 10 trials, n=2486  *Multiparae:* RR 0.82 [0.48 to 1.42], 5 trials, n=1298  *Intact membranes:* RR 1.13 [0.82 to 1.57], 6 trials, n=816  *ROM:* RR 0.89 [0.73 to 1.08], 7 trials, n=3320 *Unfavourable cervix:* RR 0.87 [0.75 to 1.02], 22 trials, n=2173  *Favourable cervix:* RR 1.13 [0.40 to 3.18], 2 trials, n=401  *PGF2a vs placebo:* RR 0.59 [0.31 to 1.14], 4 trials, n=467  ***Instrumental vaginal birth:***  *PGE2 vs placebo:* RR 0.95 [0.82 to 1.10], 13 trials, n=4219;  PGF2a vs placebo: **RR 0.63 [0.47 to 0.84], 3 trials, n=435**  ***Adverse effects****:*  *PGE2 vs Placebo: Serious neonatal morbidity or perinatal mortality:* RR 0.46 [0.09 to 2.31], 9 trials, n=3638  *Serious maternal morbidity or mortality:* RR 2.23 [0.34 to 14.76], 3 trials, n=530  *Perinatal mortality:* RR 0.56 [0.14 to 2.22], 7 trials, n=3648  *Serious maternal complication:* RR 2.90 [0.12 to 68.50], 1 trial, n=59  ***Satisfaction:***  *PGE2 vs placebo- woman not satisfied:* RR 0.76 [0.24 to 2.40], 2 trials, n=2922 | High  Score = 15  1 non-critical |
| Tieu 2017 | To assess the effects of dietary advice interventions for preventing GDM and associated adverse health outcomes for women and their babies. | Pregnant women, excluding women with pre-existing type 1 or type 2 diabetes. | Interventions that assessed any type of dietary advice before testing for GDM *versus* no dietary advice intervention (i.e. standard care) | ***Caesarean section:***  *Dietary advice vs Standard care*: RR 0.98 [0.78 to 1.24], 4 trials, n=1194  ***Adverse effects:***  *Perinatal mortality:* RR 0.0 [0.00 to 0.00], 1 trial, n=159  *Stillbirth:* RR 3.09 [0.13 to 75.65], 2 trials, n=959 | Moderate  Score = 14  2 non-critical weaknesses on 3, 10 |
| Till 2015 | To determine whether incentives are an effective tool to increase utilization of timely prenatal care among women. | Pregnant women. | Direct incentives explicitly linked to initiation and frequency of prenatal care (e.g. cash, vouchers, coupons or products not generally offered to patients as a standard of prenatal care) *versus* no incentives | ***Caesarean section:***  **RR 1.97 [1.18 to 3.30], 1 study, n=979** | High  Score = 12  1 non-critical on 10; no meta-analysis |
| Torvaldsen 2004 | To assess the effectiveness of discontinuing epidural analgesia late in labour. | Pregnant women of any age who receive an epidural for labour analgesia in the first stage of labour. | Any epidural (any dosage regimen, any method of administration) that is discontinued late in labour (at least 8cm cervical dilatation) and replaced by either placebo or no treatment *versus* continuation of the same epidural protocol until birth | ***Caesarean section:***  *Discontinued vs continued:* RR 0.98 [0.43 to 2.25], 4 trials, n=282  ***Instrumental vaginal birth:***  *23% versus 28%:* RR 0.84 [0.61 to 1.15], 5 trials, n=462  ***Spontaneous vaginal birth:***  RR 1.11 [0.95 to 1.30], 4 trials, n=282 | Low  Score = 13  1 critical on 15, 2 non-critical weaknesses on 3, 10 |
| Vogel 2017 | To examine pharmacological and mechanical interventions to induce labour or ripen the cervix in outpatient settings. | Pregnant women (with a viable fetus) at or near team (>35 weeks) in an outpatient setting. | Different methods of IOL in outpatient settings *versus* placebo, expectant management or routine care | ***Caesarean section:***  *Intravaginal PGE-gel vs placebo*: RR 0.80 [0.49 to 1.31], 4 studies, n=288  *Intracervical PGE:* RR 0.90 [0.72 to 1.12], 7 studies, n=674  *Vaginal misoprostol:* RR 0.94 [0.61 to 1.46], 4 studies, n=325  *Oral Misoprostol:* RR 0.88 [0.62 to 1.25], 5 studies, n=343  *Oestrogens:* RR 1.27 [0.63 to 2.58], 1 study, n=87 *Vaginal isosorbide mononitrate:* RR 0.99 [0.87 to 1.14], 6 studies, n=2286  *Outpatient ARM:* RR 1.20 [0.78 to 1.86], 1 study, n=521  ***Instrumental vaginal birth:***  *Intracervical PGE:* RR 1.29 [0.85 to 1.96], 4 studies, n=538  *Vaginal misoprosotol:* RR 0.91 [0.50 to 1.67], 2 studies, n=145  *Oral Misoprostol:* RR 1.35 [0.93 to 1.97], 5 studies, n=343  *Oestrogens:* RR 0.84 [0.44 to 1.60], 1 study, n=87  *Vaginal isosorbide mononitrate:* RR 0.81 [0.61 to 1.07], 2 studies, n=1712  *Outpatient ARM:* RR 0.70 [0.46 to 1.08], 1 study, n=521  ***Adverse effects:***  *Vaginal misoprostol perinatal mortality:* RR 0.34 [0.01 to 8.14], 1 study, n=77  *Mifipristone serious neonatal morbidity or mortality:* RR 1.56 [0.07 to 35.67], 1 study, n=36  *Vaginal isosorbide mononitrate perinatal mortality:* RR 1.61 [0.08 to 33.26], 2 studies, n=1712  ***Satisfaction:***  *Vaginal isosorbide mononitrate felt satisﬁed (very or extremely):* RR 0.80 [0.67 to 0.94], 1 study, n=1049 *Outpatient ARM maternal satisfaction – ‘I look back positively on the treatment I received’:* RR 1.04 [0.97 to 1.10], 1 study, n=404  ***Costs:***  *Vaginal isosorbide mononitrate total cost of care package (GBP):* MD 11.98 [-105.34 to 129. 30], 1 study, n=350 | High  Score = 15  1 non-critical on 10 |
| Weaver 2013 | To explore the impact of planned interventions on pregnant women with tokophobia who have requested a CS. | Pregnant women requesting a CS for tokophobia in the absence of medical (or obstetric) indications. | Planned interventions in reducing levels of fear and/or anxiety of women with tokophobia *versus* no intervention or alternative treatment | ***Fear of childbirth:***  *Intensive therapy versus conventional therapy:* Birth related concern reduced in the intensive therapy group, but increased in the conventional therapy group; **(p=0.022) (1 study, n=176)** | Moderate  Score = 8  4 non-critical weaknesses, no meta-analysis conducted |
| Wei 2013 | To estimate the effects of a policy of early augmentation with amniotomy and oxytocin (prevention) on the caesarean birth rate and on indicators of maternal and neonatal morbidity. | Pregnant women in spontaneous labour. | Early augmentation with amniotomy and oxytocin *versus* conservative form of management | ***Caesarean section:***  *Overall:* RR 0.89 [0.79 to 1.01], 14 trials, n=8033 Prevention studies: **RR 0.87 [0.77 to 0.99], 11 trials, n=7753**  *Therapy studies:* RR 1.47 [0.73 to 2.96], 3 trials, n=280  ***Spontaneous vaginal birth:***  RR 1.01 [0.97 to 1.05], 12 trials, n=6020 ***Instrumental vaginal birth:***  RR 1.01 [0.92 to 1.12], 12 trials, n=6018 | Low |
| Whitworth 2015 | To assess routine early pregnancy ultrasound for fetal assessment. | Women with early pregnancies, i.e. less than 24 weeks gestation. | Routine US *versus* selective US | ***Caesarean section:***  RR 1.05 [0.98 to 1.12], 5 trials, n=22193  ***Adverse effects:***  *Perinatal mortality:* RR 0.89 [0.70 to 1.12], 10 trials, n=35735  ***Satisfaction:***  Mother not satisfied with care: **RR 0.80 [0.65 to 0.99], 1 trial, n=634** | High  Score = 15  1 non-critical on 10 |
| Wojcieszek 2014 | To assess the effects of antibiotics administered prophylactically to women with prelabour rupture of the membranes at 36 weeks’ gestation or beyond | Women with SROM prior to the onset of regular uterine contractions at 36 weeks’ gestation or beyond | Any antibiotics, administered as prophylaxis, by any route *versus* placebo/no antibiotics | ***Caesarean section:***  RR 1.33 [1.09 to 1.61], 3 studies, n=1906  ***Operative vaginal birth:***  RR 0.95 [0.63 to 1.44], 3 studies, n=1906  ***Adverse effects:***  *Stillbirth:* RR 3.00 [0.61 to 14.82], 3 studies, n=1906  *Perinatal mortality:* RR 1.98 [0.60 to 6.55], 4 studies, n=2639  *Neonatal mortality:* RR 0.00 [0.00 to 0.00], 3 studies, n=1906 | Moderate  Score = 14  2 non-critical weakness |
