# Supplementary material for: Antenatal and intrapartum interventions for reducing caesarean section, promoting vaginal birth, and reducing fear of childbirth: An overview of systematic reviews
Source: PLoS One. 2019 Oct 24;14(10):e0224313. doi: 10.1371/journal.pone.0224313 (PMC6812784; doi:10.1371/journal.pone.0224313)
Supplement: S2 Table — (DOCX) [file pone.0224313.s003.docx]

**S2 Table: Summary characteristics of 54 excluded reviews based on AMSTAR-2 critically low rating**

| ***Author, year*** | ***Aim*** | ***Population*** | ***Intervention versus comparator*** | ***Reported outcomes and results*** | ***AMSTAR-2*** |
| --- | --- | --- | --- | --- | --- |
| Al-Mandeel 2013 | To assess the effects of intentional delivery (ID) over expectant management (EM) in pregnancies complicated by preterm pre-labour rupture of membranes (PPROM) between 28 and 34 weeks of gestation on maternal and neonatal outcomes. | Pregnant women with PPROM, 28-34 weeks of gestation and their babies. | Intentional delivery (i.e. planned early birth soon after PPROM via IOL or by caesarean section) *versus* expectant management | ***Caesarean section:***  **RR 1.35 [1.02 to 1.80], 5 trials, n=488**  ***Adverse effects:***  *Neonatal mortality:* RR 1.87 [0.63 to 5.52], 5 trials, n=488 | Critically low  Score = 10  3 critical flaws on 7, 9, 13; 3 non-critical flaws on 8 (p-yes), 10, 12 |
| Alwan 2009 | To compare the effect of alternative treatment policies for GDM on both maternal and infant outcomes | Pregnant women diagnosed with GDM or impaired glucose tolerance. | Alternative treatment policies for GDM *versus* usual care | ***Caesarean section:***  *Any policy vs usual care:* RR 0.95 [0.80 to 1.12], 5 trials, n=1255  ***Operative vaginal birth:***  *Specific dietary advice vs usual care:* RR 1.13 [0.07 to 17.26], 1 trial, n=68  ***Adverse effect:***  *Perinatal mortality:* RR 0.09 [0.01 to 1.70], 1 trial, n=1030 | Critically low  Score = 11  2 critical flaws on 13 and 15; 3 non-critical flaws on 8 (p-yes), 10, 12 |
| Blix 2016 | To quantify the efficacy of ST waveform analysis versus CTG. | Women in labour. | ST waveform analysis plus CTG *versus* CTG alone | ***Caesarean section:***  *Overall:* RR 1.02 [0.96 to 1.08], 6 trials, n=26446  *CS for fetal distress:* RR 0.93 [0.78 to 1.12], 6 trials, n=26446  ***Instrumental vaginal birth:***  **RR 0.92 [0.86 to 0.99], 6 trials, n=26446**  ***Adverse effects:***  *Perinatal and neonatal mortality:* RR 1.79 [0.69 to 4.64], 6 trials, n=26 446 | Critically low  Score = 4  2 critical on 4 (p-yes), 15; 2 non-critical on 10, 3 |
| Boulvain 2005 | To determine the effects of membrane sweeping for third trimester IOL. | Pregnant women due for third trimester IOL and their babies. | Membrane sweeping used for third trimester cervical ripening or IOL *versus* placebo/no treatment | ***Caesarean section:***  RR 0.90 [0.70 to 1.15], 18 trials, n=2389  *In all women, unfavourable cervix:* RR 0.98 [0.49 to 1.95], 3 trials, n=200  *Primiparae:* RR 0.68 [0.33 to 1.42], 2 trials, n=378 ***Instrumental vaginal birth:***  RR 1.15 [0.94 to 1.42], 14 trials, n=1842  ***Adverse effects:***  *Perinatal mortality:* RR 1.00 [0.20 to 4.88], 6 trials, n=800  *Serious neonatal morbidity/perinatal mortality:* RR 1.28 [0.32 to 5.14], 6 trials, n=830  *Serious maternal morbidity or mortality:* RR 0.00 [0.00 to 0.00], 2 trials, n=263 | Critically low  Score = 12  2 critical flaws on 4 (p-yes), 15; 2 non critical on 6, 10 |
| Boulvain 2008 | To determine the effects of intracervical prostaglandins for third trimester cervical ripening or IOL compared with placebo/no treatment and with vaginal prostaglandins (except misoprostol). | Pregnant women and their babies. | Intracervical prostaglandins for third trimester cervical ripening or IOL *versus* placebo/no treatment (vaginal prostaglandins except misoprostol) | ***Caesarean section:***  *Placebo/no treatment*: **RR 0.88 [0.77 to 1.00],** 27 trials, n=3734  *Unfavourable cervix:* RR 0.88 [0.77 to 1.01], 27 trials, n=3716  *Primiparae:* RR 1.27 [0.64 to 2.52], 3 trials, n=122  *Multiparae:* RR 2.18 [0.44 to 10.76], 1 trial, n=46  ***Vaginal delivery not achieved within 24hrs:*** **RR 0.61 [0.47 to 0.79],** 4 trials, n=198  ***Instrumental vaginal delivery:***  RR 1.03 [0.70 to 1.50], 7 trials, n=551  ***Adverse effects:***  *Perinatal mortality:* RR 0.20 [0.01 to 4.05], 2 trials, n=1081  *Serious neonatal morbidity or perinatal mortality:* RR 0.75 [0.19 to 2.96], 4 trials, n=1193  *Maternal mortality:* 0, 1 trial, n=816  *Serious maternal morbidity or mortality:* RR 0.33 [0.01 to 7.96], 2 trials, n=1081 | Critically low  Score = 10  3 critical flaws on 4 (p-yes), 13 and 15; 3 non-critical on 5, 6, 10 |
| Brancato 2008 | To compare the effectiveness of passive descent versus early pushing following epidural in second-stage labour. | Pregnant women with epidural in labour and their babies. | Passive descent *versus* early or immediate pushing | ***Caesarean section:***  RR 0.80 [0.57 to 1.12], 7 trials, n=2827  ***Spontaneous vaginal birth:***  **RR 1.08 [1.01 to 1.15],** 7 trials, n=2827  ***Instrumental birth:***  RR 0.77 [0.77 to 0.85], 7 trials, n=2827 | Critically low  Score = 6  5 critical flaws on 2, 4 (p-yes), 7, 13, 15; 4 non-critical on 5, 6, 10, 14 |
| Cantor 2015 | To examine evidence from U.S.-relevant populations on the effectiveness of routine supplementation and screening for IDA in pregnancy. | Asymptomatic pregnant women receiving screening or supplementation for IDA. | Screening for IDA or routine iron supplementation v*ersus* not screening or no routine iron supplementation | ***Caesarean section:***  CS ranged from 7.6% to 26% in the supplementation groups and from 9.1% to 33% in the placebo (5 trials; no MA). In 1 trial (n=1164), fewer women receiving 60 mg of elemental iron daily vs placebo had CS (25.2% vs. 33.1%; OR 0.58 [0.37 to 0.89]. | Critically low  Score = 9  4 critical on 2, 4 (p-yes), 7, 9; 3 non-critical on 1, 6, 10 |
| Catling-Paull 2011 | To review clinical interventions that increase the uptake and/or the success rates of vaginal birth after caesarean section | Pregnant women and their babies. | Clinical interventions designed to increase the uptake and/or the success of VBAC rates versus no or different intervention:  1. PG vs expectant care  2. Oxytocin vs no oxytocin  3. 200 mg mifepristone v placebo,  4. Antenatal x-ray pelvimetry vs no x-ray  5. Double vs. single layer closure | ***Caesarean section:***  1. *VBAC:* "No difference", 2 trials, n=336  2. *VBAC:* 77% with no oxytocin, 80% with oxytocin, P = NS, 1 trial, n=197  3. *VBAC:* 69% with mifepristone, 50% with placebo, P = NS, 1 trial, n=32  4. VBAC: **16% with X-ray pelvimetry, 42% with no x-ray, OR 3.8 [2.1 to 6.8], 1 trial, n=228**  5. *VBAC:* 56% with single layer, 64% with double layer, no difference, 1 trial, n=145 | Critically low  Score = 7  2 critical flaws on 2, 7; no meta-analysis; 2 non-critical flaws on 5, 10 |
| Caughey 2009 | To evaluate maternal, fetal and neonatal risks of elective IOL compared with expectant management. | Pregnant women and their babies. | Elective IOL, defined as an IOL ≥37 weeks and <42 weeks of gestation without either a maternal or fetal indication for IOL *versus* Expectant management /SOL | ***Caesarean section:***  *Elective IOL vs expectant RCTs:* **OR 1.22 [1.07 to 1.39], 9 trials, n=6138**  *Women at or beyond 41 0/7 weeks of gestation:* **OR 1.21 [1.01 to 1.46],** 6 trials, n=5481  *Less than 41 weeks:* OR 1.73 [0.67 to 4.50], 2 trials, n=573 *<1990 vs >1990:* No difference, 8 trials, n=6054  *Nulliparae:* OR 1.67 [0.81 to 3.46], 3 trials, n=506  **Observational Studies:** *Elective IOL vs expectant management:* **1% vs 6.7%**  Elective IOL vs SOL: **OR 0.63 [0.49 to 0.79], 12** **observational studies, n=32499** Nulliparae: **OR 0.48 [0.41 to 0.56], 11 studies (10 retrospective and 1 prospective), n=33168**  Multiparae: **OR 0.78 [0.63 to 0.65], 10 studies, n=20617**  ***Instrumental birth:***  OR 0.91 [0.79 to 1.04], 6 trials, n=4798  ***Adverse effects:***  Neonatal mortality, 6 trials: 4 in expectant management; 0 in elective IOL; 0.5% vs 1.7%, 1 retrospective cohort study, n=unclear | Critically low  Score = 12  3 critical flaws on 2, 4, 7; 1 non-critical on 10 |
| Chaillet & Dumont 2007 | To assess the effectiveness of interventions for reducing  the caesarean section rate. | Pregnant women and their babies. | Strategy or interventions for reducing overall CS *versus* usual care | ***Caesarean section:***  Overall: **RR 0.81 [0.75 to 0.87], 10 trials,** **n=776909**  **Audit and feedback:** **RR 0.87 [0.81 to 0.93], 4 trials**  Quality improvement: **RR 0.74 [0.70 to 0.77], 4 trials** | Critically low  Score = 11  2 critical flaws on 2, 4 (p-yes); 3 non-critical on 5, 10, 16 |
| Cluett & Burns 2009 | To assess the evidence from randomised controlled trials about immersion in water during labour and water birth on maternal, fetal, neonatal and caregiver outcomes. | Pregnant women, singleton, in labour and their babies. | Immersion in water 1^st^ or 2^nd^ stage *versus* no immersion | ***Caesarean section:***  *Immersion in 1^st^ stage*: RR 1.21 [0.87 to 1.68], 8 trials, n=2712  *Immersion in 2^nd^ stage:* RR 0.33 [0.07 to 1.52], 2 trials, n=180  ***Assisted vaginal birth:***  *Immersion in 1^st^ stage:* RR 0.86 [0.71 to 1.05], 7 trials, n=2628  *Immersion in 2nd stage:* RR 0.73 [0.21 to 2.54], 2 trials, n=180  ***Spontaneous vs operative vaginal birth:***  Immersion in first stage: **RR 1.26 [1.09 to 1.45],** **1 trial, n=106**  ***Adverse effects:***  *2nd stage Immersion - Perinatal mortality:* RR 3.0 [0.12 to 72.20], 1 trial, n=120  ***Satisfaction:***  *2nd stage satisfaction with labour and birth on scale of 0-6 where 0 is not at all satisfied:* RR 0.03 [-0.64 to 0.70], 1 trial, n=60 | Critically low  Score = 12  1 critical flaw on 15; 1 non-critical on 10 |
| Dodd 2010 | To assess the benefits and harms of antenatal dietary or lifestyle interventions for pregnant women who are overweight or obese. | Pregnant women who are overweight or obese. | Antenatal dietary intervention *versus* usual care | ***Caesarean section:***  RR 1.09 [0.93 to 1.28], 5 trials, n=540 | Critically low  Score = 12  2 critical flaws on 2, 15; 2 non-critical on 3, 10 |
| Domenjoz 2014 | To determine whether structured physical training programs during pregnancy can improve the course of labour and delivery. | Pregnant women. | Structured physical exercise programs (minimum described) *versus* no programs | ***Caesarean section:***  Overall: **RR 0.85 [0.73 to 0.99],** **16 trials,** **n=3037** Intervention of >50 hours: **RR 0.76 [0.58 to 0.99], 5 trials**  ***Instrumental birth:***  RR 1.00 [0.82 to 1.22], 8 trials, n=2083 | Critically low  Score = 11  3 critical flaws on 2, 4 (p-yes), 7; 2 non-critical 10, 16 |
| Duley 2010 | To assess the effects of magnesium sulphate, and other anticonvulsants, for prevention of eclampsia. | Pregnant women with pre-eclampsia. | Anticonvulsant drug, or other agents used specifically to prevent eclampsia versus placebo (or no anticonvulsant), or one anticonvulsant drug with another | ***Caesarean section:***  Magnesium sulphate all women with pre-eclampsia: **RR 1.05 [1.01 to 1.10],** **6 trials, n=10096**  ***Adverse effects:***  *Magnesium sulphate maternal mortality:* RR 0.54 [0.26 to 1.10], 2 trials, n=10795  *Serious maternal morbidity:* RR 1.08 [0.89 to 1.32], 2 trials, n=10332  *Stillbirths:* RR 0.99 [0.87 to 1.12], 3 trials, n=9961*Perinatal mortality:* RR 0.98 [0.88 to 1.10], 2 trials, n=9259  *Neonatal mortality:* RR 1.16 [0.94 to 1.42], 1 trial, n=8260 | Critically low  Score = 12  2 critical flaws on 13, 15,; 2 critical flaws on 10, 14 |
| Fortier & Godwin 2015 | To determine the effect of support provided by doulas on the rate of medical interventions during labour for low-risk women intending to deliver vaginally at term. | Low risk pregnant women. | Presence of a trained doula in labour *versus* usual care | ***Caesarean section:***  **OR 0.68 [0.47 to 0.99], 5 trials, n=2008**  Sensitivity analysis high quality trials: **OR 0.63 [0.49 to 0.81], 3 trials, n=1560**  ***Instrumental vaginal birth:***  **OR 0.56 [0.35 to 0.92], 4 trials, n=1587** | Critically Low  Score = 13  2 critical flaws on 2, 4 (p-yes), 1 non-critical on 10 |
| Hartling 2006 | To evaluate the effect of intentional delivery versus expectant management in women with preterm pre-labour rupture of membranes (PPROM) at 30-36 weeks. | Women with PPROM between 30-36 weeks of gestation. | Intentional delivery; initiated within 24 hours after admission for PPROM via any mode *versus* expectant management (described) | ***Caesareans section:***  RD 0.05 [-0.01 to 0.11], 4 trials, n=389  ***Adverse effects:***  *Neonatal mortality:* RD 0.03 [0.00 to 0.05], 4 trials, n=391  *Perinatal mortality:* RD -0.02 [-0.04 to 0.01], 4 trials, n=391 | Critically low  Score = 14  2 critical weaknesses on 2 and 7 |
| Howarth & Botha 2001 | To determine the efficacy and safety of amniotomy and intravenous oxytocin for third trimester IOL. | Women due for third trimester IOL, with a viable fetus. | IV (intravenous) oxytocin and amniotomy *versus* placebo/no treatment | ***Caesarean section:***  RR 4.0 [0.46 to 35.11], 1 trial, n=184  ***Adverse effects:***  *Serious neonatal morbidity or perinatal mortality:* RR 0.33 [0.01 to 8.08], 1 trial, n=184  ***Satisfaction:***  *Woman not satisfied:* RR 1.11 [0.77 to 1.60], 1 trial, n=186 | Critically low  Score = 11  2 critical weaknesses on 13, 15; 3 non-critical on 5, 6, 10 |
| Hunter 2007 | To assess the effects of adopting a hands and knees maternal posture in late pregnancy or during labour when the presenting part of the foetus is in a lateral or posterior position compared with no intervention. | Women with lateral or posterior fetal positions in late pregnancy or labour, or women with any fetal positions (preventive). | Variations of the hands and knees posture to prevent or correct fetal malposition *versus* no intervention/ alternative postures | ***Caesarean section:***  *Hands and knees posture in pregnancy:* RR 0.98 [0.80 to 1.20], 1 trial, n=2547  ***Instrumental birth:***  *Hands and knee posture in pregnancy:* RR 1.07 [0.88 to 1.31], 1 trial, n=2547 | Critically low  Score = 10  2 critical on 11, 15; 4 non-critical on 6, 10, 12, 14 |
| Hutton & Mozurkewich 2001 | To determine the effects of extra-amniotic prostaglandin for third trimester cervical ripening or IOL. | Pregnant women due for third trimester IOL carrying a viable foetus. | Extra-amniotic prostaglandin *versus* placebo/no treatment | ***Caesarean section:***  *Overall Extra-amniotic PGE2:* RR 0.56 [0.26 to 1.20], 3 trials, n=176  *Unfavourable cervix:* RR 0.50 [0.14 to 1.78], 2 trials, n=60  *Primiparae:* RR 0.40 [0.09 to 1.75], 1 trial, n=30  *PGF2 alpha vs extra-amniotic placebo gel all women:* RR 0.33 [0.03 to 3.20], 1 trial, n=25  *Unfavourable cervix:* RR 0.33 [0.03 to 3.20], 1 trial, n=25  *Primiparae:* RR 0.33 [0.03 to 3.20], 1 trial, n=25  ***Instrumental vaginal birth:***  RR 1.14 [0.56 to 2.35], 1 trial, n=30  ***Adverse effects:***  *Perinatal mortality:* RR 2.06 [0.09 to 46.11], 1 trial, n=25 | Critically low  Score = 11  3 critical on 4 (p-yes), 13, 15; 2 non-critical on 6, 10 |
| Hutton 2009 | To determine if sterile water injection for low back pain compared to placebo or alternative therapy increased or decreased the rate of CS. | Pregnant women with lower back pain during the active stage of labour who requested pain medication. | Intracutaneous or subcutaneous injections of sterile *versus* Placebo injection of isotonic saline, or other non-pharma methods of pain relief such as TENS, or acupuncture | ***Caesarean section:***  **RR 0.51 [0.30 to 0.87], 8 trials, n=828** | Critically low  Score = 11  3 critical on 2, 4 (p-yes), 15; 2 non-critical on 10, 12 |
| Kehl 2016 | To review the evidence about efficacy and safety of balloon catheters for cervical ripening and IOL in women with a previous caesarean section. | All pregnancies at term with a previous CS. | Balloon catheter for cervical ripening *versus* no intervention | ***Caesarean section:***  **OR 2.63 [2.24 to 3.10], 4 studies, n=680** | Critically low  Score = 5  6 critical on 2, 4 (p-yes), 7, 9, 13, 15; 5 non-critical on 5, 6, 10, 12, 14 |
| Kraemer 2004 | To evaluate the relationship of health care delivery system characteristics and legal factors to mode of delivery in women with prior caesarean section. | Pregnant women with previous CS and clinicians (varied populations). | Various health care system attributes that may influence rates of TOL, VBAC, or RCD (legislative  and legal system factors, practice guidelines, provider and hospital characteristics, and aspects of insurance coverage or payment source) *versus* no intervention, usual care, other intervention | ***Caesarean section:***  *Repeat CS rate - External review:* 10.9% (n=45 hospital) vs 9.8% (n=120 hospitals)  SOGC 6 years pre-guideline RCD rate : -0.041 (0.008) vs 2 years post guideline RCD rate: -0.113 monthly rate of change per 100 women  Adj RCD private teaching (n=883) versus public (n=2625) vs HMOs (n=84) 40% vs 43% vs 59% | Critically low  Score = 9  2 critical flaws on 2, 13; 2 non-critical; no meta-analysis |
| Magro-Malosso 2017a | To evaluate the effect of exercise on the risk of PTB in overweight and obese pregnant women. | Obese pregnant women with a mean BMI ≥25 kg/m2, singleton pregnancies, no obstetric contraindication to physical activity. | Exercise *versus* no exercise | ***Caesarean section:***  RR 0.93 [0.77 to 1.10], 7 studies, n=1208  ***Adverse effects:***  *Stillbirth:* RR 2.13 [0.22 to 20.4], 2 studies, n=693 | Critically Low  Score = 12  2 critical on 7, 15 and 2 non-critical |
| Magro-Malosso 2017b | To evaluate the effects of IOL for suspected fetal macrosomia. | Pregnant women with suspected fetal macrosomia, and/or no contras for vaginal. | IOL *versus* expectant management | ***Caesarean section:***  RR 0.91 [0.76 to 1.09], 4 trials, n=1190  ***Operative vaginal birth:***  RR 0.86 [0.65 to 1.13], 4 trials, n=1190  ***Spontaneous vaginal birth:***  RR 1.09 [0.99 to 1.20], 4 trials, n=1190 | Critically low  Score = 13  2 critical on 4 (p-yes), 7; 1 non-critical on 10 |
| Makvandi 2016 | To summarize and critically discuss the evidence from randomized controlled trials (RCTs) regarding the effects of acupressure on the duration of labour and the mode of delivery. | Nulliparous or multiparous women with healthy full-term pregnancies in the first stage of labour. | Acupressure at any acupoint during childbirth *versus* placebo/no intervention | **Caesarean Section:**  Not reported  Chance of vaginal birth: **OR 2.33 [1.35 to 4.02], 9 studies** | Critically low  Score = 9  3 critical on 2, 4 (p-yes), 7; 4 non-critical on 1, 3, 10, 16 |
| McDonagh 2005 | To evaluate the risks and benefits of IOL in women with a prior caesarean delivery. | Women with prior CS. | Induction or augmentation of labour by drug or mechanical means *versus* placebo, expectant management, spontaneous labour | ***Caesarean section:***  31% mifepristone vs 50% control, 1 study;  20% of those undergoing SOL (range 11–35%) compared with 32% who received oxytocin (range 18–44%), 7 studies, n=8047  PGE2 vs SOL: **(33% vs 49%) RR 2.11 [1.89 to 2.35], 1 study, n=5022**  RR 1.12 [0.51 to 2.32], 1 study, n=81  ***Adverse effects:***  Zero cases of maternal mortality reported in any study.  Of 13 studies reporting infant mortality: 2 studies found the rate higher in the SOL groups (0.15% and 0.18% vs 0). | Critically low  Score = 12  3 critical on 2, 4 (p-yes), 15; 1 non-critical on 10 |
| Mishanina 2014 | To investigate whether the risk of caesarean delivery is higher or lower following IOL compared with expectant management. | Women with a viable singleton pregnancy. | IOL *versus* expectant management | ***Caesarean section:***  Overall: **RR 0.88 [0.84 to 0.93], 157 trials, n=31085).**  **Subgroup analysis - Methods associated with a significant reduction in risk of CS:**  ***-*** PGE2: **RR 0.90 [0.84 to 0.96], 67 trials**  ***-*** Misoprostol: **RR 0.62 [0.48 to 0.81], 11 trials**  ***Adverse effects:***  Fetal mortality: **RR 0.50 [0.25 to 0.99], 60 trials**  *Maternal mortality:* RR 1.00 [0.10 to 9.57], 20 trials | Critically low  Score = 11  2 critical flaws on 2, 4 (p-yes); 3 non-critical on 3, 10. 16 |
| Nilsson 2015 | To evaluate the effectiveness of women-centred interventions during pregnancy and birth to increase rates of vaginal birth after caesarean. | Pregnant women who have had at least one previous CS. | Any women-centred intervention, used during pregnancy or birth, that was designed to increase VBAC rate *versus* Standard or usual care or an alternative intervention | ***VBAC:***  *Info vs usual care:* RR 0.97 [0.75 to 1.27], 1 study, n=402  *Decision analysis*: RR 1.24 [0.97 to 1.58], 1 study, n=399  *Written info vs AN education:* RR 1.08 [0.97 to 1.21], 1 study, n=1275 | Critically low  Score = 10  2 critical on 2, 4 (p-yes); 1 non-critical on 10; no meta-analysis |
| O'Brien 2016 | To identify the effect of providing an antenatal dietary and lifestyle intervention for women of normal BMI on maternal and infant outcomes. | All pregnant women with a singleton pregnancy and a normal BMI (BMI 18.5–24.9 kg/m2). | Dietary and/or lifestyle intervention(s) *versus* standard care/no intervention | ***Caesarean section:***  RR 0.92 [0.58 to 1.45], 2 trials, n=243  ***Adherence:***  67% of women completed or attended all four intervention sessions, 1 study, n=219  All questionnaires completed, 1 study, n=236) | Critically low  Score = 6  2 critical on 2, 4 (p-yes); 2 non-critical on 3, 10 |
| Oteng-Ntim 2012 | To determine the efficacy of antenatal dietary, activity, behaviour or lifestyle interventions in overweight and obese pregnant women to improve maternal and perinatal outcomes. | Overweight and obese pregnant women. | Antenatal dietary, activity, behaviour or lifestyle interventions *versus* Standard care, or no intervention | ***Caesarean section:***  OR 0.96 [0.68 to 1.36], 6 trials, n=663  OR 1.13 [0.78 to 1.64], 4 studies, n=1246 | Critically low  Score = 7  3 critical on 4 (p-yes), 7, 15; 2 non-critical on 10, 14 |
| Pérez-López 2015 | To assess the effects of vitamin D supplementation during pregnancy on obstetric outcomes and birth variables. | Pregnant women. | Vitamin D supplementation/calcium/ vitamins/ferrous sulfate *versus* placebo | ***Caesarean section:***  RR 0.94 [0.78 to 1.13], 4 studies, n=1028 | Critically low  Score = 9  3 critical on 2, 4, 7, and 4 non-critical on 3, 8, 10, 16 |
| Poolsup 2014 | To assess the efficacy and safety of treating pregnant women with gestational diabetes mellitus in comparison to usual antenatal care. | Women with GDM. | Any therapeutic intervention *versus* usual care | ***Caesarean section:***  **RR 0.90 [0.80 to 1.00], 8 studies, n=2997**  ***Adverse effects:***  *Perinatal/neonatal mortality:* RR 0.65 [0.36 to 1.18], 5 studies, n=3101 | Critically Low  Score = 9  3 critical on 2, 4, 7; 3 non-critical on 5, 8, 10 |
| Poyatos-León 2015 | To examine the influence of physical exercise interventions on the mode of delivery of healthy pregnant women with low to moderate levels of physical activity. | Pregnant women. | Supervised exercise programs *versus* no program | ***Caesarean section:***  RR 0.78 [0.58 to 1.05], 10 studies, n=3160  ***Normal birth/SVD:***  **1.12 [1.01 to 1.24], 8 studies, n=1770**  ***Operative birth:***  RR 0.88 [0.68 to 1.15], 6 studies, n=1637 | Critically Low  Score = 10  4 critical on 2, 4 (partial yes), 7, 9 (partial yes), and 2 non-critical |
| Rossi | To systematically review the literature about maternal and neonatal outcomes following IOL and SOL in women with previous caesarean section. | Women with at least one previous caesarean section | IOL *versus* SOL | ***Caesarean section:***  **OR 1.52 [1.26 to 1.83], 7 studies, n=14317**  ***Vaginal birth:***  **OR 0.66, [0.55 to 0.80], 7 studies, n=14317**  ***Operative vaginal birth:***  OR 0.98 [0.49 to 1.97], 2 studies, n=2349  ***Adverse effects:***  *Neonatal morbidity:* OR 1.13 [0.75 to 1.69], 4 studies, n=11922  *Maternal morbidity:* OR 1.18 [0.74 to 1.89], 2 studies, n=9585 | Critically low  Score = 7  4 critical on 2, 4 (p-yes), 7, 15; 4 non-critical on 3, 8, 10, 12, 16 |
| Rossignol 2014 | To review, critically appraise, and synthesize the literature on the epidemiological relations between four intrapartum obstetric interventions; EFM, epidural analgesia, IOL and labour acceleration; and two types of delivery, instrumental and CS. | Not defined. | Obstetric intervention *versus* no intervention | ***Caesarean section:***  EFM on admission: **RR 1.20 [1.00 to 1.44], 3 trials, n=11259**  ***Epidural and CS (3 meta-analyses):***  **i) RR 1.60 [1.18 to 2.18], 27 trials, n=23860**  ii) RR 1.52 [0.98 to 2.35], 6 trials, n=866  iii) RR 1.04 [0.80 to 1.35], 13 trials, n=3276  **Oxytocin for IOL (4 meta- analyses):**  **i) RR 1.17 [1.01 to 1.35], 24 trials**  **ii) RR 0.82 [0.72 to 0.93], 8 trials**  **iii) RR 0.89 [0.81 to 0.97], 21 trials**  **iv) RR 0.87 [0.79 to 0.96], 9 trials**  Oxytocin for acceleration: **RR 0.88 [0.77 to 0.99], 10 trials, n=7653** | Critically low  Score = 6  4 critical on 2, 4 (p-yes), 7, 15; 4 non-critical on 1, 5, 8, 10; no meta-analysis |
| Saccone 2015 | To evaluate the risk of caesarean and any maternal and perinatal effects of a policy IOL in uncomplicated full-term singleton gestations. | Women at term. | Policy of IOL *versus* Expectant management | ***Caesarean section:***  RR 1.25 [0.75 to 2.08], 5 studies, n=844  *Favourable cervix:* RR 0.95 [0.39 to 2.32], 2 studies, n=310  *Nulliparae:* RR 1.67 [0.94 to 2.95], 2 studies, n=200  ***Spontaneous birth***  RR 0.95 [0.87 to 1.02], 4 studies, n=700  ***Operative Vaginal birth:***  RR 1.22 [0.83 to 1.81], 4 studies, n=700  ***Adverse effects:***  *Perinatal mortality:* RR 0.35 [0.04 to 3.37], 4 studies, n=800 | Critically low  Score = 9  3 critical on 2 (p-yes), 4 (p-yes), 7; 3 non-critical on 3, 5, 10 |
| Saccone 2016 | To compare the effectiveness of cardiotocography plus ST analysis with cardiotocography alone during labour. | Singleton gestations in cephalic presentation at term or near term in labour. | Intrapartum electronic fetal monitoring with cardiotocography plus ST waveform analysis  (STAN group) *versus* Cardiotocography alone (control group). | ***Caesarean section:***  RR 0.96 [0.85 to 1.08], 6 studies, n=26446  ***Operative vaginal birth:***  **RR 0.91 [85 to 0.98], 6 studies, n=26446**  ***Adverse effects*:**  *Perinatal composite outcome:* RR 0.90 [0.74 to 1.10], 4 studies, n=24095  *Perinatal mortality:* RR 1.71 [0.67 to 4.33], 6 studies, n=26410 | Critically Low  Score = 10  3 critical on 2 (p-yes), 4 (p-yes), 7 and 3 non-critical |
| Salmelin 2013 | To assess the evidence that  computerized ST analysis during labour reduces the incidence of fetal metabolic acidosis, HIE, caesarean sections, instrumental vaginal deliveries and the number of women who require FBS as compared with CTG only. | Women in active labour with term pregnancy, a singleton fetus in cephalic presentation, ruptured membranes and the need for continuous fetal monitoring. | Computerised ST analysis *versus* CTG only | ***Caesarean section:***  *For fetal distress:* RR 0.93 [0.80 to 1.08], 4 studies, n=12904  *Non-fetal indications:* RR 0.98 [0.86 to 1.12], 4 studies, n=12904 | Critically low  Score = 9  4 critical on 2, 4 (partial yes), 9, 13; 3 non-critical on 3, 10, 16 |
| Sanchez-Ramos 2002 | To systematically review the effects of expectant management and IOL on mode of delivery and perinatal outcomes in patients with suspected fetal macrosomia. | Women with macrosomia | Expectant management *versus* IOL for suspected fetal macrosomia | ***Caesarean section:***  Observational studies: **OR 0.39 [0.30 to 0.50], 9 studies, n=3438**  *RCTs:* OR 1.17 [0.69 to 2.01], 2 studies, n=313  ***Spontaneous vaginal birth:***  Observational studies: **OR 2.07 [1.34 to 3.19], 8 studies, n=3252**  *RCTs:* OR 0.90 [0.54 to 1.48], 1 study, n= 273  ***Operative vaginal birth:***  *Observational studies:* OR 0.89 [0.68 to 1.17], 8 studies, n=3252  *RCTs:* OR 1.02 [0.50 to 2.08], 1 study, n=273 | Critically low  Score = 9  4 critical on 2 (p-yes), 4 (p-yes), 7, 9 and 3 non-critical |
| Sanchez-Ramos 2003 | To compare routine IOL with expectant management for pregnant women who exceed 41 weeks gestation. | Women who were having an uncomplicated, singleton pregnancy who had reached 41 weeks gestation. | IOL versus expectant management | ***Caesarean section:***  **OR 0.88 [0.78 to 0.99], 15 trials**  CS for FHR abnormalities: **OR 0.77 [0.61 to 0.96], 7 trials**  ***Adverse effects:***  *Perinatal mortality:* OR 0.41 [0.14 to 1.18], 13 studies | Critically low  Score = 7  4 critical on 2, 4 (p-yes), 7, 9; 5 non-critical on 5, 8, 10, 12, 16 |
| Schuit 2013 | To assess the effectiveness of electronic fetal monitoring (EFM) alone and with additional ST analysis. | Labouring women with a singleton term pregnancy that is in cephalic presentation. | EFM + ST analysis *versus* EFM alone | ***Caesarean section:***  RR 0.99 [0.91 to 1.09], 4 studies, n=12987  ***Instrumental vaginal birth:***  **RR 0.90 [0.83 to 0.99], 4 studies, n=12987**  ***Adverse effects:***  *Perinatal mortality:* RR 1.24 [0.33 to 4.61], 4 studies, n=12987 | Critically Low  Score = 9  4 critical on 2 (p-yes), 4 (p-yes), 7, 15, & 3 non-critical |
| Stoll 2017 | To summarize high quality studies of nonpharmacological prenatal interventions that might be linked to reductions in PSA/FoB during pregnancy. | Not defined/pregnant women. | Intervention, educational  component, or treatment regime for PSA/FoB *versus* control | ***Fear of childbirth:***  Interventions shown to be effective for reducing FoC: 16hr antenatal education programme, extended AN education; 8-week antenatal yoga course, Telephone psycho-education delivered by midwives (1.5-5 hours) | Critically Low  Score = 11  2 critical on 2, 7 & 3 non-critical |
| Thangaratinam 2012 | To evaluate the evidence on dietary and lifestyle interventions to reduce weight or prevent weight gain in pregnancy | Not explicit, but states pregnant women who were underweight (BMI < 18.5 kg/m2) were excluded. | Any dietary, physical activity or behavioural counselling intervention with the potential to influence weight change in pregnant women *versus* no intervention | ***Caesarean section:***  RR 0.93 [0.85 to 1.03], *29 RCTs:* | Critically low  Score = 9  3 critical on 2, 4 (p-yes), 7; 4 non-critical on 1, 3, 8, 10 |
| Thomas 2001 | To determine the effectiveness and safety of oestrogens alone, or with amniotomy, for third trimester cervical ripening and IOL in comparison with other methods of IOL. | Pregnant women due for third trimester IOL, carrying a single live fetus. | Oestrogens alone or with amniotomy for cervical ripening or IOL *versus* placebo/no treatment | ***Caesarean section***  *Placebo* RR 1.00 [0.60 to 1.68], 5 studies, n=306  *Unfavourable cervix:* RR 1.14 [0.63 to 2.09], 3 studies, n=162  *Primiparae:* RR 0.22 [0.04 to 1.32], 2 studies, n=69  *Multiparae:* RR 1.0 [0.07 to 15.12], 1 study, n=50  **Instrumental vaginal delivery:**  RR 0.75 [0.46 to 1.22], 3 studies, n=162  ***Adverse effects:***  *Serious neonatal morbidity or perinatal mortality:* RR 1.0 [0.07 to 15.12], 2 studies, n=75  *Perinatal mortality:* RR 0.33 [0.01 to 7.81], 2 studies, n=75  *Serious maternal complications:* RR 2.93 [0.85 to 10.10], 1 study, n=87 | Critically low  Score = 11  3 critical on 4 (p-yes), 13, 15; non-critical on 3, 5 |
| Thorne-Lyman 2012 | To assess vitamin D during pregnancy and perinatal and infant health outcomes. | Not defined. | Vitamin D supplement; control not explicit | ***Caesarean section:***  Women with vitamin D levels 25(OH)D < 37.5 nmol/L during pregnancy: (**adjusted OR 3.84 [1.71 to 8.62]), 1 observational study**  No difference in a second observational study | Critically low  Score = 6  4 critical on 2, 4 (p-yes), 7, 15; 1 non-critical on 4, 3, 5, 6, 10; no meta-analysis |
| Tuuli 2012 | To estimate whether immediate or delayed pushing in the second stage of labour optimizes spontaneous vaginal delivery and other perinatal outcomes. | Women in the 2nd stage of labour. | Delayed pushing *versus* immediate pushing | ***Caesarean section:***  RR 0.85 [0.63 to 1.14], 10 studies, n=3099  ***Spontaneous vaginal birth:***  **61.5% vs 56.9%, RR 1.09 [1.03 to 1.15], 12 studies, n=3099**  ***Instrumental vaginal birth:***  RR 0.89 [0.76 to 1.06], 10 studies, n=3099 | Critically Low  Score = 10  3 Critical on 2 (p-yes), 4 (p-yes), 7; 3 non-critical 1, 8, 10 |
| Waldenstrom 1998 | To examine the evidence for alternative models of maternity care characterised by continuity of midwifery care. | No clear description/pregnant women. | Continuity of midwifery care *versus* standard pattern of care | ***Caesarean section:***  RR 0.91 [0.78 to 1.05], 7 trials, n=8703  ***Instrumental vaginal birth:***  **RR 0.82 [0.70 to 0.95], 7 trials, n=8703**  ***Adverse effects:***  *Perinatal mortality:* OR 1.60 [0.99 to 2.59], 7 trials, n=8730  *Stillbirth:* OR 1.72 [0.90 to 3.32]  *Neonatal mortality:* OR 1.27 [0.49 to 3.34] | Critically Low  Score = 8  4 critical on 2, 7, 13, 15 & 4 non-critical flaws |
| Walker 2002 | To review and report on the success of strategies to address global CS rate. | Pregnant women in the antenatal or intrapartum period in hospital delivery ward. | Any intervention directed at CS rates | ***Caesarean section:***  Continuous support vs usual care: **OR 0.77 [0.64 to 0.91], 1 study, n=5000**  *Community-based VBAC education and support program with pamphlet:* No differences | Critically low  Score = 6  4 critical on 2, 7, 9, 13; no meta-analysis; 4 non-critical |
| Walker 2016 | IOL versus expectant management in subgroups of women over 35 to reduce risk of CS. | Women between 37-42 week's gestation, intact membranes, singleton. | IOL *versus* expectant management | ***Caesarean section:***  OR 1.20 [0.74 to 2.00], 5 studies, n=367 | Critically low  Score = 9  3 critical on 7, 13 (p-yes), 9; 4 non-critical on 6, 10, 12, 16 |
| Wennerholm 2009 | To compare perinatal and maternal outcomes between elective IOL versus expectant management of pregnancies at 41 weeks and beyond. | Pregnant women 41 weeks or beyond. | Elective IOL *versus* expectant management | ***Caesarean section:***  **RR 0.87 [0.80 to 0.96], 13 trials, n=6617**  *Caesarean Section (SR DATA):*  i) **OR 0.88 [0.78 to 0.99]**, **16 trials**  ii) *41 weeks:* RR 0.92 [0.76 to 1.12], 19 trials  iii) *42 weeks:* RR 0.97 [0.72 to 1.31], 19 trials  ***Assisted vaginal birth:***  RR 1.05 [0.95 to 1.17], 7 studies, n=4716  ***Adverse effects:***  *Perinatal mortality:* RR 0.33 [0.10 to 1.09], 11 studies  *Perinatal mortality (SR DATA):*  i) RR 0.41 [0.14 to 1.18], 16 trials  **ii) RR 0.30 [0.09 to 0.99], 19 trials**  iii) RR 0.33 [0.10 to 1.09], 13 trials  ***Satisfaction:***  **RR 1.93 [1.62 to 2.30], 1 trial, n= 496** | Critically low  Score = 10  3 critical on 2, 4 (p-yes), 7; 3 non-critical on 3, 10, 14 |
| Wei 2009 | To estimate the effects of early augmentation with oxytocin for slow progress of labour on the delivery method and on indicators of maternal and neonatal morbidity. | Pregnant women in labour and without prior use of oxytocin. | Early augmentation of labour with oxytocin *versus* more conservative form of management | ***Caesarean section:***  RR 0.87 [0.71 to 1.06], 9 trials, n=1983  ***Spontaneous vaginal birth:***  **RR 1.09 [1.03 to 1.17], 8 trials, n=1903**  ***Operative vaginal birth:***  **RR 0.84 [0.70 to 1.00], 8 trials, n=1903**  ***Adverse effects:***  *Fetal or neonatal mortality:* RR 2.33 [0.35 to 15.6], 3 trials, n=1638 | Critically low  Score = 9  4 critical on 2, 4 (p-yes), 13, 15; 3 non-critical on 3, 10, 12 |
| Whitworth 2011 | To assess the value of specialised antenatal clinics for women with a pregnancy at high risk of preterm delivery when compared with ’standard’ antenatal clinics. | Pregnant women with a singleton pregnancy considered by the trial authors to be at high risk of preterm labour. | Specialised antenatal clinics *versus* standard clinics | ***Caesarean section:***  RR 1.11 [0.76 to 1.62], 1 trial, n=376  ***Adverse effects:***  *Perinatal mortality:* RR 1.25 [0.43 to 3.62], 1 trial, n=302  ***Cost:***  *Cost with care:* MD -16.67 [-367.00 to 335.67], 2 trials, n=604 | Critically low  Score = 12  2 critical weakness on 4 (p-yes), 15, 2 non-critical on 3, 10 |
| Witkop 2009 | To estimate benefits and harms of the choice of timing of IOL or elective caesarean delivery based on estimated fetal weight or gestational age in women with GDM. | Not clearly defined. | IOL (but not clearly defined) *versus* not clearly defined (probably expectant) | ***Caesarean section:***  Active IOL versus expectant: **25% vs 31%, P=0.43** | Critically Low  2 critical on 2, 7 and 3 non-critical |
| Wood 2014 | To evaluate whether IOL, compared with a policy of expectant management, in women with intact membranes increased the rate of caesarean section. | Women with intact membranes (low and high risk). | Policy of IOL, for indications other than preterm rupture of membranes (PROM) *versus* expectant management | ***Caesarean section:***  **OR 0.83 [0.76 to 0.92], 31 trials, n=12166**  CS in post-dates: **OR 0.85 [0.76 to 0.95], 19 trials, n=7812**  CS with IOL for other indications: **OR 0.81 [0.69 to 0.95], 12 trials, n=4354**  ***Operative vaginal birth:***  RR 1.09 [0.98 to 1.22], 20 trials, n=8816  ***Adverse effects:***  *Perinatal mortality:* OR 0.37 [0.14 to 1.00], 30 trials, n=12054 | Critically Low  Score = 12  2 critical on 4 (p-yes), 7; 2 non-critical on 3, 10 |
| Xu 2007 | To evaluate if AI reduces meconium aspiration syndrome (MAS) and other indicators of morbidity in babies born to women with meconium-stained amniotic fluid (MSAF). | Women with meconium stained liquor. | Prophylactic amnioinfusion *versus* no amnioinfusion | ***Caesarean section:***  *All women:* RR 0.85 [0.70 to 1.03], 12 trials, n=4029  *CS with standard peripartum surveillance:* RR 0.89 [0.73 to 1.10], 10 trials, n=3184  *CS limited peripartum surveillance:* RR 0.70 [0.49 to 1.00], 2 trials, n=845 | Critically low  Score = 11  2 critical on 2, 4 (p-yes); 3 non-critical on 1, 10, 16 |
