# Supplementary material for: Antenatal and intrapartum interventions for reducing caesarean section, promoting vaginal birth, and reducing fear of childbirth: An overview of systematic reviews
Source: PLoS One. 2019 Oct 24;14(10):e0224313. doi: 10.1371/journal.pone.0224313 (PMC6812784; doi:10.1371/journal.pone.0224313)
Supplement: S3 Table — (DOCX) [file pone.0224313.s004.docx]

**S3 Table: Summary results for 10 included Practice Guidelines**

| ***Reference*** | ***Aim*** | ***Recommendations*** | ***AGREE***  ***Overall*** |
| --- | --- | --- | --- |
| ACOG 2016 | To provide information about ECV and to make recommendations regarding its use in obstetric practice | - All women who are near term with breech presentations should be offered an ECV if there are no contraindications ***(Level A Evidence)*** - Previous caesarean is not associated with a lower rate of success; however, the magnitude of the risk of UR is not known *(Level B Evidence)* | 83% |
| ACOG 2017 | To review the risks and benefits of trial of labour after caesarean (TOLAC) in various clinical situations and to provide practical guidelines for counselling and management of women who will attempt to give birth vaginally after a previous caesarean birth | - Most women with one previous caesarean with a low-transverse incision are candidates for and should be counselled about and offered TOLAC ***(Level A Evidence)*** - Misoprostol should not be used for cervical ripening or IOL in women at term who have had a caesarean or major uterine surgery ***(Level A Evidence)*** - Those at high risk of uterine rupture (UR) and those in whom vaginal birth is otherwise contraindicated are not generally candidates for planned TOLAC *(Level B Evidence)* - It is reasonable to consider women with two previous low-transverse caesareans to be candidates for TOLAC and to counsel them based on the combination of other factors that affect their probability of achieving a successful VBAC *(Level B Evidence)* - Women with one previous caesarean with an unknown uterine scar type may be candidates for TOLAC, unless there is a high clinical suspicion of a previous classical uterine incision *(Level B Evidence)* - Women with one previous caesarean with a low-transverse incision, who are otherwise appropriate candidates for twin vaginal birth, are considered candidates for TOLAC *(Level B Evidence)* - IOL remains an option in women undergoing TOLAC *(Level B Evidence)* - ECV for breech presentation is not contraindicated in women with a prior low-transverse uterine incision who are candidates for ECV and TOLAC *(Level B Evidence)* - After counselling, the decision to undergo TOLAC or a repeat caesarean should be made by the woman in consultation with her obstetrician or obstetric care provider. - The potential risks and benefits of both TOLAC and elective repeat caesarean section (ERCS) should be discussed. Documentation of counselling and the management plan should be included in the medical record *(Level C Evidence)* - Women attempting TOLAC should be cared for in a level 1 centre or higher/a centre capable of performing emergency deliveries *(Level C Evidence)* - Because of the unpredictability of complications requiring emergency medical care, home birth is contraindicated for women undergoing TOLAC *(Level C Evidence)* | 67% |
| ACOG 2017a | Review of the evidence for labor care practices that facilitate a physiologic labor process and minimize intervention for appropriate women who are in spontaneous labor at term. | - Evidence suggests that, in addition to regular maternity care, continuous one-to-one emotional support is associated with improved outcomes for women in labor - In the absence of an indication for expeditious birth, women (particularly those who are nulliparous with epidural analgesia) may be offered a period of rest of 1–2 hours (unless the woman has an urge to bear down sooner) at the onset of the second stage of labor - Clinicians should be familiar with and consider using low-interventional approaches, when appropriate, for the intrapartum management of low-risk women in spontaneous labor. Many low-interventional approaches such as continuous support in labour, IA of FHR, reduce the incidence of caesarean | 54% |
| Caughey 2015 | To assist health care providers to understand the short-term and long-term trade-offs between caesarean and vaginal birth, as well as the safe and appropriate opportunities to prevent overuse of caesarean particularly primary caesarean | ***First stage of labor***   - A prolonged latent phase (> 20 hours in nulliparous and > 14 hours in multiparous) should not be an indication for caesarean *[Level 1B evidence]* - Slow but progressive labor should not be an indication for caesarean *[Level 1B evidence]* - Cervical dilation of 6cm should be considered the threshold for the active phase of most women in labor. Thus, < 6 cm of dilation, standards of active phase progress should not be applied *[Level 1B evidence]* - Caesarean birth for active phase arrest in the 1st stage should be reserved for women at or > 6 cm dilation with rupture of membranes who fail to progress despite 4hrs of adequate uterine activity, or at least 6hrs of oxytocin administration with inadequate uterine activity and no cervical change *[Level 1B evidence]*   ***Second stage labor***   - Before diagnosing arrest of labor in the 2nd stage, if the maternal and fetal conditions permit, allow for the following: At least 2 hours of pushing in multiparous women *[Level 1B evidence]* - At least 3 hours of pushing in nulliparous women *[Level 1B evidence]* - Instrumental vaginal birth (IVB) by experienced and well trained physicians should be considered a safe, acceptable alternative to caesarean. Training in, and ongoing maintenance of, practical skills related to IVB should be encouraged *[Level 1B evidence]* - Manual rotation of the fetal occiput in the setting of fetal malposition is a reasonable intervention to consider before moving to IVB or caesarean *[Level 1B evidence]* - FHR monitoring of decelerations may safely reduce the rate of caesarean ***[Level 1A evidence]*** - Scalp stimulation can be used as a means of assessing fetal acid–base status when abnormal FH patterns are present and is a safe alternative to caesarean in this setting *[Level 1C evidence]*   ***IOL***   - Before 41 weeks, IOL generally should be performed based on maternal and fetal medical indications - IOL at 41 weeks and beyond should be performed to reduce the risk of caesarean and the risk of perinatal morbidity and mortality ***[Level 1A evidence]*** - Cervical ripening methods should be used when labor is induced in women with an unfavorable cervix *[Level 1B evidence]* - If the maternal and fetal status allow, caesarean for failed IOL in the latent phase can be avoided by allowing longer durations of the latent phase (up to 24 hours or longer) and requiring that oxytocin be administered for at least 12–18 hours after rupture of membranes before deeming the induction a failure *[Level 1B evidence]*   ***Fetal malpresentation***   - Fetal presentation should be assessed and documented beginning at 36 0/7 weeks to allow for ECV to be offered *[Level 1C evidence]*   ***Suspected fetal macrosomia***   - Caesarean to avoid potential birth trauma should be limited to estimated fetal weights of at least 5000 g in women without diabetes and at least 4500 g in women with diabetes *[Level 2C evidence]*   ***Other***   - Individuals, organizations, and governing bodies should work to ensure that research is conducted to provide a better knowledge base to guide decisions regarding caesarean and to encourage policy changes that safely lower the rate of primary caesarean *[Level 1C evidence]* | 86% |
| FIGO 2016 | Use of the 10-Group Classification System (TGCS) for caesarean birth | - The TGCS for the audit of caesareans should be instituted by all healthcare facilities and organizations responsible for delivering maternity care - FIGO encourages the TGCS results to be made available for purposes such as quality control and auditing - FIGO encourages maternal and neonatal events and outcomes, including morbidity and mortality, to be recorded and made available. | 28% |
| Hauk 2015 | To provide information on health outcomes associated with labour after caesarean (LAC) or planned VBAC | - Counselling, encouragement and facilitation for a planned VBAC should be provided so that women can make informed decisions. If LAC/VBAC is not locally available, then women desiring it should be offered referral to a facility or clinician who can offer the service - Indications for and circumstances surrounding the prior caesarean should be discussed - IOL after caesarean is appropriate for women who have a medical indication for IOL and who are planning LAC/VBAC. Misoprostol should not be used for cervical preparation or IOL in the third trimester of pregnancy for women with a prior caesarean - Women should be informed of the specific short-term and long-term benefits and harms of planned VBAC for the patient, her fetus/infant, and future pregnancies - Hospitals should have guidelines to promote access to LAC/VBAC and actively monitor and improve quality of care for women who choose LAC | 28% |
| Kotaska 2009 | To review the physiology of breech birth; to discern the risks and benefits of a trial of labour (TOL) versus planned caesarean and to recommend selection criteria, intrapartum management parameters, and delivery techniques for a trial of vaginal breech birth | - Vaginal breech birth can be associated with a higher risk of perinatal mortality and short-term neonatal morbidity than elective caesarean ***(Level I Evidence)*** - Is reasonable in selected women with a term singleton breech fetus ***(Level I Evidence)*** - For a woman with suspected breech presentation, pre- or early labour ultrasound should be performed to assess type of breech presentation, fetal growth and estimated weight, and attitude of fetal head. If ultrasound is not available, caesarean is recommended *(Level II-1A Evidence)*   ***Key recommendations***   - Careful case selection and labour management in a modern obstetrical setting may achieve a level of safety similar to elective caesarean *(Level II-1 Evidence)* - In the absence of a contraindication to vaginal birth, a woman with a breech presentation should be informed of the risks and benefits of a TOL and elective caesarean, and informed consent should be obtained. A woman's choice of delivery mode should be respected. *(Level III-A Evidence)* - Hospitals offering a TOL should have a written protocol for eligibility and intrapartum management. *(Level III-B Evidence)* - Women with a contraindication to a TOL should be advised to have a caesarean. - Women choosing to labour despite this recommendation have a right to do so and should be provided the best possible in hospital care *(Level III-A Evidence)* | 67% |
| Mandruzzato 2010 | To offer recommendations based on available evidence for post-term pregnancy. | - Increased caesarean has only been observed in nulliparous, but not multiparous women - IOL versus expectant management: No differences in caesarean rates were reported in seven out of eight trials - A higher rate of neonatal mortality is reported in some, but not all studies***.*** - After 41 completed weeks, routine induction or expectant management can be offered ***(Level A Evidence)*** - Intrapartum fetal monitoring is recommended during post-term labor, irrespective of induction or spontaneous labour *(Level B Evidence)* | 42% |
| Sentilhes 2013 | Guidelines for clinical practice from the French College of Gynecologists and Obstetricians for delivery of women with a previous caesarean | - The expert advisory group considers TOLAC the preferred option in the great majority of cases *(professional consensus)* - Few individual clinical situations justify ERCS *(professional consensus)* - TOLAC and ERCS both present low risks of serious complications for mother and child. The risk-benefit ratio for the mother in both the short and long term favours TOLAC, but the short-term risk-benefit ratio for the child favours ERCS *(professional consensus)* - The choice of mode of birth must be shared by the woman and her doctor. If the woman desires a repeat CS after adequate information, discussion, and time to think about it, her preference should be honoured *(professional consensus)* | 38% |
| SOGC 2005 | To provide evidence-based guidelines for the provision of a trial of labour (TOL) after caesarean birth | - A woman with 1 previous transverse lower segment caesarean birth should be offered a TOL with appropriate discussion of maternal and perinatal risks and benefits. The process of informed consent with appropriate documentation should be an important part of the birth plan in a woman with a previous caesarean *(Level II-2B Evidence)* - The intention of a woman undergoing a TOLAC should be stated, and documentation of the previous uterine scar should be clearly marked on the prenatal record *(Level II-2B Evidence)* - For a safe LAC, a woman should deliver in a hospital where a timely caesarean is available *(Level II-2A Evidence)* - Each hospital should have a written policy in place regarding the notification and/or consultation for the physicians responsible for a possible timely caesarean *(Level III-B Evidence)* - Continuous electronic fetal monitoring of women attempting a TOLAC is recommended *(Level II-2A Evidence)* - Oxytocin augmentation is not contraindicated in women undergoing a TOLAC *(Level II-2A Evidence)* - Medical IOL with oxytocin may be associated with an increased risk of UR and should be used carefully after appropriate counselling *(Level II-2B Evidence)* - Prostaglandin E1 (misoprostol) is associated with a high risk of UR and should not be used as part of a TOLAC *(Level II-2A Evidence)* - A foley catheter may be safely used to ripen the cervix in a woman planning a TOLAC *(Level II-2A Evidence)* | 34% |
